# Structural and biochemical characterization of yeast Tcd enzymes installing the post-transcriptional modification ct^6^A in tRNA

**DOI:** 10.1093/nar/gkag376

**Published:** 2026-05-05

**Authors:** Julia Hirschmann, Rachel Sonntag, Matthias Heiss, Ewa Wegrzyn, Wolfgang Heinemeyer, Thomas Carell, Eva M Huber

**Affiliations:** Technical University of Munich, TUM School of Natural Sciences, Center for Functional Protein Assemblies, 85747 Garching, Germany; Technical University of Munich, TUM School of Natural Sciences, Center for Functional Protein Assemblies, 85747 Garching, Germany; Ludwig-Maximilians University Munich, Department of Chemistry and Pharmacy, 81377 München, Germany; Ludwig-Maximilians University Munich, Department of Chemistry and Pharmacy, 81377 München, Germany; Technical University of Munich, TUM School of Natural Sciences, Center for Functional Protein Assemblies, 85747 Garching, Germany; Ludwig-Maximilians University Munich, Department of Chemistry and Pharmacy, 81377 München, Germany; Technical University of Munich, TUM School of Natural Sciences, Center for Functional Protein Assemblies, 85747 Garching, Germany

## Abstract

Post-transcriptional modifications near the anticodon of transfer ribonucleic acids (tRNAs) ensure translation fidelity and accuracy. For instance, at position 37, the universally conserved and essential nucleoside *N^6^*-threonylcarbamoyladenosine (t^6^A) supports decoding of ANN triplets. In some organisms t^6^A is converted to cyclic t^6^A (ct^6^A), but only little is known about this ATP-dependent reaction and the corresponding threonylcarbamoyladenosine dehydratases (Tcds). We here show that yeast Tcds localize to the outer mitochondrial membrane and co-purify with tRNAs recognizing ANN codons. Depending on the number of *TCD* genes in the genome, the proteins form V-shaped hetero- or homodimers, of which at least one subunit binds and modifies tRNAs. The C-terminal, monomeric domain shares similarities with Cas9-endonucleases and assists tRNA recognition, while the N-terminal domain mediates dimerization and contains the active site. Structure-based mutagenesis and activity assays imply that yeast Tcds lack a catalytic cysteine and do not covalently bind their substrate as proposed for *Escherichia coli* TcdA.

## Graphical Abstract

**Graphical Abstract:**
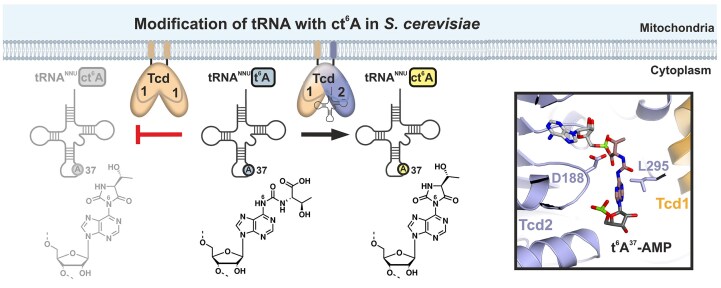

## Introduction

**Figure 1.**
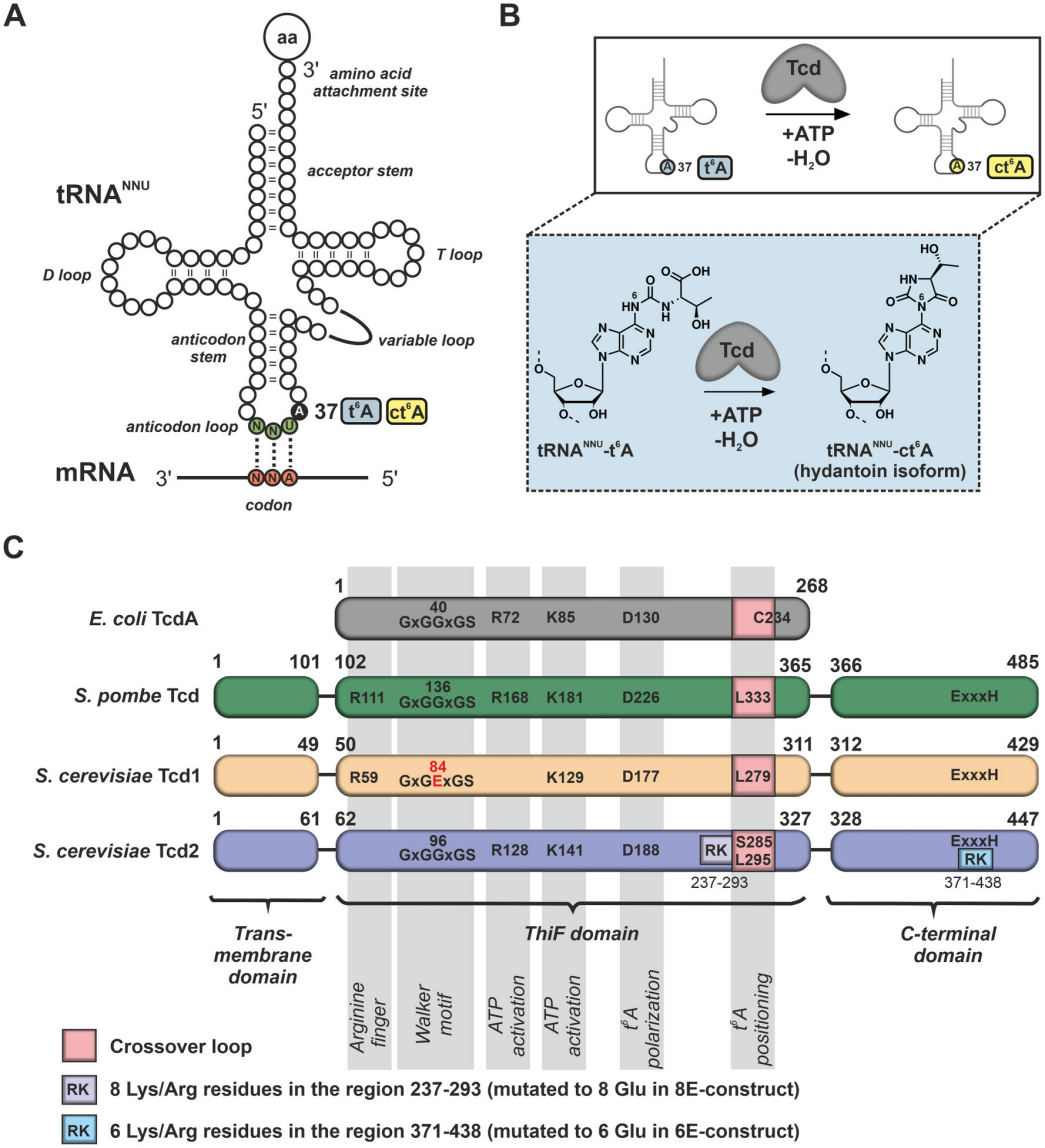
Activity and structure of tRNA modifying Tcd enzymes. (**A**) Schematic illustration of tRNA^NNU^ modified at position 37 next to the anticodon with t^6^A/ct^6^A. Both tRNA modifications support the decoding of ANN codons in mRNA at ribosomes. (**B**) Tcd enzymes catalyze the ATP-dependent cyclization from t^6^A to ct^6^A on tRNA. The tRNA has been created in BioRender. Huber, E. (2026) https://BioRender.com/h55raqy. (**C**) Schematic illustration of the domain architecture of Tcd enzymes. Amino acid numbers of domain borders are indicated. Key amino acid residues are labeled and their function is indicated in gray bars. Cysteine 234 of TcdA is not conserved in yeast Tcds. For details see Supplementary Fig. S1.

Like all nucleic acids, transfer ribonucleic acids (tRNAs) are decorated with post-transcriptional modifications. In fact, tRNAs are the most highly modified ribonucleic acids (RNAs) [1]. The decorations mostly occur in two hotspot regions and serve two major functions: Firstly, modifications of the tRNA core affect stability and tertiary structure; and secondly, modified nucleosides in the anticodon loop impact the function of tRNA as an adaptor molecule during translation [2]. In particular nucleoside variants close to the anticodon, at positions 34 and 37, ensure correct and efficient decoding of messenger RNAs (mRNAs) at ribosomes by modulating the structural flexibility of the anticodon loop and improving base stacking [3–5]. For instance, *N*^6^-threonylcarbamoyladenosine (t^6^A), a nucleoside often found at position 37 of tRNA, helps to decode ANN triplets [6, 7] and hence safeguards translation [8, 9] (Fig. 1A). Due to the crucial function of t^6^A, any disruption of its biosynthesis, starting from ${\rm L}$-threonine and carbonate [10], is lethal or causes severe diseases like the Galloway–Mowat syndrome [11–13]. t^6^A is universally conserved in all three kingdoms of life [10] and, as an example for an amino acid containing noncanonical RNA base, considered as a fossil of a former RNA-peptide world [14, 15]. In 2013, a cyclic, nonessential but more effective form of t^6^A, termed ct^6^A, was discovered at position 37 of tRNA in some bacteria, fungi, plants, and protists [16] (Fig. 1A). Although initially ct^6^A was reported to adopt an oxazolone structure [16], later the hydantoin isoform was identified and visualized in tRNA [17, 18]. Starting from t^6^A, ct^6^A is installed in an ATP-dependent reaction by enzymes termed threonylcarbamoyladenosine dehydratases (Tcds) [16] (Fig. 1B). So far, members of this enzyme family have been studied from *Escherichia coli* [16, 19, 20] and *Saccharomyces cerevisiae* [16]. While *E. coli* encodes only one Tcd enzyme, termed TcdA (formerly CsdL), *S. cerevisiae* encodes two homologues, Tcd1 and Tcd2, the functional importance of which was unclear at the beginning of this study. Furthermore, each of the two *S. cerevisiae* homologues features an extended C-terminal domain of unknown function compared to *E. coli* TcdA (Fig. 1C). Based on the available structural and biochemical data for *E. coli* TcdA [19, 20], we here set out to study the increased complexity of yeast Tcd enzymes in order to contribute to a better understanding of ct^6^A formation in general. By producing Tcd enzymes from *S. cerevisiae* and *Schizosaccharomyces pombe* heterologously in *E. coli*, we found that in organisms which encode only one Tcd variant, e.g. *E. coli* or *S. pombe*, the Tcd enzyme forms a homodimer with probably two functional active sites. By contrast, in *S. cerevisiae* the two encoded Tcd homologues assemble into a heterodimer of which only Tcd2 appears to have retained tRNA binding capacity. Structural data reveal a V-shape for yeast Tcd enzymes and a distant relationship of their C-terminal domain to Cas9 restriction endonucleases. A combination of yeast genetics, point mutagenesis, and *in vivo* activity assays complements the structural data and suggests for the C-terminal domain a function in tRNA recognition.

## Materials and methods

### Key methods relevant for main figure experiments

#### Protein overproduction in *E. coli*

For large scale expression freshly transformed SoluBL21 (DE3) cells (Supplementary Table S11) were grown in 50 ml overnight culture at 37°C while shaking at 120 revolutions per minute (rpm). The next day, the main culture (up to 8 L in 2 L flasks) was inoculated in a ratio of 1:100 and incubated at 37°C and 120 rpm. Once an OD_600_ of 0.5–0.8 was reached, the main culture was cooled to 20°C expression temperature. To this end, the 2 L flasks were cooled for 30 min at 4°C. Subsequently, isopropyl β-D-1-thiogalactopyranoside (IPTG) was added to a final concentration of 1 mM to induce heterologous gene expression, and the main culture was incubated for 16 h at 20°C and 120 rpm. Thereafter, the cells were harvested by centrifugation at 4000 × *g* for 20 min, washed with an isotonic solution of sodium chloride [0.9% (w/v) NaCl], and frozen at −20°C.

#### Protein purification

Cells were homogenously dissolved in buffer A (specific buffer composition for individual protein constructs can be found in Supplementary Table S17), and 1 mg DNase and 5 mg protease inhibitor were added. The solution was sonicated (5 min, 70% amplitude, and 1 s on/off), centrifuged (21 000 × *g* for 20 min at 4°C) and the resulting supernatant was applied to a nickel (Ni) affinity chromatography column (HisTrap HP 5 ml, Cytiva, Marlborough, United States) pre-equilibrated with 5 column volumes (CV) buffer A containing 20 mM imidazole. Unbound protein was removed by washing with 5 CV buffer A. The target protein was eluted using a gradient from 0% to 100% over 10 CV buffer B comprising 500 mM imidazole (Supplementary Table S17). Fractions containing the protein of interest were pooled and supplemented with either His_6_-TEV protease (self-made) or His_6_-SUMO protease (self-made) or both to cleave the tag(s). The mixture was dialyzed overnight at 4°C in buffer C (Supplementary Table S17).

The digested protein was again loaded on a HisTrap column, pre-equilibrated with 5 CV buffer A. In this step, the cleaved protein was separated from the uncleaved protein, tag, and protease. The flow-through was collected and concentrated with a 50 000 MWCO centrifugal filter unit. The protein was loaded on a Superdex 200 pg 16/600 or a Superdex 200 Increase 10/300 GL gel filtration column equilibrated with buffer D (Supplementary Table S17). The desired protein fractions were concentrated, flash-frozen in liquid nitrogen and stored at −80°C.

#### Protein crystallization

All crystallization trials were performed using the sitting drop vapor diffusion technique. SpTcd^366-485^ was crystallized at 20°C from a 1:1 mixture of 0.2 µl protein solution (10 mg/ml in 20 mM Tris–HCl pH 7.5, 150 mM NaCl, and 3 mM DTT) and 0.2 µl of reservoir solution containing 0.2 M NaCl, 0.1 M citrate pH 5.5, 1 M (NH_4_)_2_HPO_4_, final pH 7.6. A 1:1 mixture of the reservoir solution and 70% (v/v) glycerol was used as a cryoprotectant prior to vitrifying the crystals in liquid nitrogen. Crystals of Tcd1^50-429^ were grown at 4°C from 0.2 µl protein (20 mg/ml in 20 mM Bis–Tris/HCl pH 6.5, 150 mM NaCl, 10% (v/v) glycerol, 5 mM MgCl_2_, 3 mM DTT, and 3 mM AMP) mixed with 0.1 µl of reservoir solution containing 1.98 M sodium malonate pH 7.0. A 1:1 mixture of reservoir and 70% (v/v) glycerol was used as a cryoprotectant.

#### X-ray data collection and structure determination

Diffraction data were collected at the beamline X06SA at the Swiss Light Source (Paul Scherrer Institute, Villigen, Switzerland, λ = 1 Å). Reflection intensities were evaluated with XDS [21] and processed with XSCALE [21]. By determining the Matthews coefficient, the number of protein molecules in the asymmetric subunit was estimated to facilitate phasing. The structure of SpTcd^366-485^ was solved by *ab initio* phasing with Arcimboldo lite [22] within the CCP4 suite [23]. The model was adapted with Coot [24] and iteratively refined with REFMAC5 [25] or phenix.refine [26]. The statistics are summarized in Supplementary Table S1. The structure of Tcd1^50-429^ was solved with Phaser [27] using its AlphaFold 2 prediction [28, 29] as a search model. The statistics are summarized in Supplementary Table S1.

#### Yeast mitochondria isolation

To extract mitochondria from *S. cerevisiae*, a liquid culture of 5 ml was grown overnight at 30°C in YPD (Yeast Peptone Dextrose) media to saturation. The following morning, a liquid culture of 50 ml of YPD was inoculated to an optical density (OD) at λ = 600 nm (OD_600_) of 0.5–0.6. After reaching the log phase, at an OD_600_ of 1.5 to 2.0, an amount of OD_600 _= 20 of yeast cells was harvested. Yeast mitochondria isolation and purification were performed according to the manual of the Abcam Yeast Mitochondria Isolation Kit [Abcam (Cambridge, GB)]. The resulting pellet was resuspended in 50 µl of storage buffer. Each sample was divided into two, and one part was incubated with 200 µl/ml proteinase K for 30 min on ice, while the other was left untreated. Subsequently, the samples were analyzed by western blotting.

#### Western blotting

Equal amounts of isolated mitochondria, treated with proteinase K or not, were resolved by SDS–PAGE (sodium dodecyl sulfate/polyacrylamide gel electrophoresis) and transferred on a nitrocellulose membrane (GE Healthcare, Chalfont St. Giles, GB) using a semi-dry blotting transfer system (Biometra Fastblot, Analytik Jena, Jena, DE; 1 h at 100 mA). After blotting, the membranes were stained with Ponceau, destained with water and incubated in 5% (w/v) skim milk in TBS-T (Supplementary Table S13) for 1 h (western blot shown in main figure) or 3 h (western blots shown in Supplementary Information) at room temperature (RT).

After blocking of α-Myc/α-HA blots, the first antibody (either α-Myc or α-HA) was added according to SupplementaryTable S14, and the membranes were incubated overnight at 4°C on an orbital shaker. After washing six times 5 min with TBS-T, the membranes were exposed to 5% (w/v) skim milk in TBS-T supplemented with horse radish peroxidase (HRP) labeled secondary antibody (AB_2536527, Invitrogen, Carlsbad, United States; Supplementary Table S14) for 60 min on an orbital shaker at RT. Afterwards, the membranes were washed three times with TBS-T for 5 min and then three times with TBS for 5 min.

After blocking of α-His blots, an HRP-conjugated anti-His_6_ antibody (HRP-66005, Proteintech, Rosemont, United States) was added according to Supplementary Table S14, and the membrane was incubated on a shaker at 4°C overnight. The membrane was washed once with TBS, twice with TBS-T, and thrice with TBS for 5 min each.

For Western blots against *S. cerevisiae* VDAC-1 (Uniprot ID: P04840) and actin (Uniprot ID: P60010), membranes were incubated after the blocking step with the corresponding antibody (Supplementary Table S14) in 5% (w/v) skim milk overnight at 4°C on an orbital shaker. After washing six times 5 min with TBS-T, the membranes were exposed to 5% (w/v) skim milk in TBS-T supplemented with HRP-labeled secondary antibody [A9044, Merck (Darmstadt, DE), Supplementary Table S14] for 120 min on an orbital shaker at RT. Afterwards, the membranes were washed three times with TBS-T for 5 min and then three times with TBS for 5 min.

Finally, all membranes were immersed in WesternBright ECL-spray (Advansta, San Jose, United States), and the emitted light was monitored with the imaging devices ImageQuant LAS 4000 (Cytiva, Marlborough, United States) or Vilber Fusion FX (Vilber, Marne-la-Vallée, FR).

#### Estimation of molecular weights by size-exclusion chromatography

Size-exclusion chromatography columns were calibrated by determining the retention volumes of selected marker proteins (Supplementary Fig. S28A and C). Catalase (1.8 mg, 240 kDa), aldolase (6.3 mg, 158 kDa), conalbumin (7.0 mg, 75 kDa), and ovalbumin (4.0 mg, 43 kDa) were individually applied to a Superdex 200 pg 16/600 column at a flow rate of 1 ml/min using 20 mM Tris–HCl (pH 7.5), 100 mM NaCl, and 10% (v/v) glycerol as the mobile phase. For calibration of the Superdex 200 Increase 10/300 GL column, ferritin (0.15 mg, 440 kDa), aldolase (1.5 mg, 158 kDa), conalbumin (1.5 mg, 75 kDa), and ovalbumin (1.2 mg, 43 kDa) were applied separately at a flow rate of 0.8 ml/min in 20 mM Tris–HCl (pH 7.5), 100 mM NaCl, and 5 mM dithiothreitol (DTT). Retention volumes were plotted against the logarithm of the molecular weight of the respective marker proteins and fitted with a linear fit in Origin (Supplementary Fig. S28B and D). Please note that size exclusion chromatography separates proteins based on their hydrodynamic radius, which depends on size and shape of the macromolecule and thus gives only a rough estimation of the molecular weight or oligomeric state.

#### Native PAGE/Electrophoretic mobility shift assay (EMSA)

Five micrograms of purified proteins were mixed with binding buffer (5×), loading buffer (10×) (Supplementary Table S18), and optionally *in vitro* transcribed tRNA. The total volume of each sample was adjusted to 20 µl using sample buffer (buffer D, Supplementary Table S17). Samples were loaded on a 5% precast TBE gel (Bio-Rad, Hercules, United States), and the gel was run at 15 mA for 1 h at 4°C in TBE running buffer (1×). Afterwards, the gel was stained with 1:10 000 SYBR Gold (Invitrogen, Carlsbad, United States) in TBE Buffer (1×) for 30 min while shaking in the dark. RNA bands were visualized under UV light (λ = 365 nm). Subsequently, the gel was stained with Coomassie Blue and decolorized with the destaining solution to visualize the corresponding protein bands.

#### *In vivo* activity assays

Single clones of *S. cerevisiae* transformed with an pBEVY-GL^Amp, Leu^ based expression vector were used to inoculate 25 ml of complete minimal (CM)^Leu−^ dropout medium (Supplementary Table S16) which was incubated at 30°C and 120 rpm for 2 days. Cultures were harvested by centrifugation at 5 000 rpm for 5 min, washed with sterile ddH_2_O, transferred to 25 ml of selection medium containing galactose for protein expression, and subsequently incubated at 30°C and 120 rpm for 2 days.

Single clones of *S. cerevisiae* transformed with an pBEVY-L^Amp, Leu^ based expression vector were used to inoculate 25 ml of CM^Leu−^ dropout medium which was then incubated at 30°C and 120 rpm for 2 days. Untransformed strains were grown in 25 ml of YPD medium and incubated at 30°C and 120 rpm overnight. Two milliliters of each culture was harvested by centrifugation, and cells were lysed by the addition of TRIzol reagent (Invitrogen, Carlsbad, United States). RNA was purified according to the manufacturer’s protocol. Small RNAs were further purified by LiCl precipitation. In brief, 1 volume of 4 M LiCl was added, and the mixture was incubated at −20°C overnight. Samples were centrifuged at 14 000 × *g* for 15 min at 4°C, and the supernatant, containing small RNAs, was collected. One microliter of GlycoBlue (Invitrogen, Carlsbad, United States), 0.1 volumes of 5 M NH_4_OAc, and 2.5 volumes of 100% (v/v) EtOH were added. The samples were incubated for 1 h at −20°C and centrifuged at 12 000 × *g* for 30 min. The supernatant was discarded, and the pellet was washed with 75% (v/v) EtOH and centrifuged again at 12 000 × *g* for 5 min at 4°C. The supernatant was discarded again, and the pellet was dried for 5 min before dissolving in H_2_O. After digestion, the nucleosides were quantified by LC–MS/QQQ (see below).

#### Quantification of t^6^A and ct^6^A

##### Digestion of RNA to nucleosides

100 ng to 1 µg of pre-purified small RNAs (20 µl of each) were digested to single nucleosides by using 2 U benzonase nuclease (>90% purity, Merck, Darmstadt, DE) 0.2 U phosphodiesterase I (VWR), and 2 U alkaline phosphatase (QuickCIP, NEB) in MgCl_2_ (1 mM) containing NH_4_OAc (pH 4.9, 5 mM) buffer. Acidic buffer was chosen over the standard Tris (pH 8) buffer due to better stability of ct^6^A in acidic conditions. Test digestions were conducted to make sure digestion and dephosphorylation still work. In a final volume of 30 µl 1 µg pentostatin, 5 µg of THU, and a final concentration of 10 µM BHT were added to each reaction for protection against degradation of the released nucleosides. After incubation for 2 h at 37°C, 20 μl of LC-MS buffer A (see below) was added to the mixture and then filtered through 0.2 µm Supor Natural PP filters (Pall Corporation, AcroPrep Advance350, 96 well-plate) at 3000 × *g* and 4°C for 30 min before measurement by LC–MS/QQQ.

##### LC–MS/QQQ analysis of nucleosides

For quantitative mass spectrometry an Agilent 1290 Infinity equipped with a variable wavelength detector (VWD) combined with an Agilent Technologies G6490 Triple Quad LC/MS system with electrospray ionization (ESI-MS, Agilent Jetstream) was used. Operating parameters: positive-ion mode, cell accelerator voltage of 5 V, N_2_ gas temperature of 120°C and N_2_ gas flow of 11 l/min, sheath gas (N_2_) temperature of 280°C with a flow of 11 l/min, capillary voltage of 3000 V, nozzle voltage of 0 V, nebulizer at 60 psi, high-pressure RF at 100 V, and low-pressure RF at 60 V. The instrument was operated in dynamic MRM mode. For separation an Uptisphere C18-HDO column (3.0 μm, 150 × 2.1 mm from Interchim, UP3HDO-150/021) was used. Running conditions were 35°C and a flow rate of 0.35 ml/min in combination with a binary mobile phase of 5 mM NH_4_OAc aqueous buffer A, brought to pH 4.9 with glacial acetic acid (200 μl/l), and an organic buffer B of acetonitrile (Roth, Ultra LC-MS grade, purity ≥99.98) acidified with 0.075% formic acid (187.5 µl per 2.5 l). The gradient started at 100% solvent A for 0.5 min, followed by an increase of solvent B to 10% over 5.5 min. From 6.0 to 8.5 min, solvent B was increased to 20% then to 80% in 1 min and maintained at 80% for 1.5 min before returning to 100% solvent A in 0.5 min and a 2.2 min re-equilibration period. Of each sample 10 µl were co-injected with 1 µl of stable isotope labeled internal standard (ISTD), which was aspirated automatically before each injection from the instrument itself. The ISTD consisted of a mix containing all relevant isotopically labeled nucleosides, including C, U, G, A, and t^6^A. The labeling was as follows: ^13^C_9_-Cytidine, ^13^C_9_-Uridine, ^13^C_10_-Guanosine, ^13^C_10_-Adenosine (all from Silantes), and ^13^C_5_-threonylcarbamoyladenosin (synthesized in the lab of Prof. Thomas Carell, isotope label in functional group). This standardized ISTD mix was used for all analyses, ensuring exact co-injection of 1 µl per sample. For both the quantification of t^6^A and ct^6^A the ISTD of t^6^A was used. The sample data were analyzed by the quantitative MassHunter Software from Agilent using the integrated calibration function. The calibration solutions ranged from 0.05 to 100 pmol for each canonical nucleoside and from 0.002 to 5 pmol for each modified nucleoside (12 calibration levels, 1:2 dilution). Retention times and mass transitions (including collision energies) for C, U, G, A, t^6^A, and ct^6^A were determined and optimized using chemically synthesized standards. These parameters were consistently applied for the analysis of all calibration levels and experimental samples. Exemplary UV- and MS-chromatograms of calibration level 10, including determined retention times and mass transitions from precursor to product ion are shown in Supplementary Fig. S30.

##### Calculation for modification per tRNA molecule

The measured molar amount of all canonical nucleosides (pmol) was summed and then divided by 70 (average number of canonical nucleosides per tRNA molecule) to obtain the molar amount of tRNA. The measured amount of modification was then divided by the calculated molar amount of tRNA to obtain the number of modifications per tRNA molecule.


\begin{eqnarray*}
\# mod\ per\ \textit{tRNA} = \frac{{{{n}_{\textit{modification}}}}}{{{{n}_{\textit{tRNA}}}}}
\end{eqnarray*}

### Additional materials and methods

#### Cloning and mutagenesis of constructs for protein production in *E. coli*

##### S. pombe TCD (Uniprot ID: O13861, sequence version 1 annotated before April 2025)

Genomic DNA from *S. pombe* served as a template for amplifying gene fragments coding for Tcd^102-485^ (primer #1 and #2, Supplementary Table S5) and Tcd^366-485^ (primer #3 and #2, Supplementary Table S5), respectively. The amplified sequences were cloned into expression vectors using the restriction enzymes *Bgl*II-HF and *Pst*I-HF. The resulting plasmids, pET28b^Kan^-His_6_-SUMO-*TCD^304-1458^* and pRSET A^Amp^-His_6_-TEV-*TCD^1096-1458^* (Supplementary Table S6), were confirmed by sanger sequencing (Eurofins Genomics, Ebersberg, DE) and served as templates for Q5 mutagenesis using primers #4 and #5 (Supplementary Table S5).

##### S. cerevisiae TCD1 (Uniprot ID: P38756)

*TCD1^148-1290^* coding for Tcd1^50-429^ was amplified from *S. cerevisiae* genomic DNA (primer #6 and #7, Supplementary Table S5) and cloned into the expression vector pRSET A^Amp^-His_6_-TEV by the restriction enzymes BamHI-HF and PstI-HF yielding pRSET A^Amp^-His_6_-TEV*-TCD1^148-1290^* (Supplementary Table S6). This vector served as a template for Q5 mutagenesis with oligonucleotides #8–11 (Supplementary Table S5), resulting in pRSET A^Amp^-His_6_-TEV*-TCD1^148-1290^-E84G* and pRSET A^Amp^-His_6_-TEV*-TCD1^148-912^* (Supplementary Table S6). Additionally, the same plasmid was used as a template for the amplification of the His_6_-TEV*-TCD1^148-1290^* cassette with oligonucleotides #12 and #13 (Supplementary Table S5). The amplified His_6_-TEV*-TCD1^148-1290^* cassette was then inserted into the multiple cloning site (MCS) 2 of the plasmid pACYCDuet-1^Cm^ via NdeI-HF and KpnI-HF. The resulting vector pACYCDuet-1^Cm^-His_6_-TEV-*TCD1^148-1290^* (Supplementary Table S6) served for the co-expression with *TCD2^184-1344 ^*(next paragraph) and was further modified for the overexpression of tRNAs. Upstream of the *T7* promoter of MCS1, the EcoNI restriction site was mutated to an *Aat*II recognition site by Quikchange site-directed mutagenesis (primer #14 and #15, Supplementary Table S5). In the following, different synthetic tRNA gene fragments (Supplementary Table S7) under control of the *lpp* promoter and *rrnc* terminator were amplified and inserted via restriction cloning (*Aat*II and *Hind*III-HF; primer #16 and #17) or standard Gibson assembly (primer #18–21, Supplementary Table S5) [30], replacing MCS1 and its regulatory elements. This way pACYCDuet-1^Cm^-tRNA^Thr(UGU)^–His_6_-TEV-TCD1^148-1290^* and pACYCDuet-1^Cm^-tRNA^Arg(CCU)^–His_6_-TEV-TCD1^148-1290^* constructs were created (Supplementary Table S6).

##### S. cerevisiae TCD2 (Uniprot ID: P36101)

*TCD2^184-1344^* coding for Tcd2^62-447^ was amplified from *S. cerevisiae* genomic DNA (primer #22 and #23, Supplementary Table S5) and cloned into the expression vector pRSET A^Amp^-His_6_-TEV by the restriction enzymes BglII/BamHI-HF and PstI/PstI-HF yielding pRSET A^Amp^-His_6_-TEV*-TCD2^184-1344^* (Supplementary Table S6). Later, a synthetic gene fragment *TCD2^184-1344^* coding for *S. cerevisiae* Tcd2^62-447^ but adapted to codon-usage of *E. coli* (Supplementary Table S8) was ordered from Eurofins Genomics (Ebersberg, DE) and subcloned by the company with the restriction enzymes BamHI-HF and PstI-HF in MCS1 of a modified pETDuet-1^Amp^ vector, carrying a His_6_-SUMO cassette in front of MCS1 (resulting in pETDuet-1^Amp^-His_6_-SUMO*-TCD2^184-1344^*, Supplementary Table S6). Similarly, a pETDuet-1^Amp^-His_6_-SUMO*-TCD2^184-1344^-14E* construct was ordered, in which 14 Arg or Lys codons were replaced by Glu (Supplementary Table S8). Both plasmids served as templates for the creation of vectors pETDuet-1^Amp^-His_6_-SUMO*-TCD2^184-1344^-6E* and pETDuet-1^Amp^-His_6_-SUMO*-TCD2^184-1344^-8E* by Gibson assembly using primers #24–27 and #28–31 (Supplementary Table S5), respectively. Additional combinations of single point mutants in *TCD2^184-1344^* were created by Q5 mutagenesis using primers *#*32–65 (Supplementary Table S5).

The vector pETDuet-1^Amp^-His_6_-SUMO*-TCD2^184-1344^* and its mutant variants served for co-expression experiments with pACYCDuet-1^Cm^-tRNA^Thr(UGU)^–His_6_-TEV-TCD1^148-1290^* or pACYCDuet-1^Cm^-tRNA^Arg(CCU)^–His_6_-TEV-TCD1^148-1290^*.

To confirm that tagging of Tcd2^62-447^ is sufficient for the purification of the Tcd1-Tcd2-tRNA complex, *TCD1^148-1290^* was additionally inserted via standard Gibson assembly (using primer #66–69, Supplementary Table S5) in MCS2 of the vector pETDuet-1^Amp^-His_6_-SUMO*-TCD2^184-1344^*, generating pETDuet-1^Amp^-His_6_*-*SUMO-*TCD2^184-1344^–TCD1^148-1290^*(Supplementary Table S6).

##### E. coli TcdA (Uniprot ID: Q46927)

Genomic DNA from *E. coli* served as template for amplifying *tcdA* with oligonucleotides #70 and #71 (Supplementary Table S5). The resulting fragment was cloned into MCS1 of pETDuet-1^Amp^-His_6_-SUMO via standard Gibson assembly using primer #72 and #73 (Supplementary Table S5), allowing the production of N-terminally His_6_-SUMO-tagged full-length TcdA protein from pETDuet-1^Amp^-His_6_-SUMO-*tcdA* (Supplementary Table S6).

#### Cloning and mutagenesis of constructs for protein production in *S. cerevisiae*

For homologous, soluble protein production in *S. cerevisiae*, the coding sequence for His_6_-TEV-*TCD1^148-1290^* was amplified (primer #74 and #7, Supplementary Tables S5 and S9) and inserted by BamHI-HF and PstI-HF restriction enzymes into the vector pBEVY-L^Amp, Leu^.

Next, synthetic gene fragments coding for the His_6_-SUMO^star^ tag (LifeSensors Inc.; Supplementary Table S8) and the genomic *S. cerevisiae TCD2^184-1344^* sequence were amplified and fused by PCR (primer #75–78, Supplementary Table S9) to generate a His_6_-SUMO^star^-*TCD2^184-1344^* cassette. In the following, this fragment was inserted by the restriction enzymes KpnI-HF and SacI-HF in the pBEVY-L^Amp, Leu^-His_6_-TEV-*TCD1^148-1290^* vector to produce pBEVY-L^Amp, Leu^-His_6_-TEV-*TCD1^148-1290^*–His_6_*-*SUMO^star^-*TCD2^184-1344^* (Supplementary Table S10). Additionally, a pBEVY-L^Amp, Leu^-His_6_-TEV-*TCD1^148-1290^*–His_6_*-*TEV-*TCD2^184-1344^* plasmid (Supplementary Table S10) was created. To this end, the *TCD2^184-1344^* sequence was amplified (primers #79 and #78, Supplementary Table S9) and cloned with KpnI-HF and SacI-HF in pBEVY-L^Amp, Leu^-His_6_-TEV-*TCD1^148-1290^*.

For the *in vivo* activity assay, full-length *TCD1^1-1290^* and *TCD2^1-1344^* genes were expressed without any affinity tags from a pBEVY-GL^Amp, Leu^-*TCD1^1-1290^*-*TCD2^1-1344^* plasmid (Supplementary Table S10). For construction of this plasmid, the coding sequences of *TCD1^1-1290^* and *TCD2^1-1344^* as well as the target vector backbones were amplified by PCR (oligonucleotides #80–87, Supplementary Table S9) and put together step by step via standard Gibson assembly. Single point mutations in either *TCD1* or *TCD2* were introduced via Q5 site-directed mutagenesis following the instructions of the NEBase Changer tool. The corresponding primers #88–105 are listed in Supplementary Table S9. To verify the expression of the different mutants *in vivo* by western blotting (Supplementary Fig. S22), the same constructs were also created as C-terminal His_7_ fusions (using primers #106–109, Supplementary Table S9).

All cloning steps were performed in *E. coli* XL1 blue cells (Supplementary Table S11) and the correct insertion of all gene sequences was confirmed by Sanger sequencing (Eurofins Genomics, Ebersberg, DE).

#### Transformation of *S. cerevisiae*

*S. cerevisiae* (Supplementary Table S11) was transformed according to the lithium acetate (LiAc) method by Gietz and Woods [31]. A pinhead size of cells was resuspended in 1 ml of sterile ddH_2_O, centrifuged at 13 000 rpm for 30 s and washed with 1 ml of TE/LiAc/H_2_O (1:1:8; v/v/v, Supplementary Table S12). After another centrifugation step, the cells were taken up in 50 µl of TE/LiAc/H_2_O, and 5 µl of DNA of interest (100 ng/µl) and 3.5 µl of salmon testes DNA (10 mg/ml) were added. Next, 300 µl of TE/LiAc/PEG_3350_ (1:1:8; v/v/v, Supplementary Table S12) were supplemented and the mixture was incubated at 30°C for 30 min. Subsequently, the cells were heat-shocked at 42°C for 20 min, centrifuged for 2 min at 2 000 rpm and resuspended in 100 µl of sterile ddH_2_O. Transformed cells were plated on YPD or CM^Leu−^ agar plates containing the appropriate selection marker and stored at 30°C for 24 to 72 h.

#### Isolation of genomic DNA from *S. cerevisiae*

Cells were scraped off an agar plate, dissolved in 500 µl of ddH_2_O, and centrifuged for 30 s at 16 000 rpm. The cell pellet was resuspended in 200 µl of breaking buffer [2% (v/v) Triton X, 10 mM Tris–HCl pH 8.0, 100 mM NaCl, 1 mM ethylenediaminetetraacetic acid (EDTA), and 10 µg/µl RNase]. Two hundred microliters of phenol–chloroform–isoamyl alcohol (25:24:1) and approximately 200 µl of glass beads were added. Cells were lysed by vortexing for 2 min. After centrifugation at 16 000 rpm for 5 min, 100 µl of the aqueous phase (supernatant) was transferred in a new tube, mixed with 10 µl of sodium acetate (3 M, pH 6.0) and 280 µl of ethanol_absolute_ and stored at −80°C for 10 min to precipitate nucleic acids. After centrifugation for 5 min at 14 000 rpm, the supernatant was discarded and the pellet was washed with 300 µl of 70% (v/v) ice-cold ethanol. Following an additional centrifugation step (5 min, 14 000 rpm), the pellet was dried at room temperature and finally taken up in 100 µl of TE buffer supplemented with 1 µl of RNase (10 μg/μl).

#### Generation of knockout strains

The *tcd2Δ::KanMX4* strain (BY4741 background, Supplementary Table S11) was obtained from the Euroscarf gene deletion library. The *tcd1Δ::NatMX6* strain (BY4742 background) was produced by homologous recombination. To this end, the *NatMX6* marker gene was amplified from plasmid pFA6a-*NatMX6* (Supplementary Table S10) using primers #110 and #111 (Supplementary Table S9). As these oligonucleotides possess 40–50 bps overhangs complementary to the flanking regions of the yeast *TCD1* gene, the resulting PCR product contained 5′ and 3′ ends that were homologous to the genomic DNA surrounding the yeast *TCD1* gene. After transformation of the PCR product in BY4742, the transformed cells were plated on nourseothricin containing agar plates (100 μg/ml) to select for clones in which homologous recombination was successful.

The *tcd1Δ::NatMX6 tcd2Δ::KanMX4* double knockout strain was generated by mating the two single knockout strains *tcd2Δ::KanMX4* and *tcd1Δ::NatMX6*. The resulting heterozygous diploid was sporulated and after tetrad dissection double mutant spore clones were identified by selection against both nourseothricin and G418 (geneticin; 300 µg/ml) [32].

A *tcd1ΔCterm::KanMX4 tcd2ΔCterm::NatMX6* mutant in the BY4741 strain background was created by consecutively replacing the C-terminal domains of *TCD1* and *TCD2* with the *KanMX4* and the *NatMX6* cassette, respectively. For this purpose, primers #112 and #113 (Supplementary Table S9) and the template plasmid pYM14 containing the *KanMX4* marker (Supplementary Table S10) were used to generate a PCR fragment for the first transformation. The resulting *tcd1ΔCterm::KanMX4* strain was then transformed with a fragment resulting from PCR with primers #114 and #115 (Supplementary Table S9) and the pFA6a-*NatMX6* template plasmid (Supplementary Table S10). The correct integration of all markers was verified by PCR using primer pairs that bind upstream of the deletion and in the marker cassette, respectively (primer #116–121; Supplementary Table S9), and sequencing.

#### Tagging of *TCD1* and *TCD2* in yeast

*TCD1* and *TCD2* were C-terminally tagged in *S. cerevisiae* with either a HA_6_ tag or a Myc_9_ tag. Primers #122–125 (Supplementary Table S9) that overlap with the chromosomal region flanking the target site of the tag (excluding the stop codon) were used to amplify the tag cassette sequence on either pYM14 (HA_6_ tag, *KanMX4* selection marker) or pYM21 (Myc_9_ tag, *NatNT2* selection marker) (Supplementary Table S10) [33]. Each PCR product was transformed into wild-type BY4741 cells via the LiAc method. The transformed cells were resuspended in 1 ml of YPD medium and incubated at 30°C for 2 to 3 h. Depending on the cassette used, the cells were plated on YPD plates containing G418 (300 µg/ml) or nourseothricin (100 μg/ml) for selection and then incubated at 30°C for 3 days. Single colonies were streaked out on YPD plates containing the appropriate selection marker and incubated at 30°C for 1 day. Finally, genomic DNA was isolated and a control PCR with primers #118–121 was performed, followed by sequencing, to verify the correct insertion of the tag cassette.

#### *In vitro* transcription (IVT)

The template DNA, coding for a T7 promoter and the tRNA gene, was ordered as a linear gene strand from Eurofins Genomics (Ebersberg, DE) (Supplementary Table S15). The reaction was performed according to the instructions of the AmpliScribe T7 High Yield Transcription Kit (Lucigen, Middelton, United States).

#### Test expression experiments in *E. coli*

*E. coli* SoluBL21 (DE3) cells were transformed with the respective expression vector and cultured in LB medium containing antibiotics. Liquid pre-cultures were grown overnight at 37°C and 120 rpm. The next day, 1 ml of pre-culture was used to inoculate 50 ml of fresh LB medium, and the main culture was grown at 37°C and 120 rpm until an OD_600_ of 0.5 was reached. Cells were induced with 1 mM IPTG and incubated overnight at 20°C. Cells were harvested by centrifugation and lysed via sonication. The cell lysate was cleared from cell debris by centrifugation, and the protein of interest was purified using Ni-nitrilotriacetic acid (NTA) Spin Columns (Qiagen, Hilden, DE) according to the manufacturer’s protocol. Collected samples were analyzed on a 12% SDS–PAGE.

#### Protein overproduction in *S. cerevisiae* for subsequent isolation

*S. cerevisiae* transformed with an pBEVY-L^Amp, Leu^ based expression vector was used to inoculate a pre-culture. After incubation overnight at 30°C, 120 rpm in YPD medium, 3 l of CM^Leu−^ dropout media were inoculated in a ratio of 1:100, and the cells were grown at 30°C for 28 h. Subsequently, the cells were harvested at 5000 × *g* for 20 min by centrifugation, washed with ddH_2_O, and stored at −20°C.

#### Mass spectrometry analysis of proteins

##### ESI–MS analysis

Purified proteins were analyzed for their molecular weight on a Synapt XS mass spectrometer equipped with an electrospray ion source and a time-of-flight mass analyzer (*m/z*-range of 400–4000, Waters Corp.). Liquid samples (concentration: 0.1 mg/ml; injection volume: 2 µl) were loaded on a C4 column coupled to an ACQUITY HPLC system (Waters Corp.). An elution gradient of 0%–95% acetonitrile was applied. Mass data were analyzed by MassLynx software (Waters Corp.).

##### Peptide mass fingerprinting

To identify proteins in gel fragments spots were excised with a pipette tip, washed twice with 10 mM NH_4_HCO_3_ for 10 min each, followed by a wash with acetonitrile:10 mM NH_4_HCO_3_ (1:1, v/v, 10 min) and removal of all liquid. For reduction, 100 µl of buffer containing 80 mM DTT, 0.5 mg guanidine hydrochloride, 0.8 mM EDTA, and 0.1 mM Tris–HCl (pH 8.2) were added, and samples were incubated at 37°C for 30 min while shaking. Subsequently, 10 µl of 0.5 M iodoacetamide in 0.1 M Tris–HCl (pH 8.2) were added, and samples were incubated in the dark for 15 min. The supernatant was removed, and gel pieces were stored in 5 µl of 1 mM β-mercaptoethanol and 100 µl of 10 mM NH_4_HCO_3_ for 5 min while shaking. After removal of the liquid, gel pieces were washed once with acetonitrile:10 mM NH_4_HCO_3_ (1:1, v/v, 10 min), followed by 10 mM NH_4_HCO_3_ for 10 min. This wash sequence was repeated once, and finally a 10 min wash with acetonitrile:10 mM NH_4_HCO_3_ (1:1, v/v) was performed. Samples were dried completely in a vacuum centrifuge. Samples were digested by adding 2 µl of sequencing-grade modified trypsin (Promega) directly to the dried gel pieces, followed by overnight incubation at 37°C. Peptides were extracted with 10 µl of 0.1% (v/v) trifluoroacetic acid, 5% (v/v) acetonitrile in 10 mM NH_4_HCO_3_, aided by 10 min sonication, and C18 ZipTipps. Data acquisition was performed on a SYNAPT XS (Waters). Data analysis was performed with the ProteinLynx Global Server 3.0 software.

#### tRNA sequencing

To analyze the tRNA species bound to proteins, protein samples from two independent purifications were sent to the Epitranscriptomics & Sequencing Core Facility (Biopôle, Campus Brabois-Santé, Nancy, FR). The facility extracted the RNAs and performed next-generation sequencing according to the following protocol:

30 µl of each sample were combined with 1.05 µl of 1 M Tris–HCl (pH 8.0), 0.7 µl of 0.5 M EDTA (pH 8.0), and 3.5 µl of 10% (w/v) SDS [final concentrations: 30 mM Tris–HCl, 10 mM EDTA, 1% (w/v) SDS]. In addition, 2 µl of proteinase K (20 mg/ml) was added, and the mixture was incubated for 1 h at 37°C. The reaction was stopped by addition of 362.75 µl of 1 × AE buffer (50 mM NaOAc pH 5.2, 10 mM EDTA pH 8.0), 40 μl of 10% (w/v) SDS, and 440 μl of acid phenol. Following vortexing, the mixture was incubated for 4 min at 65°C and quickly cooled down on dry ice for 3 min. After centrifugation for 10 min at 13 200 rpm at room temperature, the aqueous (upper) phase was transferred to a new 1.5 ml of microcentrifuge tube. An equal volume of phenol–chloroform–isoamyl alcohol was added, the mixture vortexed and centrifuged for 10 min at 13 200 rpm and room temperature. The aqueous (upper) phase was again transferred in a new tube, treated with an equal volume of chloroform, and centrifuged. Finally, the aqueous (upper) phase was mixed in a new tube with 40 μl of AcONa (pH 5.2) and 1 ml of 96% (v/v) EtOH and if necessary 1 μl of glycoblue. After mixing, the sample was stored for at least 30 min at −80°C before centrifugation for 30 min at 4°C and 13 200 rpm. The pellet was washed with 500 μl of 80% (v/v) EtOH and the supernatant removed. The pelleted nucleic acids were air dried for 5 min at room temperature or 2 min at 37°C, before dissolving them in 10 µl of RNase-free water.

Then, the RNA was subjected to slight fragmentation by alkaline hydrolysis in 50 mM sodium-bicarbonate buffer at pH 9.2 and 96°C for 5 min. The reaction was stopped by ethanol precipitation using 3 M NaOAc, pH 5.2, and glycoblue. After centrifugation, the RNA pellet was washed with 80% (v/v) EtOH and resuspended in nuclease-free water. RNA fragments were end-repaired as previously described [34] and purified using RNeasy MinElute Cleanup kit according to the manufacturer’s recommendations, except that 675 µl of 96% (v/v) ethanol were used for RNA-binding step. Elution of purified RNA fragments was performed in 19 µl of nuclease-free water. RNA fragments were converted to library using the NEBNext^®^ Small RNA Library Prep Set for Illumina^®^ (NEB ref. E7330S, USA) following the manufacturer’s recommendations. DNA library was quantified using a fluorometer (Qubit 2.0 fluorometer, Invitrogen, USA) and qualified using a High Sensitivity DNA chip on Agilent Bioanalyzer 2100. Libraries were multiplexed and subjected for high-throughput sequencing on an Illumina Miseq with a 50 bp paired end read mode.

#### Thermal shift assay

Purified proteins were tested in triplicates for their thermostability under different conditions in a 96-well thin-wall PCR plate (ThermoFisher, Waltham, United States). Each well contained 1 µl of fluorescent dye indicator SYPRO Orange (Sigma–Aldrich, 5000× in dimethyl sulfoxide (DMSO), 1:40 diluted with H_2_O), 1 µl of 5 mg/ml protein and optionally 1 µl of 50 mM nucleotide [AMP, ATP, AMPCPP (adenosine-5′-[(α,β)-methyleno]triphosphate (Jena Bioscience, Germany)), and AMPNPP (adenosine-5′-[(α,β)-imido]triphosphate (Jena Bioscience, Germany))]. If not indicated otherwise, the total volume of 20 µl was adjusted with the corresponding buffer D (Supplementary Table S17). The plate was sealed, shortly centrifuged, and then heated in a Bio-Rad CFX96 Real-Time PCR Detection System from 4°C to 95°C in increments of 0.5°C/20 s, while the fluorescence was monitored. The results were analyzed by the Bio-Rad CFX Maestro software and melting temperatures were derived. The curves from three measurements for the same protein were averaged, normalized, and fitted with GraphPad Prism 5. Data points before the minimum and after the maximum of the fluorescence intensity were excluded from fitting [35].

#### *In vitro* activity assay with biotinylated tRNA stem loops

The activity of purified Tcd1–Tcd2 was tested *in vitro* with chemically synthesized t^6^A-modified stem loop tRNA^Arg(CCU)^ (Supplementary Table S3 and section on chemical synthesis later in text). As a control the nonmodified stem loop was used. Since Tcd1–Tcd2 enzyme preparations inherently contained tRNA from *E. coli* labeled with t^6^A and ct^6^A (see Supplementary Fig. S14), an essential step after the activity assay was to isolate the synthetic stem loops from the assay mixture and to check for any modification. To this end, the synthetic stem loops carried a biotin label at their 5′ end (Supplementary Table S3).

The assay mixture contained 16 µM t^6^A-modified or nonmodified stem loop, 1 mM ATP, and different concentrations of enzyme (0, 1, or 160 µM) in 200 mM Bis–Tris/HCl pH 6.5, 150 mM NaCl, 10% (v/v) glycerol, and 5 mM MgCl_2_. The total volume was adjusted with enzyme buffer to 50 µl, and the assay mixture was incubated for 16 h at 22°C.

Ten microliters of each reaction mixture was diluted with 190 µl of Milli-Q water to a final volume of 200 µl. An equal volume (200 µl) of phenol/chloroform/isoamyl alcohol (ROTI^®^Aqua-P/C/I, Roth) was added, followed by thorough mixing. After a 2-min incubation to allow phase separation, the upper aqueous phase was carefully collected. This extraction step was followed by two additional extractions with 200 µl of chloroform to ensure complete removal of organic contaminants. The aqueous phase was recovered after each extraction and subsequently subjected to bead-based purification.

Purification of biotinylated stem loops was performed using Dynabeads^™^ MyOne^™^ Streptavidin C1 (Thermo Scientific). Prior to sample binding, 50 µl of beads per reaction was prewashed (for all reactions combined) by washing three times with 1× B&W buffer (5 mM Tris–HCl pH 7.5, 0.5 mM EDTA, and 1 M NaCl) and once with 5× SSC buffer (prepared from a 20× stock solution: 3 M NaCl, 300 mM trisodium citrate, pH 7.0). After washing, the beads were resuspended in 50 µl per aliquot. Each sample from the pre-purified stem-loops was then incubated with a 50 µl of aliquot of washed beads at 25°C and 600 rpm for 30 min to ensure efficient binding. Following incubation, the beads were washed once with 1× SSC buffer and subsequently three times with 0.1× SSC buffer.

After the final wash, the beads were resuspended in 15 µl of Milli-Q water. On-bead digestion was performed by adding 15 µl of a digestion master mix to reach a final volume of 30 µl as described in section “Quantification of t^6^A and ct^6^A - digestion of RNA to nucleosides” above. The resulting digests were filtered and analyzed by LC–MS/QQQ as described above.

#### Software

All images illustrating protein structures and electron densities were created with PyMOL [36]. Figures were assembled using CorelDraw and BioRender. Sequence alignments were performed using T-Coffee, Escript [37], and Endscript [37]. Size-exclusion data or raw data from tRNA sequencing were analyzed using GraphPad Prism. Statistical analysis was performed with Excel.

#### Chemical synthesis of t^6^A phosphoramidite

**Scheme 1.**
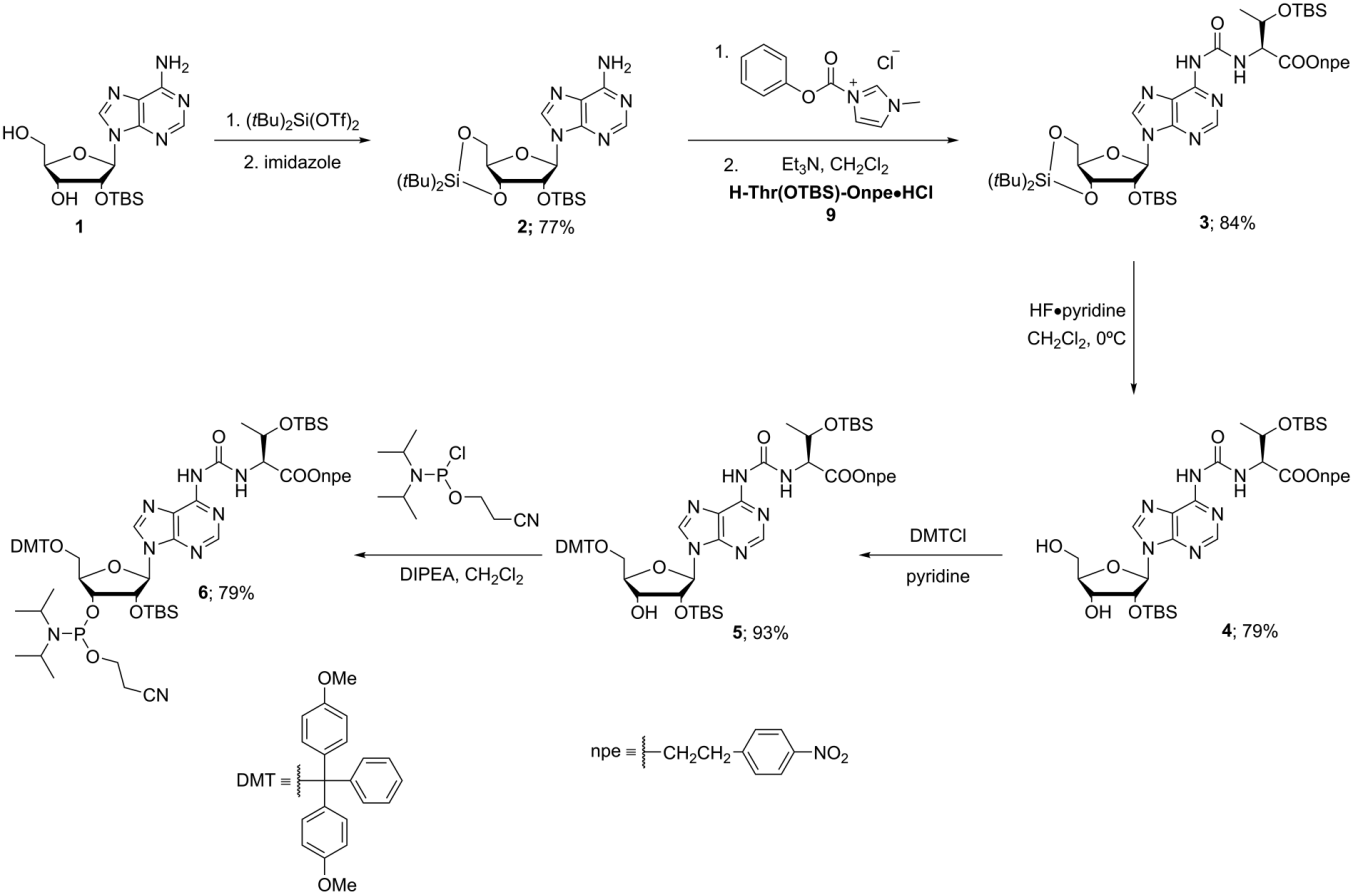
Chemical synthesis of the t^6^A phosphoramidite (compound 6).

The t^6^A phosphoramidite was synthesized following a previously reported procedure (Scheme 1) [14].

**General procedure for the synthesis of compound 2:** Adenosine **1** (1 equiv.) was suspended in dry *N, N*-dimethylformamide (DMF) and cooled to 0°C. Di-tert-butylsilyl (DTBS) ditriflate (1.2 eq) was added dropwise to the stirring mixture. The reaction was stirred at 0°C for 45 min. Imidazole (5 eq) was added and the reaction mixture was slowly warmed up to rt. TBS-Cl (1.2 eq) was added and the reaction was heated to 60°C overnight. The DMF was evaporated, then the crude was redissolved in EtOAC, washed with brine, and then extracted with EtOAc. The combined organic layers were dried over Na_2_SO_4_, filtered, and concentrated. The crude was purified by silica gel column chromatography affording the product as a white solid.

**2:** Yield: 77%. Rf = 0.33 (3:7 iHex/EtOAc). ^1^H NMR (500 MHz with cryoprobe, CDCl_3_, 298 K): *δ* (ppm) = 8.32 (s, 1H); 7.82 (s, 1H); 5.91 (s, 1H); 5.75 (s, 1H); 4.62 (d, J = 4.7 Hz, 1H); 4.54 (dd, J = 9.5 Hz, *J* = 4.8 Hz, 1H); 4.48 (dd, J = 9.3 Hz, *J* = 5.0 Hz, 1H); 4.24 - 4.19 (m, 1H); 4.03 (dd, J = 9.3 Hz, *J* = 1.1 Hz, 1H); 1.08 (s, 9H); 1.04 (s, 9H); 0.93 (s, 9H); 0.16 (s, 3H); 0.14 (s, 3H). ^13^C{^1^H} NMR (125 MHz with cryoprobe, CDCl_3_, 298 K): *δ* (ppm) = 155.6; 153.3; 149.4; 139.0; 120.5; 92.6; 75.9; 75.6; 74.8; 68.0; 27.6; 27.2; 26.0; 22.9; 20.5; 18.5; -4.2; -4.9. FTIR *v*_max_ (cm^−1^): 3149 (w); 2935 (w); 2860 (w); 1677 (m); 1602 (m); 1473 (m); 1167 (m); 1131 (m); 1063 (s); 1005 (s); 828 (vs); 652 (s). HRMS (ESI) *m*/*z*: [M + H]^+^ Calcd for C_24_H_44_O_4_N_5_Si_2_ 522.2926; Found 522.2926.

The corresponding NMR spectra are shown in Supplementary Figs S31 and S32.

**General procedure for the synthesis of compound 3:** Step 1: Compound **2** (1 equiv.) and 1-*N*-methyl-3-phenoxycarbonyl-imidazolium chloride (2 equiv.) were added to an oven-dried round-bottom flask and kept under high-vacuum for 15 min. After that, dry CH_2_Cl_2_ was added under nitrogen atmosphere and the reaction was stirred at r.t. for 5 h. Step 2: Onpe-protected amino acid **9•HCl** (2 equiv.) was added to an oven-dried round-bottom flask and suspended in dry CH_2_Cl_2_, followed by the addition of Et_3_N (2 equiv.). The suspension was added dropwise to the reaction mixture. The reaction was stirred at r.t. under nitrogen atmosphere for 2 days. After that, the reaction was quenched with aqueous saturated NaHCO_3_. The organic layer was separated, and the crude was further extracted with CH_2_Cl_2_. The combined organic layers were dried (Na_2_SO_4_), filtered, and concentrated. The crude was purified by silica gel column chromatography affording the product as a white foam.

**3:** Yield = 84%. Rf = 0.32 (2:3 iHex/EtOAc). ^1^H NMR (500 MHz with cryoprobe, CDCl_3_, 298 K): *δ* (ppm) = 10.03 (d, *J* = 9.1 Hz, 1H); 8.46 (s, 1H); 8.42 (s, 1H); 8.17 (s, 1H); 7.97–7.91 (m, 2H); 7.34–7.28 (m, 2H); 6.00 (s, 1H); 4.63 (d, *J* = 4.6 Hz, 1H); 4.58–4.46 (m, 3H); 4.41–4.34 (m, 2H); 4.29–4.22 (m, 1H); 4.05 (dd, *J* = 10.5 Hz, *J* = 9.2 Hz, 1H); 3.03 (t, *J* = 6.5 Hz, 2H); 1.25 (d, *J* = 6.3 Hz, 3H); 1.08 (s, 9H); 1.05 (s, 9H); 0.95 (s, 9H); 0.90 (s, 9H); 0.19 (s, 3H); 0.16 (s, 3H); 0.07 (s, 3H); -0.04 (s, 3H). ^13^C{^1^H} NMR (125 MHz with cryoprobe, CDCl_3_, 298 K): *δ* (ppm) = 171.0; 154.5; 151.2; 150.3; 149.8; 146.8; 145.6; 141.6; 129.8; 123.6; 121.2; 92.5; 75.9; 75.7; 74.9; 68.7; 67.9; 64.7; 59.7; 34.9; 27.6; 27.2; 26.0; 25.6; 22.9; 21.3; 20.5; 18.4; 17.9; −4.2; −4.9; −5.3. FTIR *v*_max_ (cm^−1^): 2896 (w); 1703 (m); 1611 (m); 1522 (s); 1345 (m); 1250 (m); 1058 (s); 999 (m); 828 (vs); 777 (s); 652 (m). HRMS (ESI) *m*/*z*: [M + H]^+^ Calcd. for C_43_H_71_O_10_N_7_Si_3_ 930.4642; Found 930.4650.

The corresponding NMR spectra are shown in Supplementary Figs S33 and S34.

**General procedure for the synthesis of compound 4:** Compound **3** (1 equiv.) was added to a plastic flask and dissolved in dry 9:1 CH_2_Cl_2_/pyridine. The solution was stirred at 0°C. Finally, HF•pyridine (from a commercial solution containing 70% HF and 30% pyridine) was added, and the reaction was stirred at 0°C for 2 h. After that, the reaction was quenched with aqueous saturated NaHCO_3_ and CH_2_Cl_2_ was added. The organic layer was separated and the crude was further extracted with CH_2_Cl_2_. The combined organic layers were dried (Na_2_SO_4_), filtered and concentrated. The crude was purified by silica gel column chromatography affording the product as a white foam.

**4:** Yield = 79%. Rf = 0.29 (96:4 CH_2_Cl_2_/IPA). ^1^H NMR (500 MHz with cryoprobe, CDCl_3_, 298 K): *δ* (ppm) = 9.88 (d, *J* = 9.1 Hz, 1H); 8.50 (s, 1H); 8.13 (s, 1H); 8.12 – 8.08 (m, 2H); 7.99 (s, 1H); 7.40 – 7.34 (m, 2H); 5.96 (dd, *J* = 12.1 Hz, *J* = 2.1 Hz, 1H); 5.82 (d, *J* = 7.3 Hz, 1H); 5.10 (dd, *J* = 7.3 Hz, *J* = 4.8 Hz, 1H); 4.58 (dd, *J* = 9.1 Hz, *J* = 1.6 Hz, 1H); 4.53 – 4.41 (m, 2H); 4.41 – 4.35 (m, 2H); 4.29 (dt, *J* = 11.0 Hz, *J* = 6.9 Hz, 1H); 3.97 (dt, *J* = 13.0 Hz, *J* = 2.0 Hz, 1H); 3.77 (td, *J* = 12.5 Hz, *J* = 1.5 Hz, 1H); 3.07 (t, *J* = 6.7 Hz, 2H); 2.84 (s, 1H); 1.24 (d, *J* = 6.2 Hz, 3H); 0.89 (s, 9H); 0.81 (s, 9H); 0.05 (s, 3H); -0.06 (s, 3H); -0.15 (s, 3H); -0.37 (s, 3H). ^13^C{^1^H} NMR (125 MHz with cryoprobe, CDCl_3_, 298 K): *δ* (ppm) = 170.9; 153.9; 151.1; 151.0; 149.3; 147.0; 145.5; 142.9; 130.0; 123.9; 122.3; 91.5; 87.7; 74.7; 72.9; 68.7; 65.1; 63.4; 59.7; 35.0; 25.6; 21.3; 18.0; -4.1; -5.2; -5.2; -5.3. FTIR *v*_max_ (cm^−1^): 2930 (w); 1697 (m); 1611 (m); 1520 (s); 1470 (m); 1345 (s); 1250 (m); 1094 (m); 836 (s); 777 (vs). HRMS (ESI) *m*/*z*: [M + H]^+^ Calcd for C_35_H_56_O_10_N_7_Si_2_ 790.3621; Found 790.3626.

The corresponding NMR spectra are shown in Supplementary Figs S35 and S36.

**General procedure for the synthesis of compound 5:** Compound **4** (1 equiv.) was dissolved in dry pyridine and stirred under nitrogen atmosphere at r.t. 4,4-Dimethoxytrityl chloride (1.5 equiv.) was added in two portions, and the reaction was stirred at r.t. overnight. After that, the crude was concentrated and purified by silica gel column chromatography (eluent containing 0.1% pyridine) affording the product as a white foam.

**5:** Yield = 93%. Rf = 0.35 (7:3 CH_2_Cl_2_/EtOAc). ^1^H NMR (500 MHz with cryoprobe, CDCl_3_, 298 K): *δ* (ppm) = 9.95 (d, *J* = 9.1 Hz, 1H); 8.75 (s, 1H); 8.50 (s, 1H); 8.37 (s, 1H); 8.02–7.97 (m, 2H); 7.52–7.49 (m, 2H); 7.48–7.44 (m, 2H); 7.34 (dq, *J* = 8.6 Hz, *J* = 3.2 Hz, 4H); 7.24 (dd, *J* = 8.3 Hz, *J* = 6.7 Hz, 2H); 7.20–7.15 (m, 1H); 6.85–6.77 (m, 4H); 6.13 (d, *J* = 4.4 Hz, 1H); 5.13 (t, *J* = 4.7 Hz, 1H); 4.53 (td, *J* = 5.5 Hz, *J* = 4.0 Hz, 2H); 4.48 (dd, *J* = 9.1 Hz, *J* = 1.7 Hz, 1H); 4.37 (qt, *J* = 11.1 Hz, *J* = 6.2 Hz, 2H); 4.26 (td, *J* = 4.7 Hz, *J* = 3.5 Hz, 1H); 3.96 (d, *J* = 5.8 Hz, 1H); 3.73 (s, 6H); 3.48–3.39 (m, 2H); 3.10 (t, *J* = 6.2 Hz, 2H); 1.25 (d, *J* = 6.3 Hz, 3H); 0.88 (s, 9H); 0.82 (s, 9H); 0.06 (s, 3H); 0.03 (s, 3H); −0.06 (s, 3H); −0.07 (s, 3H). ^13^C{^1^H} NMR (125 MHz with cryoprobe, CDCl_3_, 298 K): *δ* (ppm) = 171.6; 159.6; 154.7; 151.5; 151.4; 151.2; 147.6; 147.4; 146.1; 143.5; 136.7; 136.7; 131.1; 131.0; 131.0; 129.0; 128.6; 127.5; 124.1; 121.9; 113.8; 90.2; 87.1; 84.8; 76.3; 72.0; 69.6; 65.6; 64.4; 60.3; 55.5; 35.3; 26.1; 26.0; 21.5; 18.7; 18.4; −4.2; −4.6; −4.8; −5.2. FTIR *v*_max_ (cm^−1^): 2935 (w); 1701 (m); 1608 (m); 1509 (s); 1466 (m); 1345 (m); 1247 (s); 1033 (m); 828 (vs); 777 (s). HRMS (ESI) *m*/*z*: [M + Na]^+^ Calcd for C_56_H_73_O_12_N_7_Si_2_Na^+^ 1114.4753; Found 1114.4763.

The corresponding NMR spectra are shown in Supplementary Figs S37 and S38.

**General procedure for the synthesis of compound 6:** Compound **5** (1 equiv.) was added to an oven-dried round-bottom flask and dissolved in dry CH_2_Cl_2_. The solution was stirred under Argon atmosphere at 0°C. DIPEA (4 equiv.) was added dropwise. Finally, 2-cyanoethyl *N,N*-diisopropylchlorophosphoramidite (2.5 equiv.) was added dropwise. The reaction was stirred at r.t. for 3 h. After that, the reaction was stopped and diluted with CH_2_Cl_2_. The crude was washed with aqueous saturated NaHCO_3_ and the organic layer was separated. The crude was further extracted with CH_2_Cl_2_. The combined organic layers were dried (Na_2_SO_4_), filtered and concentrated. The crude was purified by silica gel column chromatography (eluent containing 0.1% pyridine). The products were isolated as a mixture of diastereoisomers as a white foam. Finally, the product was lyophilized from benzene.

**6:** Yield = 79%. Rf = 0.47 (1:1 iHex/EtOAc). ^31^P{^1^H} NMR (162 MHz with cryoprobe, acetone-*d*_6_, 298 K): *δ* (ppm) = 150.1; 148.5. HRMS (ESI) *m*/*z*: [M + Na]^+^ Calcd. for C_65_H_90_O_13_N_9_PSi_2_Na^+^ 1314.5831; Found 1314.5855.

The corresponding NMR spectrum is shown in Supplementary Fig. S39.

**Scheme 2.**
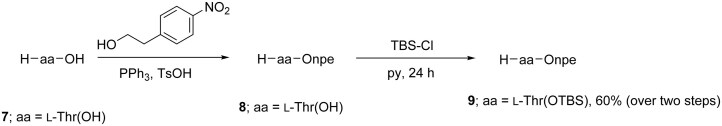
Chemical synthesis of compound 9.

The Onpe-protected amino acid **8** was synthesized following a previously reported procedure (Scheme 2) [14].

**General procedure for the synthesis of compound 9:** Compound **8** was dissolved in dry pyridine, then *tert*-butyldimethylsilyl chloride (TBDMS-Cl) (1.5 equiv.) and imidazole (3 equiv.) was added. After 10 min, another portion of TBDMS-Cl (1.5 equiv.) was added and the reaction was stirred at r.t. for 23 h. The reaction mixture was diluted with CH_2_Cl_2_ and washed with aq. sat. NaHCO_3_ and water. The organic layer was dried (MgSO_4_), filtered, and concentrated. The crude was purified by silica gel column chromatography affording the product as a brown oil.

**9:** Yield = 60% (over two steps). ^1^H NMR (500 MHz with cryoprobe, CDCl_3_, 298 K): *δ* (ppm) = 8.11 (d, *J* = 8.7 Hz, 2H); 7.35 (s, *J* = 8.7 Hz, 2H); 4.38 (dt, *J* = 11.0 Hz, *J* = 6.8 Hz, 1H); 4.22 (dt, *J* = 11.0 Hz, *J* = 6.8 Hz, 1H); 4.16 (qd, *J* = 6.3 Hz, *J* = 2.8 Hz, 1H); 3.21 (d, *J* = 2.8 Hz, 1H); 3.03 (t, *J* = 6.8 Hz, 2H); 1.17 (d, *J* = 6.3 Hz, 3H); 0.76 (s, 9H); −0.04 (s, 3H); −0.13 (s, 3H). ^13^C{^1^H} NMR (125 MHz with cryoprobe, CDCl_3_, 298 K): *δ* (ppm) = 174.3; 146.9; 145.6; 129.8; 123.8; 69.6; 64.4; 60.8; 34.9; 25.6; 20.9; 17.8; −4.3; −5.3. FTIR *v*_max_ (cm^−1^): 2930 (w); 2857 (w); 1739 (m); 1601 (w); 1519 (s); 1473 (w); 1345 (vs); 1252 (m); 1154 (m); 1075 (m); 969 (m); 835 (s); 775 (vs). HRMS (ESI) *m*/*z*: [M + H]^+^ Calcd for C_18_H_32_N_2_O_5_Si 383.1996; Found 383.1996.

The corresponding NMR spectra are shown in Supplementary Figs S40 and S41.

#### Chemical synthesis of stem loops

Phosphoramidites of canonical ribonucleosides (Bz-A-CE, Dmf-G-CE, Ac-C-CE, and U-CE) and the biotin phosphoramidite were purchased from Sigma–Aldrich. Oligonucleotides (Supplementary Table S3) were synthesized on a 1 μmol scale using High Load Glen UnySupport^™^ as solid support, using an RNA automated synthesizer (Applied Biosystems 394 DNA/RNA Synthesizer) with a standard phosphoramidite chemistry. Oligonucleotides were synthesized in DMT-OFF mode using DCA as a deblocking agent in CH_2_Cl_2_, BTT, or Activator 42^®^ as activator in MeCN, Ac_2_O as capping reagent in pyridine/THF, and I_2_ as oxidizer in pyridine/H_2_O.

Deprotection of npe group: For the deprotection of the *para*-nitrophenylethyl (npe) group in the oligonucleotide containing Thr-modified carbamoyl adenosine derivatives, the solid support beads were suspended in a 9:1 THF/DBU solution mixture (1 ml) and incubated at r.t. for 2 h [38]. After that, the supernatant was removed and the beads were washed with THF (3 × 1 ml).

Cleavage from beads: The solid support beads were suspended in a 1:1 aqueous solution mixture (0.6 ml) of 30% NH_4_OH and 40% MeNH_2_. The suspension was heated at 65°C (60 min for High Load Glen UnySupport^™^). Subsequently, the supernatant was collected and the beads were washed with water (2 × 0.3 ml). The combined aqueous solutions were concentrated under reduced pressure using a SpeedVac concentrator deprotection of TBS groups and precipitation of the synthesized oligonucleotide.

Deprotection of TBS groups and precipitation: The crude was dissolved in DMSO (100 μl) and triethylamine trihydrofluoride (125 μl) was added. The solution was heated at 65°C for 1.5 h. Finally, the oligonucleotide was precipitated by adding 3 M NaOAc in water (25 μl) and *n*-butanol (1 ml). The mixture was kept at −80°C for 2 h and centrifuged at 4°C for 1 h. The supernatant was removed and the white precipitate was lyophilized.

Purification of the synthesized oligonucleotide by HPLC and desalting: The crude was purified by semi-preparative HPLC (1260 Infinity II Manual Preparative LC System from Agilent equipped with a G7114A detector) using a reverse-phase (RP) VP 250/10 Nucleodur 100–5 C18ec column from Macherey-Nagel. Buffers: (i) 0.1 M AcOH/Et_3_N in H_2_O at pH 7 and (ii) 0.1 M AcOH/Et_3_N in 80% (v/v) MeCN in H_2_O. Gradient: 0%–25% of B in 45 min. Flow rate = 5 ml∙min^−1^. The purified oligonucleotide was analysed by RP-HPLC (1260 Infinity II LC System from Agilent equipped with a G7165A detector) using an EC 250/4 Nucleodur 100–3 C18ec from Macherey-Nagel. Gradient: 0%–30% or 0%–40% of B in 45 min. Flow rate = 1 ml∙min^−1^. Finally, the purified oligonucleotide was desalted using a C18 RP-cartridge from Waters.

Determination of the concentration and the mass of the synthesized oligonucleotide: The absorbance of the synthesized oligonucleotide in H_2_O solution was measured using an IMPLEN NanoPhotometer^®^ N60/N50 at 260 nm. The extinction coefficient of the single stranded oligonucleotides was calculated using the OligoAnalyzer Version 3.0 from Integrated DNA Technologies. For oligonucleotides incorporating noncanonical bases, the extinction coefficients were assumed to be identical to those containing only canonical counterparts. The synthesized oligonucleotide (2–3 μl) was desalted on a 0.025 μm VSWP filter (Millipore), co-crystallized in a 3-hydroxypicolinic acid matrix (HPA, 1 μl) and analyzed by MALDI–TOF mass spectrometry (negative mode).

## Results

### Bioinformatics analysis of yeast Tcd enzymes

Initial bioinformatics analysis of Tcd1 and Tcd2 from *S. cerevisiae* indicated for their N-terminal domain sequence similarities with the Rossmann fold of the adenylyltransferases ThiF and MoeB as well as ubiquitin-activating E1 enzymes [39, 40], while for the C-terminus a domain of unknown structure and function was predicted (Fig. 1C). A blast search identified a homologue with a similar domain architecture in *S. pombe* (encoded by the open reading frame SPAC1A6.10, Fig. 1C and Supplementary Fig. S1). A transmembrane domain, poorly conserved in sequence, was noted at the N-terminus [41] and excluded from all our constructs used for heterologous expression in *E. coli* (Supplementary Fig. S1). Although the domain architecture was initially indicative of a function related to the ubiquitin–proteasome system, work from Miyauchi *et al*. in 2013 [16] suggested that Tcd enzymes are required for installing ct^6^A in tRNAs.

### S. *pombe* Tcd copurifies with tRNAs and its C-terminal domain adopts a HNH fold

Similar to *E. coli, S. pombe* encodes a single Tcd enzyme (Fig. 1C). Initial efforts focused on the heterologous expression of a truncated version (coding for SpTcd^102-485^, Supplementary Fig. S1) as an N-terminal His_6_-SUMO fusion in *E. coli*. The protein of interest was isolated from the crude lysate by Ni affinity chromatography and after tag removal further purified by size-exclusion chromatography (Supplementary Fig. S2A). The retention volume at which the protein eluted indicated a much higher molecular weight than expected from the protein’s size. Considering the putative function in tRNA modification, we analyzed the purified SpTcd samples by native polyacrylamide electrophoresis (PAGE) and noted that the enzyme co-purified with a nucleic acid (Supplementary Fig. S2B). The sensitivity toward ribonuclease (RNase) implied that RNAs were bound to SpTcd (Supplementary Fig. S2C) and next-generation sequencing finally identified them as tRNAs. SpTcd co-purified predominantly with *E. coli* tRNA^Thr^ featuring the anticodon sequence UGU (Supplementary Fig. S2D). Since tRNA^Thr(UGU)^ is not the most abundant tRNA in *E. coli* [42, 43], it was selectively enriched during Tcd purification. This result agrees with the fact that the ct^6^A modification is present on tRNAs decoding ANN mRNA codons [17, 44] (Fig. 1A). Since the genetic code uses a number of ANN codons, multiple tRNAs are assumed to serve as substrates for Tcd enzymes and indeed the sequencing analysis revealed besides tRNA^Thr(UGU)^ also other tRNAs in the SpTcd protein sample, although at markedly lower frequency (Supplementary Fig. S2D). In an effort to solve the structure of SpTcd bound to tRNA, crystallization trials were performed, but the heterogeneity of the tRNA as well as the tendency of SpTcd to aggregate prevented crystallization.

**Figure 2.**
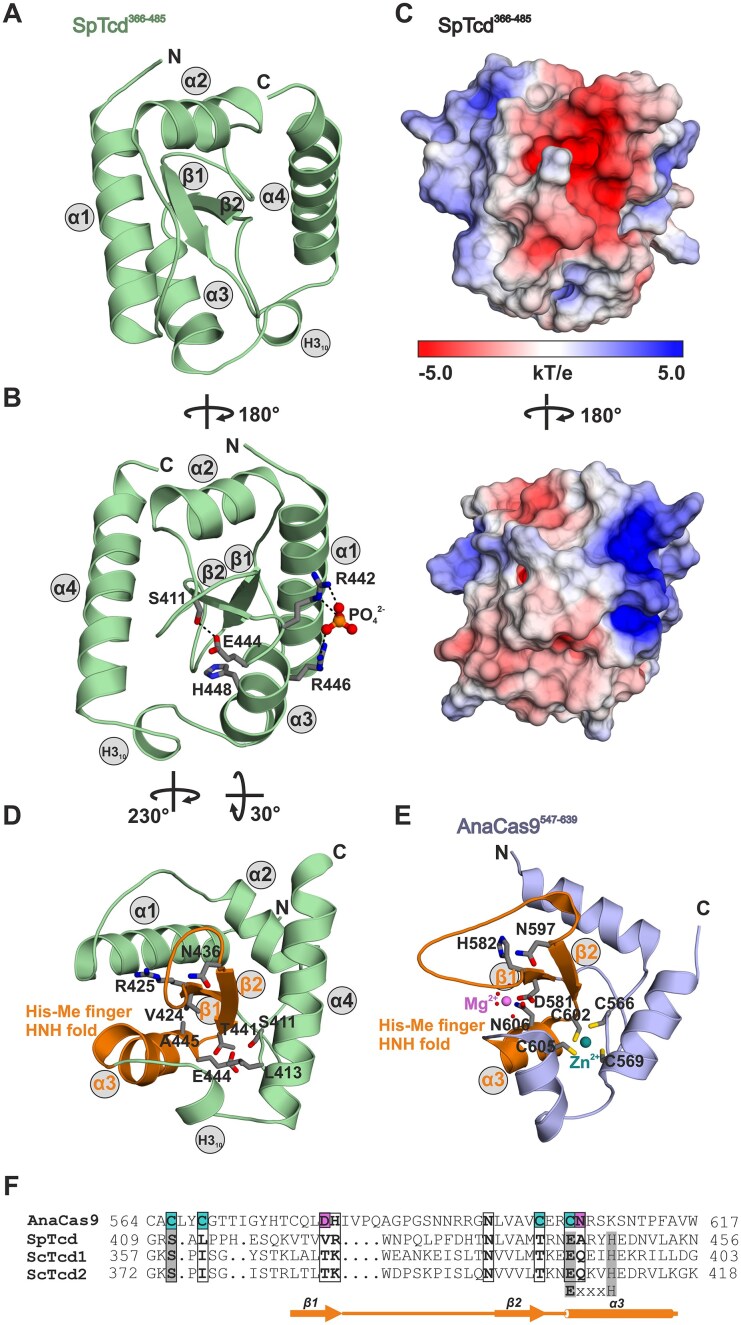
Crystal structure of *S. pombe* Tcd^366-485^. (**A**) 3D cartoon structure of SpTcd^366-485^ colored in green with the secondary structure elements as well as N- and C-termini labeled. (**B**) Ribbon illustration of SpTcd^366-485^ with key residues shown as sticks and labeled by the one-letter amino acid code. Ser411 and residues Glu444 as well as His448 of the ExxxH motif (see also Supplementary Fig. S1) do not form a catalytic triad. Arg442 and Arg446 coordinate a phosphate ion that may mimic a nucleic acid phosphate moiety. (**C**) Electrostatic surface illustrations [−5.0 *kT*/*e* (red) to 5.0 *kT*/*e* (blue)] of SpTcd^366-485^ according to the two orientations shown in panels (A) and (B). (**D**) Cartoon image of SpTcd^366-485^ with the His-Me finger/HNH fold highlighted in orange. Residues shown as sticks correspond to key amino acid positions for catalytic activity and Mg^2+^ or Zn^2+^ binding in AnaCas9 (panel E) but their identity is not conserved in Tcd enzymes (see also panel F). (**E**) Ribbon illustration of the NHN domain (residues 547–639) of the Cas9 endonuclease from *Actinomyces naeslundii* (AnaCas9) (PDB code: 4OGC [46]). AnaCas9 has been identified in a Dali search [45] as the closest structural relative of SpTcd^366-485^ (Supplementary Table S2 and Supplementary Fig. S5A). Structurally and catalytically relevant residues are depicted as sticks and labeled. While SpTcd^366-485^ and AnaCas9 share a conserved, structurally relevant Asn residue (Asn436 in SpTcd^366-485^ and Asn597 in AnaCas9) at the N-terminus of β-strand 2 of the His-Me finger [50], none of the other key residues of AnaCas9 are preserved in SpTcd^366-485^ (see panel D). The general base of AnaCas9, His528 [46], is replaced by Arg425 in SpTcd^366-485^ and all amino acids involved in coordinating the catalytically relevant Mg^2+^ ion (Asp581 and Asn606) are missing in SpTcd^366-485^ (panel D). SpTcd^366-485^ is therefore most likely catalytically inactive. Furthermore, the four cysteines building the noncatalytic zinc binding site of AnaCas9 are absent from SpTcd^366-485^. (**F**) Sequence comparison of yeast Tcd enzymes and AnaCas9. Residues adopting the NHN fold are shown. Structurally or catalytically important amino acids and residues relevant for Mg^2+^ (cyan) or Zn^2+^ (pink) binding in AnaCas9 are boxed and printed bold. The majority of these residues is not conserved in yeast Tcds. Key residues of the HNH fold of yeast Tcds are highlighted against a gray background. These include the Glu and His residues as part of the ExxxH motif and a conserved Ser. A schematic illustration of secondary structure elements for the NHN domain of SpTcd is shown below the sequence alignment. Please note that the length of secondary structure elements (in particular helix α3) in AnaCas9 slightly deviates from this annotation.

As the C-terminal domain of SpTcd (residues 366–485, Fig. 1C and Supplementary Fig. S1) was predicted to potentially be an enzyme itself (personal communication of Prof. Kay Hofmann, University of Cologne, Germany), we aimed for experimental structural data. Expression and purification by Ni affinity and size-exclusion chromatography was straight forward. The protein eluted as a monomer (Supplementary Fig. S3A) and did not co-purify with a nucleic acid (Supplementary Fig. S3B), indicating that SpTcd^366-485^ on its own is unable to mediate dimerization (as anticipated for SpTcd) and tRNA binding. Notably, SpTcd^366-485^ was about 3–5°C more stable than SpTcd^102-485^ that in addition included the N-terminal ThiF domain and had co-purified *E. coli* tRNA bound (Supplementary Fig. S4A and B). SpTcd^366-485^ was crystallized (Supplementary Fig. S3C) and the structure was solved by *ab initio* phasing (Supplementary Table S1). SpTcd^366-485^ folds into a twisted two-stranded β-sheet surrounded by α-helices (α–α–β–β–α–α) (Fig. 2A–D). A Dali search [45] highlighted this fold as rather exceptional with only few related structures and rather low *Z*-scores below the cutoff for a strong match (Supplementary Table S2). To our surprise, the most related known proteins are Histidine–Asparagine–Histidine (HNH) domains of CRISPR–Cas9 endonucleases [46], prevalent restriction endonucleases like McrA [47] or PacI [48], and Cap5 endonuclease from the cyclic oligonucleotide-based antiphage signaling system, which is related to the cGAS–STING pathway in humans [49] (Supplementary Table S2). Structural comparison of SpTcd^366-485^ with its closest relative, the Cas9 nuclease from *Actinomyces naeslundii* (AnaCas9) [46], revealed that while the His-Me finger/HNH fold is conserved (Supplementary Fig. S5A) the general base, His582, and key residues for binding of the catalytically relevant Mg^2+^ ion [50] are missing (Fig. 2D–F). Similar results were obtained when comparing SpTcd^366-485^ with the restriction enzymes PacI (Supplementary Fig. S5B) and McrA. PacI encodes a catalytically relevant Arg93 that occupies the same position as Arg425 in SpTcd^366-485^, but SpTcd^366-485^ lacks the other catalytic residues that PacI has, e.g. Tyr100 and His42 (Fig. 2D and Supplementary Fig. S5C) [48]. Based on the PacI:DNA co-crystal structure (PDB code: 3LDY [48], Supplementary Fig. S5C), we modeled a SpTcd^366-485^–DNA complex by superposition (Supplementary Fig. S5D). Assuming a certain conformational flexibility of the protein, nucleic acid binding to SpTcd^366-485^ may be possible. Yet, SpTcd^366-485^ features significantly fewer basic patches compared to PacI to mediate nucleic acid interactions (Supplementary Fig. S5E and F, and Fig. 2C) and an AlphaFold 3 prediction [51] of SpTcd^366-485^ and the DNA duplex used for PacI crystallization did not yield a complex model. We, therefore, conclude that SpTcd^366-485^ alone is unlikely to mediate strong nucleic acid interactions. However, in support of a potential minor role in nucleic acid binding, SpTcd^366-485^ was crystallized with a phosphate ion that localizes close to the region that in PacI mediates DNA binding (Fig. 2B, and Supplementary Fig. S5C and D).

Before any structural data on SpTcd^366-485^ have become available, bioinformatic analyses suggested a hydrolytic activity for the SpTcd^366-485^ domain. A conserved serine residue (Ser411) and a ExxxH motif, comprising Glu444 and His448, were speculated to engage in a Ser-His-Glu catalytic triad (personal communication of Kay Hofmann (University of Cologne, Germany), Fig. 2F and Supplementary Fig. S1). In the X-ray structure however, Glu444 and His448 are not in hydrogen bonding distance to each other (Fig. 2B). In contrast, Ser411 tightly hydrogen bonds to Glu444 (2.5 Å, Fig. 2B), but there is no putative substrate binding pocket close by. As such this interaction appears to rather stabilize the fold as a compensation for the lack of the zinc finger (CXXC)_2_ that is often part of HNH endonucleases [52] (Fig. 2E) but absent from SpTcd^366-485^ (Fig. 2B and D). Altogether, considering that SpTcd^366-485^ is lacking the catalytic components for nuclease activity, we assume that it is most likely catalytically inactive.

### Biochemical and structural studies on *S. cerevisiae* Tcds

#### *S. cerevisiae* Tcds localize to the outer mitochondrial membrane

**Figure 3.**
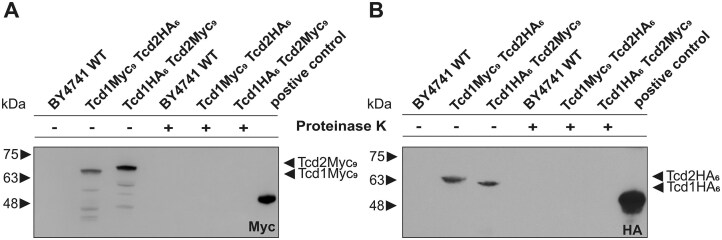
Intracellular localization of Tcd1 and Tcd2. Mitochondria were isolated from three different *S. cerevisiae* strains: Wild-type BY4741, Tcd1HA_6_-Tcd2Myc_9_ tagged BY4741, and Tcd1Myc_9_-Tcd2HA_6_ tagged BY4741. Half the isolated mitochondria extract was digested for 30 min with 200 µl/ml proteinase K. All lysates were separated by SDS–PAGE (12%) and analyzed by western blotting using monoclonal antibodies against Myc (**A**) and HA (**B**) tags (Supplementary Table S14). Molecular weights are indicated on the left, and the corresponding proteins on the right of each blot. Controls for the successful isolation of mitochondria are provided in Supplementary Fig. S6.

In contrast to *S. pombe, S. cerevisiae* encodes two Tcd enzymes: Tcd1 and Tcd2 (Fig. 1C). Proteomics analyses localized both proteins to the outer mitochondrial membrane [41]. To verify the subcellular localization and probe the orientation of Tcd1 and Tcd2 in the membrane, we fused the chromosomally encoded *TCD1* and *TCD2* genes with the coding sequence of either a nona-c-myc (Myc_9_) or a hexa-hemagglutinin (HA_6_) tag. The resulting fusion proteins had a native N-terminus (required for correct membrane insertion) and the corresponding C-terminal tag that could be detected by western blotting. From both WT and chromosomally tagged strains (producing either Tcd1-Myc_9_ and Tcd2-HA_6_ or Tcd1-HA_6_ and Tcd2-Myc_9_) mitochondria were isolated (Supplementary Fig. S6) and split into two samples. After one half was treated with proteinase K, all samples were blotted and probed with either an anti-HA_6_ or an anti-Myc_9_ antibody. The proteinase K treated samples did not show any signal for the Myc_9_- and HA_6_-tags, while the non-treated samples did (Fig. 3), indicating that the C-terminus of Tcd1 and Tcd2 points into solution and hence is accessible to proteolytic digestion. In conclusion, we verified that Tcd1 and Tcd2 localize to the outer mitochondrial membrane and face the cytosol, which perfectly matches their described function to modify cytosolic tRNAs [16].

#### Separate isolation and characterization of Tcd1 and Tcd2

Although it was previously proposed that both Tcd1 and Tcd2 are required for the formation of ct^6^A [16], it remained unclear whether there is any functional or physical interaction between them. We therefore separately produced Tcd1 and Tcd2 without their N-terminal transmembrane segment (Supplementary Fig. S1) as N-terminal, protease-cleavable His_6_ fusions in *E. coli*.

First attempts to produce Tcd2^62-447^ with only a N-terminal His_6_-tag followed by a tobacco etch virus (TEV) protease cleavage site did not yield soluble protein. To enhance the solubility of Tcd2, we switched to a His_6_-SUMO tag and used a codon-optimized synthetic gene as well as SolubBL21 *E. coli* cells for expression. This way, we obtained a soluble protein fraction (Supplementary Fig. S7). However, attempts to remove the His_6_-SUMO-tag resulted in irreversible precipitation and aggregation of Tcd2 under the chosen conditions, preventing isolation of the protein for follow-up studies.

**Figure 4.**
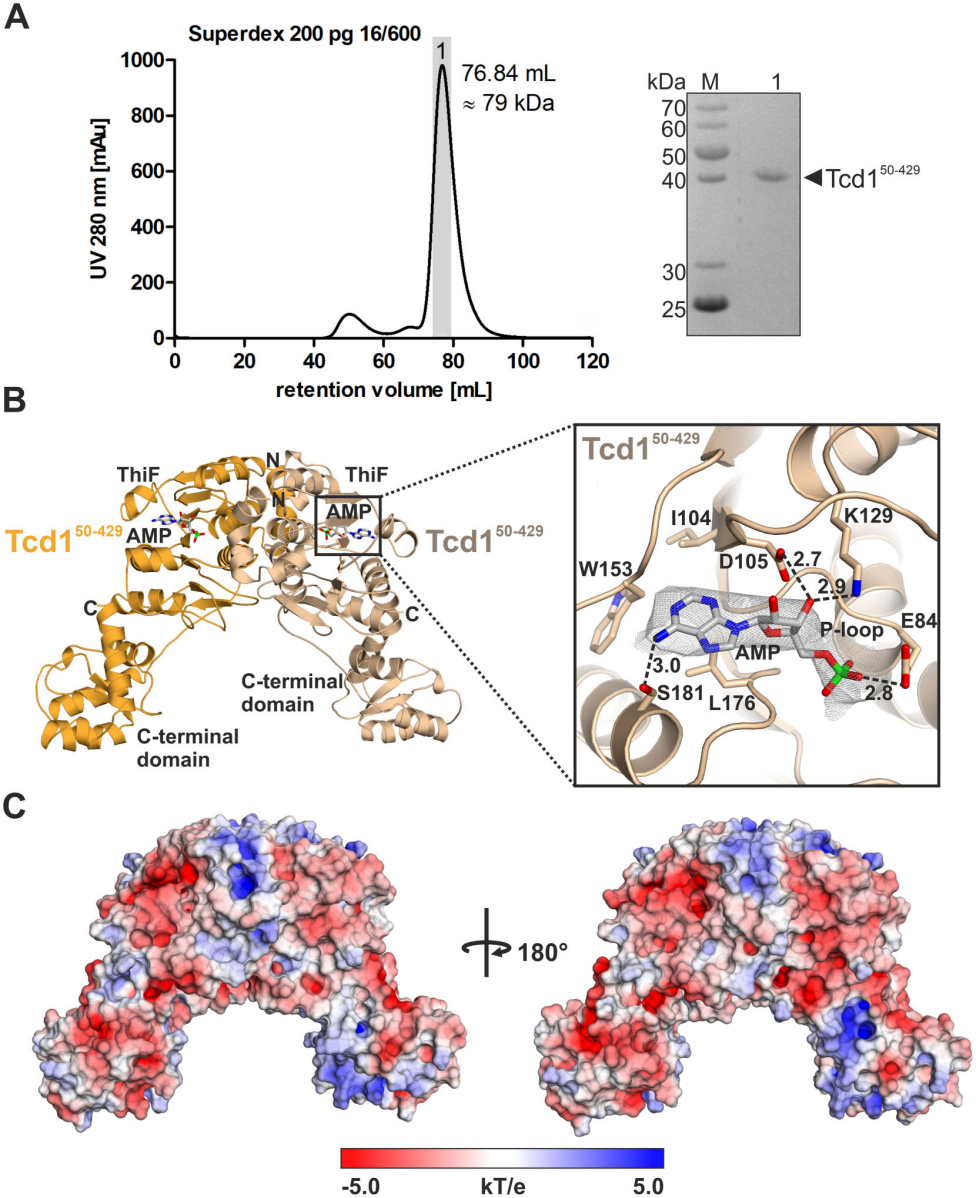
Crystal structure of the *S. cerevisiae* Tcd1^50-429^ homodimer with bound AMP. (**A**) Size-exclusion chromatography profile of the Tcd1^50-429^ homodimer with the apparent relative molecular mass (*M*_r_) calculated from the calibration curve in Supplementary Fig. S28B. Peak fractions were pooled and analyzed by SDS–PAGE (12%). The band at ∼40 kDa fits the molecular weight of a Tcd1^50-429^ monomer (43.5 kDa). (**B**) Left: Ribbon representation of the Tcd1^50-429^ homodimer with bound AMP as solved by X-ray crystallography. The ThiF and C-terminal domains as well as the N- and C-termini are labeled. AMP is shown as multicolored stick model. Right: Zoom-in at the cofactor binding site with AMP and the coordinating residues shown as sticks. Hydrogen bonds are indicated by black dotted lines and distances are given in Å. The mF_O_–DF_C_ Polder omit map [54] (gray mesh) for AMP is shown at a contour level of 4 σ (calculated from PDB code: 9TZH). Residues 81–87 form the P-loop typical of ATP binding proteins (GAGEVGS sequence) but with an unusual Glu84 residue (see also Supplementary Fig. S1). (**C**) Electrostatic surface illustrations of the Tcd1 homodimer with colors ranging from −5.0 *kT/e* (red) to 5.0 *kT/e* (blue). Tcd1 lacks a characteristic positively charged patch that could serve the binding of nucleic acids.

In contrast, the production and purification of Tcd1^50-429^ as a His_6_-TEV fusion protein was successful. After tag removal, size exclusion chromatography indicated a homodimeric assembly (Fig. 4A), similar to TcdA from *E. coli* [19, 20]. Surprisingly, native PAGE showed that Tcd1 did not co-purify with any tRNA (Supplementary Fig. S8A) and also *in vitro* transcribed tRNAs with a NNU anticodon sequence did not bind to Tcd1 (Supplementary Fig. S8C). To obtain structural insights, we performed crystallization trials with Tcd1 and adenosine monophosphate (AMP) and yielded hardly reproducible and highly fragile, needle-like crystals (Supplementary Fig. S8B) with a solvent content of 80%. Nonetheless, the X-ray structure of Tcd1 was solved (Supplementary Table S1) and revealed a V-shaped homodimer (Fig. 4B). In agreement with the structural data on *E. coli* TcdA [19, 20], exclusively the ThiF domain of Tcd1 mediates homodimerization (Fig. 4B). The interface area of about 2600 Å^2^ is classified as stable by PISA analysis [53] and formed by mostly hydrophobic contacts. At the single subunit level, the ThiF and C-terminal domains are structurally related to *E. coli* TcdA and SpTcd^366-485^, respectively, with r.m.s.d. values < 2 Å (Supplementary Fig. S9A and B). The electron density map revealed an AMP bound to the ThiF domain (Fig. 4B and Supplementary Fig. S9C). Superposition of Tcd1 and ATP-bound TcdA (PDB code: 4D79 [20]) implied steric clashes of Glu84 with ATP (Supplementary Fig. S9D). Since the Walker motif of known Tcd enzymes encodes a Gly at this position (Supplementary Fig. S1), we wondered whether Glu84 might impede ATP binding to Tcd1.

We therefore created a Tcd1–E84G mutant and analyzed its thermal stability in the absence and presence of nucleotides like AMP, ATP, AMPCPP, and AMPNPP and in comparison to WT Tcd1 (Supplementary Fig. S10). The data indicate that the Tcd1–E84G mutant is better stabilized by ATP, AMPCPP, and AMPNPP than WT Tcd1 (Supplementary Fig. S10G), hence indirectly confirming that Glu84 in Tcd1 hinders binding of ATP. In agreement, Tcd1 also fails to bind tRNAs *in vitro* (Supplementary Fig. S8C) and lacks prominent basic surface charges that could mediate strong nucleic acid binding (Fig. 4C). Altogether, these results question a catalytic function of Tcd1 in ct^6^A formation.

#### Tcd1 and Tcd2 are required for tRNA binding in *E. coli*

**Figure 5.**
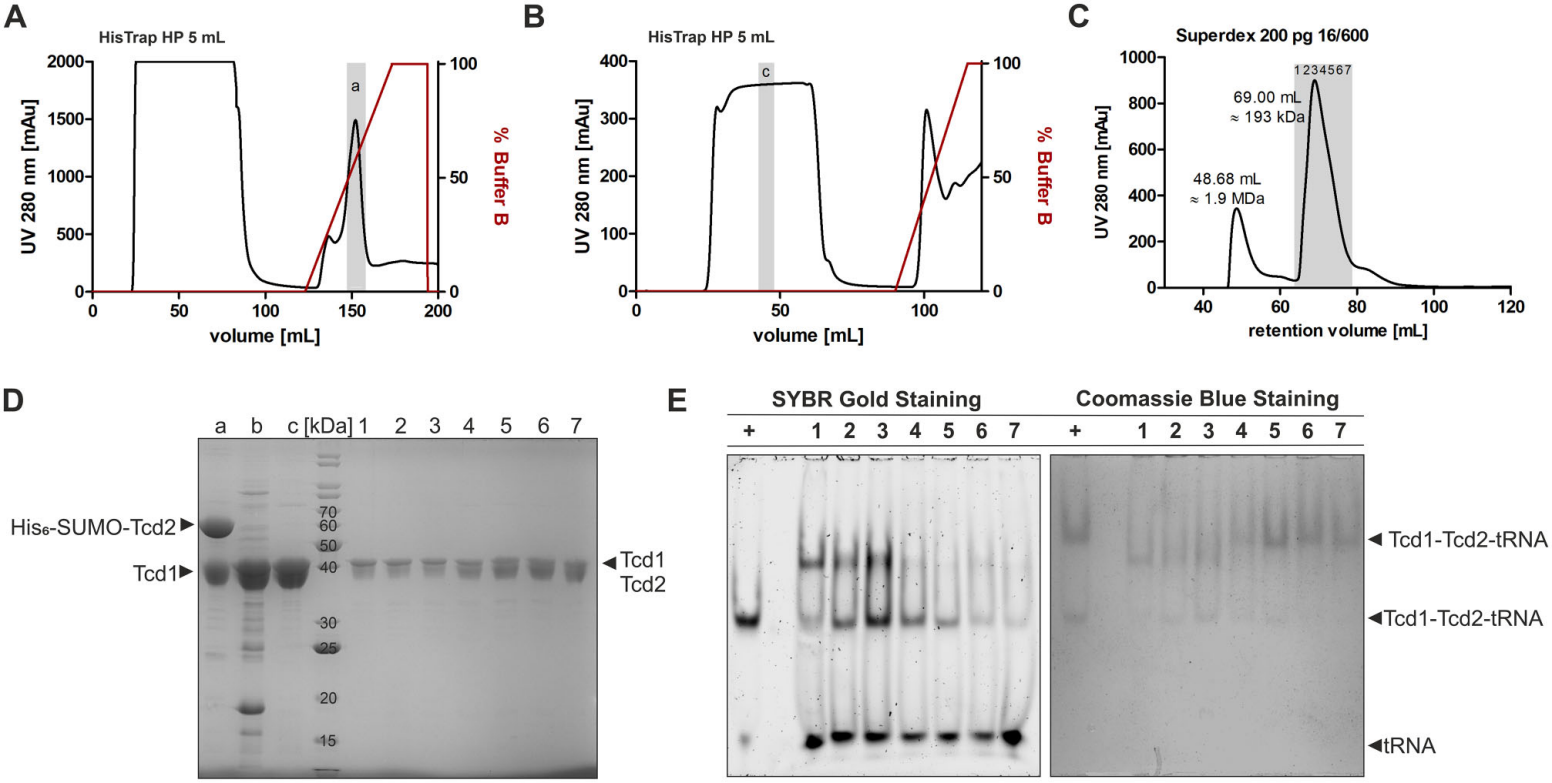
Protein purification of the *S. cerevisiae* Tcd1^50-429^-Tcd2^62-447^-tRNA complex with only Tcd2 being tagged. (**A–C**) Ni affinity (A and B) and size-exclusion chromatography (C) profiles for the purification of the Tcd1–Tcd2–tRNA complex. Samples analyzed by SDS- and native PAGE (see panels D and E) are indicated by gray bars and labeled. Cleavage of the His_6_-SUMO-tag from Tcd2 was performed between the two Ni affinity chromatography steps. *M*_r_ values indicated in the size-exclusion profile were calculated from the calibration curve in Supplementary Fig. S28B. (**D**) Protein samples taken during purification were analyzed by SDS–PAGE (12%). Lane a (peak fraction from first Ni affinity chromatography) illustrates two distinct bands corresponding to His_6_-SUMO-Tcd2 (56.8 kDa) and Tcd1 (43.5 kDa). Lanes b (sample after dialysis and tag cleavage) and c (flow through of second Ni affinity chromatography, i.e. tag-free protein) show two overlapping bands at 43 kDa corresponding to tag-free Tcd2 (43.3 kDa) and Tcd1 (43.5 kDa). Lanes 1–7 represent samples from size-exclusion chromatography after tag cleavage according to panel (C). (**E**) Native PAGE of peak fractions 1–7 from panel (C) after tag cleavage; Tcd1–Tcd2–tRNA complex obtained by a different purification strategy (see Supplementary Fig. S13) was used as a positive control (+). The staining with SYBR Gold and Coomassie Blue reveals bands at the same height, suggesting tRNA binding.

Considering the insolubility of Tcd2 *in vitro* (Supplementary Fig. S7) as well as the need of both Tcd1 and Tcd2 for ct^6^A formation *in vivo* [16], we speculated that both proteins might form a heterodimer. To test this hypothesis, cell pellets of *E. coli* having individually produced His_6_-TEV-Tcd1^50-429^ and His_6_-SUMO-Tcd2^62-447^ were mixed and the protein was purified (Supplementary Fig. S11A–C). Notably, size exclusion chromatography indicated a double peak (Supplementary Fig. S11C versus Fig. 4A), the reason for which could be traced back to co-purification of ribonucleic acids (Supplementary Fig. S11D and E) and in particular to tRNA^Thr(UGU)^ (Supplementary Fig. S11F). This result suggested that both Tcd1 and Tcd2 are required for tRNA binding but did not prove the formation of a heterocomplex. We therefore additionally established a co-expression system for the two proteins in *E. coli* in which only Tcd2 was His_6_-SUMO-tagged. Notably, Ni affinity chromatography captured not only His_6_-SUMO-tagged Tcd2 but also untagged Tcd1 and the two proteins co-purified with a nucleic acid (Fig. 5 and Supplementary Fig. S12), ultimately confirming the Tcd1–Tcd2 heterocomplex.

In parallel, we optimized the purification conditions (Supplementary Fig. S4C–F) and set-up a co-expression system with tRNA^Thr(UGU)^ from *S. cerevisiae* (Supplementary Fig. S13). After isolation of the co-expressed Tcd1–Tcd2–tRNA complex (Supplementary Figs S13A–D), the tRNAs were sequenced and although tRNA^Thr(UGU)^ from *S. cerevisiae* was the main species (43%, Supplementary Fig. S13E), >20% were still tRNA^Thr(UGU)^ from *E. coli*. Either the Tcd1–Tcd2 complex has higher affinity for *E. coli* tRNA^Thr(UGU)^ than for the yeast variant or the levels of *S. cerevisiae* tRNA^Thr(UGU)^—despite constitutive expression—were too low to completely displace the *E. coli* counterpart. In conclusion, our efforts to co-express yeast tRNA^Thr(UGU)^ rather increased than improved the heterogeneity of the sample.

To investigate if the co-purified tRNAs are substrates (carrying t^6^A) or products (carrying ct^6^A) of the Tcd catalyzed reaction or a mixture of both, we extracted the tRNAs from purified protein samples, digested them, and analyzed their composition by HPLC-MS. This way, we found that t^6^A and ct^6^A are each present on 1 out of 3.2 tRNAs (Supplementary Fig. S14), implying that Tcd enzymes co-purify with (non-)substrate and product tRNAs.

#### Analysis of the Tcd1–Tcd2–tRNA complex purified from *S. cerevisiae*

As it is astonishing that enzymes form long-lasting complexes with substrates and products, we wondered whether heterologous expression might be the reason and overproduced Tcd1 and Tcd2 in its native host, *S. cerevisiae*. To prevent SUMO-tag removal from Tcd2 by ubiquitin-like-protease 1 (Ulp1), a SUMO^star^ variant (LifeSensors Inc.) was used, but purification attempts merely yielded Tcd1. Only overproduction of both Tcd1^50-429^ and Tcd2^62-447^ as N-terminal His_6_-TEV fusions allowed for complex isolation (Supplementary Fig. S15A) and native PAGE analysis indicated co-purification of tRNAs (Supplementary Fig. S15B). Next-generation sequencing revealed the presence of a number of different tRNAs, all of which decode ANN codons (Supplementary Fig. S15C). The two most prominent tRNAs were tRNA^Asn(GUU)^ and tRNA^Arg(CCU)^, each present at about 30%. These data indicate that it is an intrinsic feature of yeast Tcd enzymes to co-purify with tRNAs and that the preferred tRNA and the distribution of bound tRNAs depends on the tRNA sequences available in a given expression host as well as the t^6^A modification efficiency.

To further explore this issue of tRNA co-purification, we purified *E. coli* TcdA under high-salt conditions as described by López-Estepa *et al*. (2015) and low-salt conditions as established for the Tcd1–Tcd2 complex from *S. cerevisiae*. We hereby noted that TcdA from *E. coli* also co-purifies with tRNAs under low salt conditions, while high salt conditions yielded the protein alone (Supplementary Fig. S16). Therefore, additional washing steps with high salt buffers or sodium bromide and anion exchange chromatography were included in the Tcd1–Tcd2 purification process. However, none of the tested purification conditions led to a protein preparation definitely containing both Tcd1 and Tcd2 without tRNA. These results let us conclude that co-purification of tRNAs is an inherent property of Tcd enzymes.

#### Attempts to map the tRNA binding site

**Figure 6.**
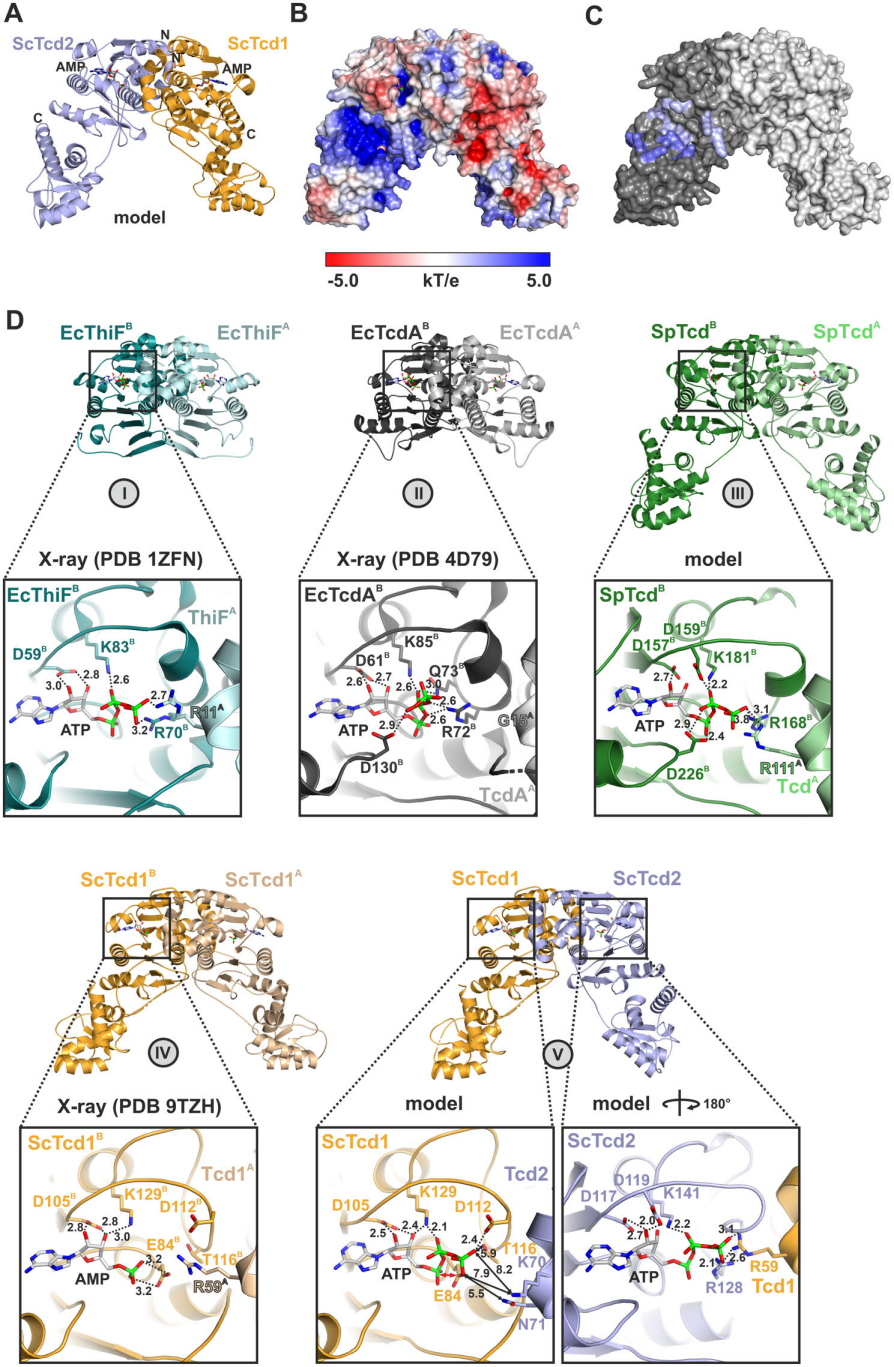
Structural model for the Tcd1–Tcd2 heterocomplex and related enzymes. N-terminal transmembrane segments for yeast enzymes are not displayed. X-ray structures are shown where available, otherwise AlphaFold models, as indicated. (**A**) Cartoon model of the Tcd1–Tcd2 heterocomplex, generated by superposition of the AlphaFold 2 prediction of Tcd2 (light blue) onto the X-ray structure of the Tcd1 homodimer (orange). (**B**) Electrostatic surface illustration of the Tcd1–Tcd2 heterodimer according to panel A with basic patches highlighted in blue and acidic ones in red. (**C**) Surface illustration of the Tcd1–Tcd2 heterodimer according to panel A with Tcd2 in dark gray and Tcd1 in light gray. Residues mutated in the ThiF domain of Tcd2 are colored light blue (residues 237–293, 8E-construct), and those in the C-terminal domain dark blue (residues 371–438, 6E construct). (**D**) Structural comparison of various Tcd enzymes (II–V) with the related *E. coli* protein ThiF (I, teal) from thiamine biosynthesis [39]. The nucleotides in the models were placed by superposition with the *E. coli* TcdA:ATP structure (PDB code: 4D79) [20]. Distances are given in Å. ThiF (I) is homologous to the N-terminal domain of TcdA (II, gray) from *E. coli*, Tcd (III, green) from *S. pombe* as well as Tcd1 (IV, orange) and Tcd1–Tcd2 (V, orange-light blue) from *S. cerevisiae*. The proteins form dimers with two active sites. For the homodimers ThiF, TcdA, SpTcd, and ScTcd1 only one of two identical active sites is depicted. ThiF (I) and SpTcd (III) use an arginine finger (Arg11/Arg111) from the neighboring subunit. ScTcd1 (IV) harbors the arginine finger (Arg59) but misses the other ATP coordinating arginine residue that is present in ThiF (Arg70), TcdA (Arg72), and SpTcd (Arg168). In the Tcd1–Tcd2 heterocomplex (V) two distinct active sites exist. Tcd2 does not contribute an arginine finger to the Tcd1 active site and Lys70 as well as Asn71 are located too far from the nucleotide to stimulate ATP hydrolysis in Tcd1 (double arrows). By contrast, Tcd1 complements the Tcd2 active site with Arg59.

Based on the X-ray structure of Tcd1 and the AlphaFold 2 [29] prediction of Tcd2 (note: AlphaFold 3 for the prediction of complexes was not yet available), the Tcd1–Tcd2 heterocomplex was modeled by superposition (Fig. 6A). The surface charge distribution indicated a prominent basic patch on the outer rim between the ThiF and C-terminal domains of Tcd2 only, suggesting that most likely Tcd2 alone is responsible for tRNA binding (Fig. 6B). To experimentally support this observation and to map the tRNA binding site, we selected 14 lysine or arginine residues in the putative tRNA binding pocket of Tcd2 for mutagenesis (Supplementary Fig. S1 and Fig. 6C). From a 14E-Tcd2-construct having all 14 selected basic residues replaced by glutamates, we created an 8E-construct, containing 8 mutations in the ThiF domain (between residues 237–293), and a 6E-construct, featuring 6 mutations in the C-terminal domain (between residues 371 and 438). After co-production of the different mutant His_6_-SUMO-Tcd2^62-447^ variants with His_6_-TEV-Tcd1^50-429^, protein purification was attempted. Notably, only for the His_6_-SUMO-Tcd2^62-447^-8E-construct we were able to isolate tRNA free Tcd1–Tcd2 complex (Supplementary Fig. S17), implying that the mutant residues in the ThiF domain contribute to tRNA recognition. Support for this experimental data was recently obtained from a computational AlphaFold 3 [51] model of the Tcd1–Tcd2–tRNA complex (Supplementary Fig. S18). In the following, we gradually mutated the eight point mutations in Tcd2–8E back to WT to identify which of the eight residues are essential for tRNA binding. Numerous constructs were expressed and purified (for details see Supplementary Table S4), but no definitive conclusions about the hierarchy of residues required for tRNA binding could be derived. Most likely these basic amino acid side chains have redundant functions in tRNA recognition, leading to additive and compensation effects.

#### Activity assays reveal Tcd2 residues crucial for ct^6^A formation

Next, we tested the activity of the heterocomplex towards chemically synthesized tRNA^Arg(CCU)^ stem loops carrying the t^6^A modification (Supplementary Table S3) by HPLC-MS, but no activity was detectable (Supplementary Fig. S19), suggesting that the small stem loops are likely not accepted as substrates. To additionally rule out an impact of other post-translational tRNA modifications, we chose an *in vivo* approach. First, a *S. cerevisiae* strain lacking both Tcd1 and Tcd2 (*Δtcd1Δtcd2*) was created from the corresponding single knockout strains. Growth tests of WT, single and double knockout strains revealed slight differences in fitness under glycerol conditions (Supplementary Fig. S20). This observation agrees with the fact that defects in tRNA modifications often cause no or only mild phenotypes [55, 56].

**Figure 7.**
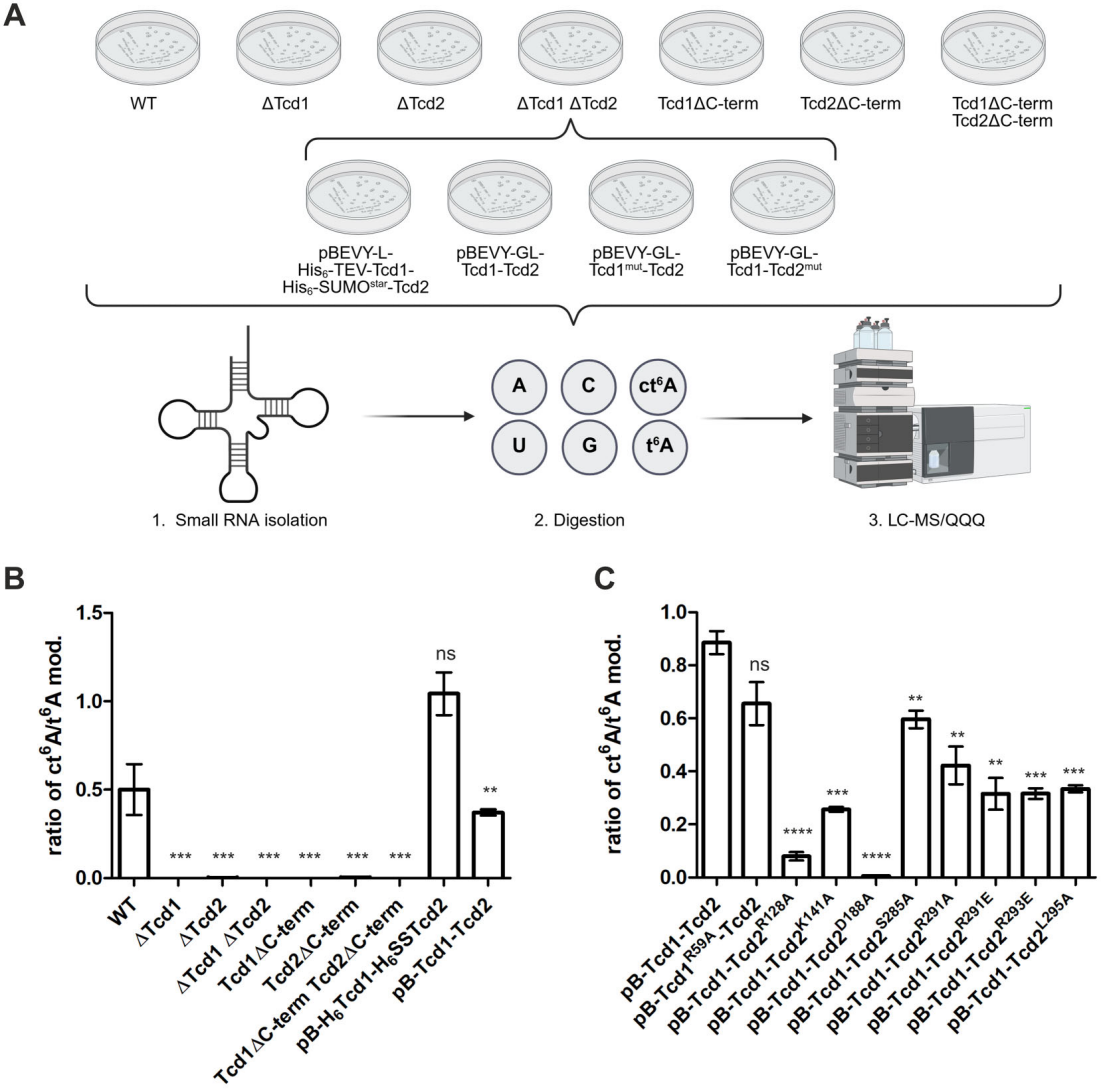
*In vivo* activity assay of *S. cerevisiae* Tcd variants.(**A**) Schematic overview of the assay set-up and the different *S. cerevisiae* strains used. The figure has been created in BioRender. Huber, E. (2026) https://BioRender.com/miungsl based on Sonntag, R. (2026) https://BioRender.com/w92e694. (**B** and **C**) ct^6^A and t^6^A levels quantified in the different *S. cerevisiae* strains are shown as ct^6^A/t^6^A ratios. The *Δtcd1Δtcd2* double knockout, which does not produce ct^6^A (panel B), served as an expression host for various *TCD1* and *TCD2* versions from the pBEVY-(G)L vector (pB). Tested variants were either missing the N-terminal transmembrane segment and carrying an affinity tag (H_6 _= His_6_-TEV; H_6_SS = His_6_-SUMO^star^) or full-length versions without tag (panel B). In addition, full-length tag-free but mutant versions of Tcd1 and Tcd2 were tested for activity (panel C). In brief, yeast cells were lysed via TRIzol, the small RNA fraction was purified, digested to nucleosides and subsequently quantified by LC–MS/QQQ. Each bar represents the mean of three independent samples (for WT in panel B only duplicates) ± standard error of the mean (SEM). Statistical differences between samples were determined with the TTEST function in Excel using the options: two-tailed distribution and two-sample equal variance (homoscedastic). Asterisks indicate *P* values *<0.05, **<0.01, ***<0.001, ****<0.0001. The production of all enzyme variants *in vivo* was verified by probing C-terminally His_7_-tagged versions with western blotting (see Supplementary Fig. S22). For simplicity, labels do not adhere to yeast gene nomenclature and do not discriminate between genes and corresponding proteins.

**Figure 8.**
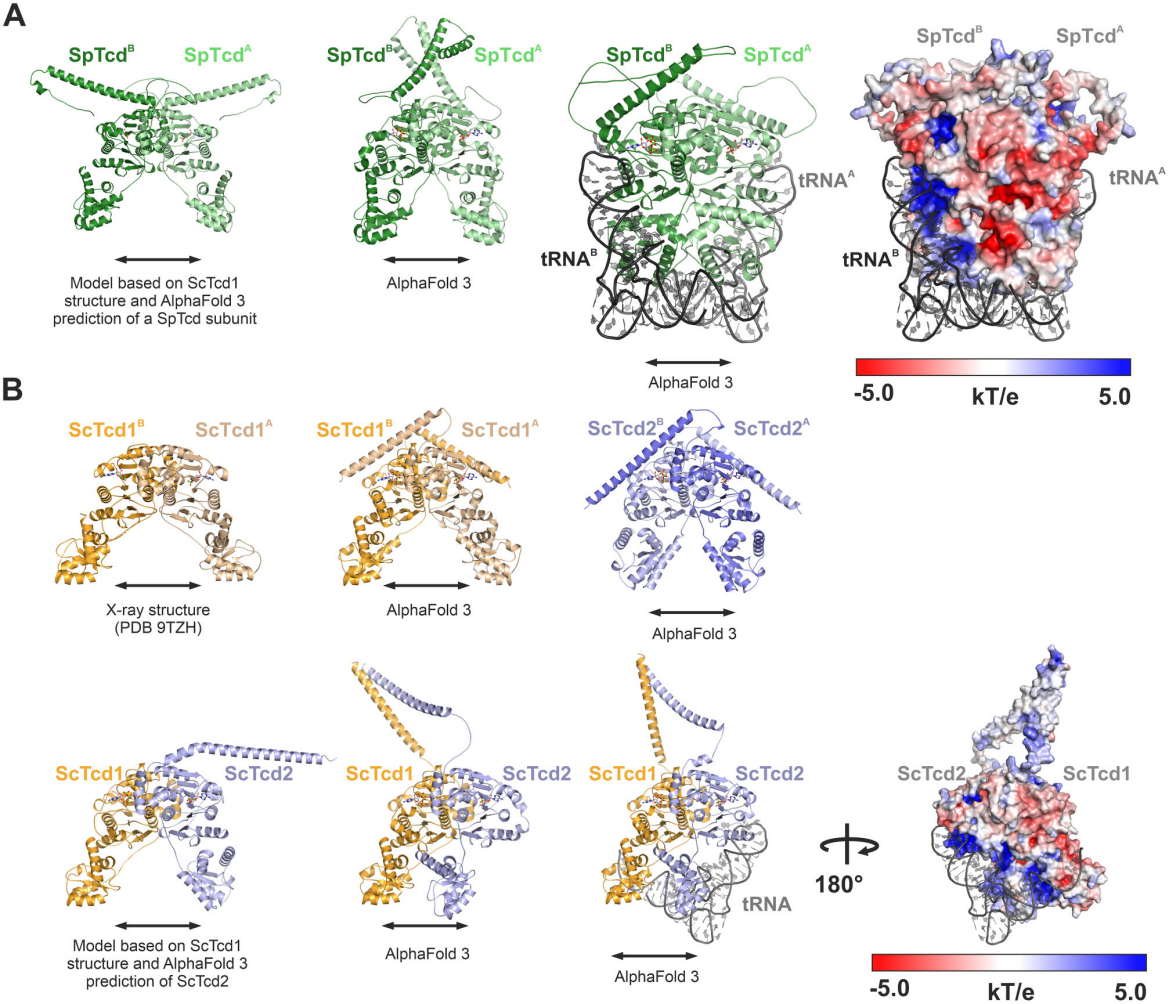
tRNA binding to yeast Tcd enzymes. Cartoon and Connolly surface models of (**A**) *S. pombe* Tcd and (**B**) *S. cerevisiae* Tcd1/2 enzymes generated by AlphaFold 3 [51] (prediction as homo-/heterodimers) or by superimposing single subunit AlphaFold 3 predictions onto the Tcd1 X-ray structure. Models were predicted with the N-terminal transmembrane segment. The distance of the C-terminal domains in the AlphaFold 3 predictions of *S. pombe* Tcd and *S. cerevisiae* Tcd1-Tcd2 (column 2) is shorter than in the Tcd1 X-ray structure (panel B, upper left panel) and even shorter in tRNA bound structures (column 3), indicating a potential conformational change of the C-terminal domains upon tRNA binding (black double arrows). The larger distance of the C-terminal domains in the Tcd1 X-ray structure compared to the AlphaFold 3 prediction may be a result of crystal packing (see Supplementary Fig. S26).

The double knockout strain then served as an expression host for the overproduction of Tcd1 and Tcd2 variants in the context of an *in vivo* activity assay (Fig. 7A). This set-up allowed us to study the activity of Tcd enzymes and the functional contribution of individual residues. Specifically, the yeast strains were grown and small RNAs were isolated and analyzed for their ct^6^A and t^6^A contents by HPLC-MS (Fig. 7B and C). While WT *S. cerevisiae* produced ct^6^A, the double knockout strain *Δtcd1Δtcd2* did not (Fig. 7B). By overproducing native, full-length Tcd1^1-429^ and Tcd2^1-447^ (both with their genuine N-terminal membrane anchor and without tag) or His_6_-TEV-Tcd1^50-429^ and His_6_-SUMO^star^-Tcd2^62-447^ in the double knockout strain, ct^6^A production was clearly restored (Fig. 7B), confirming the activity of Tcd enzymes in *S. cerevisiae*. These data further imply that (i) the membrane anchor is not required for activity; (ii) the affinity tags disturb neither complex assembly nor activity and that (iii) either Tcd1, Tcd2 or both produce ct^6^A. To further refine the results, we tested the single knockout strains *Δtcd1* and *Δtcd2* for ct^6^A production. In agreement with our findings that Tcd2 assembles with Tcd1 into a heterodimer, the single knockouts also failed to produce ct^6^A. To probe the functional relevance of the C-terminal domain for ct^6^A production in yeast, we created strains in which either Tcd1 or Tcd2 or both lacked this segment (Supplementary Fig. S20). To our surprise, all these strains were devoid of ct^6^A (Fig. 7B). While surface charge distributions of Tcd2 (Fig. 6B) surmised that deletion of the Tcd2-C-terminal domain might negatively affect tRNA binding, the need of the Tcd1-C-terminal domain for yeast Tcd activity was not obvious. An AlphaFold 3 model of the Tcd1–Tcd2–tRNA complex however implies that the C-terminal domain of Tcd1 contributes to tRNA binding as well (Fig. 8B). Moreover, the C-terminus of Tcd1 might be required for stability, integrity and solubility of the whole Tcd1 subunit and hence indirectly for ct^6^A production. In agreement, we found that yeast Tcd enzymes lacking the C-terminal domain were not accessible to purification (Supplementary Fig. S21).

Apart from deletion mutants, we investigated the effects of single-point mutants in Tcd1 and Tcd2 on ct^6^A production (Fig. 7C). To this end, we overexpressed Tcd1 and Tcd2 variants (full-length, tag-free versions with the N-terminal transmembrane anchor but with point mutations) in the *Δtcd1Δtcd2* strain (Supplementary Fig. S22) and evaluated the ct^6^A and t^6^A contents (Fig. 7C). First, we tested the Tcd2_K141A_ mutant. Lys141 is conserved in ThiF (Lys83), MoeB (Lys86) and TcdA from *E. coli* (Lys85), in Tcd1 (Lys129), and SpTcd (Lys181) (Supplementary Figs S1 and S23), where it coordinates the β-phosphate of ATP and stabilizes the negative charge emerging upon nucleotide hydrolysis (Fig. 6D) [39, 57]. Compared to the corresponding control strain (double knockout overexpressing WT Tcd1 and Tcd2), the ct^6^A/t^6^A ratio was 71.0% lower in the Tcd2_K141A_ mutant (Fig. 7C), confirming the functional relevance of this lysine residue for ATP-dependent dehydration of t^6^A. Next, we found that the mutation R128A in Tcd2 drastically restricts ct^6^A production (reduction of the ct^6^A/t^6^A ratio by 91.0%, Fig. 7C). Arg128 facilitates nucleotide hydrolysis by coordinating to the phosphate groups of ATP and has functional equivalents in ThiF (Arg70), MoeB (Arg73), TcdA (Arg72), and SpTcd (Arg168) but not in Tcd1 (Fig. 6D and Supplementary Figs S1 and S23). Instead Tcd1 encodes Thr116, that due to its shorter side chain length fails to contribute to ATP activation (Fig. 6D and Supplementary Fig. S1). Additionally, we mutated Arg59 of Tcd1 to Ala. According to our structural model of the Tcd1–Tcd2 heterocomplex, Arg59 of Tcd1 is an arginine finger, completing the Tcd2 active site and chelating the γ-phosphate of Tcd2 bound ATP (Fig. 6D). This arginine finger is also known from ThiF (Arg11) [39] and MoeB (Arg14) [57] proteins (Supplementary Fig. S23). Although arginine fingers are usually essential for the hydrolysis of triphosphates in the neighboring enzyme subunit, the arginine fingers of MoeB and ThiF are not [57, 58]. Consistently, the mutation R59A in Tcd1 did not significantly affect the ct^6^A/t^6^A ratio (reduction by 26.0%, Fig. 7C) and this arginine is also absent from TcdA (Supplementary Figs S1 and S23).

Based on the AlphaFold 3 prediction of the Tcd1-Tcd2-tRNA complex (Supplementary Fig. S18) residues Arg293 and Arg291 of Tcd2 were considered to be relevant for tRNA and potentially t^6^A recognition. Indeed, the Tcd2 mutants R293E (64.3% reduction of the ct^6^A/t^6^A ratio), R291E (64.4% reduction) and R291A (52.3% reduction) significantly impaired the catalytic activity of the Tcd1–Tcd2 heterocomplex (Fig. 7C). Yet, the Tcd2_R293E_ mutant still purified with tRNA (Supplementary Fig. S24), suggesting that tRNA binding per se is not abrogated. Next, we modeled a t^6^A base adjacent to the anticodon of the tRNA in the AlphaFold 3 model and noted that the substrate has to move about 4 Å deeper into the active site to become adenylated, placing t^6^A nearby Asp188 and Leu295 of Tcd2 (Supplementary Fig. S25). To probe a potential function of these residues in substrate orientation and/or catalysis, both amino acids were individually mutated to alanine and the dehydratase activity was assessed by the *in vivo* assay set-up. While in the Tcd2_L295A_ mutant still 37.7% of the WT ct^6^A/t^6^A ratio was found, the Tcd2_D188A_ variant was inactive (0.65% of WT ct^6^A/t^6^A ratio detected), implying that Asp188 plays a key role for the catalytic cycle of Tcd2 and the formation of ct^6^A. The functional relevance of this aspartate is further underpinned by its conservation in ThiF (Asp127), MoeB (Asp130), TcdA (Asp130), SpTcd (Asp226), Tcd1 (Asp177) (Supplementary Figs S1 and S23) as well as two recently identified Tcd2 homologues from *Candida* species [59] (Asp177 and Asp174 in Hma1 (hypermodification of adenosine) from *Candida albicans* and *Candida dubliniensis*, respectively).

## Discussion

Tcd enzymes are responsible for the conversion of the essential tRNA modification t^6^A to the nonessential variant ct^6^A, but only little is known about the cyclization reaction and the corresponding enzymes. Here, we investigated yeast Tcd enzymes (Tcd1/2 from *S. cerevisiae* and Tcd from *S. pombe*) from a structural and biochemical point of view. We showed that yeast Tcds reside in the outer mitochondrial membrane, face the cytosol (Fig. 3), and do not require the transmembrane segment for ct^6^A formation (Fig. 7B), which agrees with the fact that *E. coli* TcdA is not a membrane protein [19].

**Figure 9.**
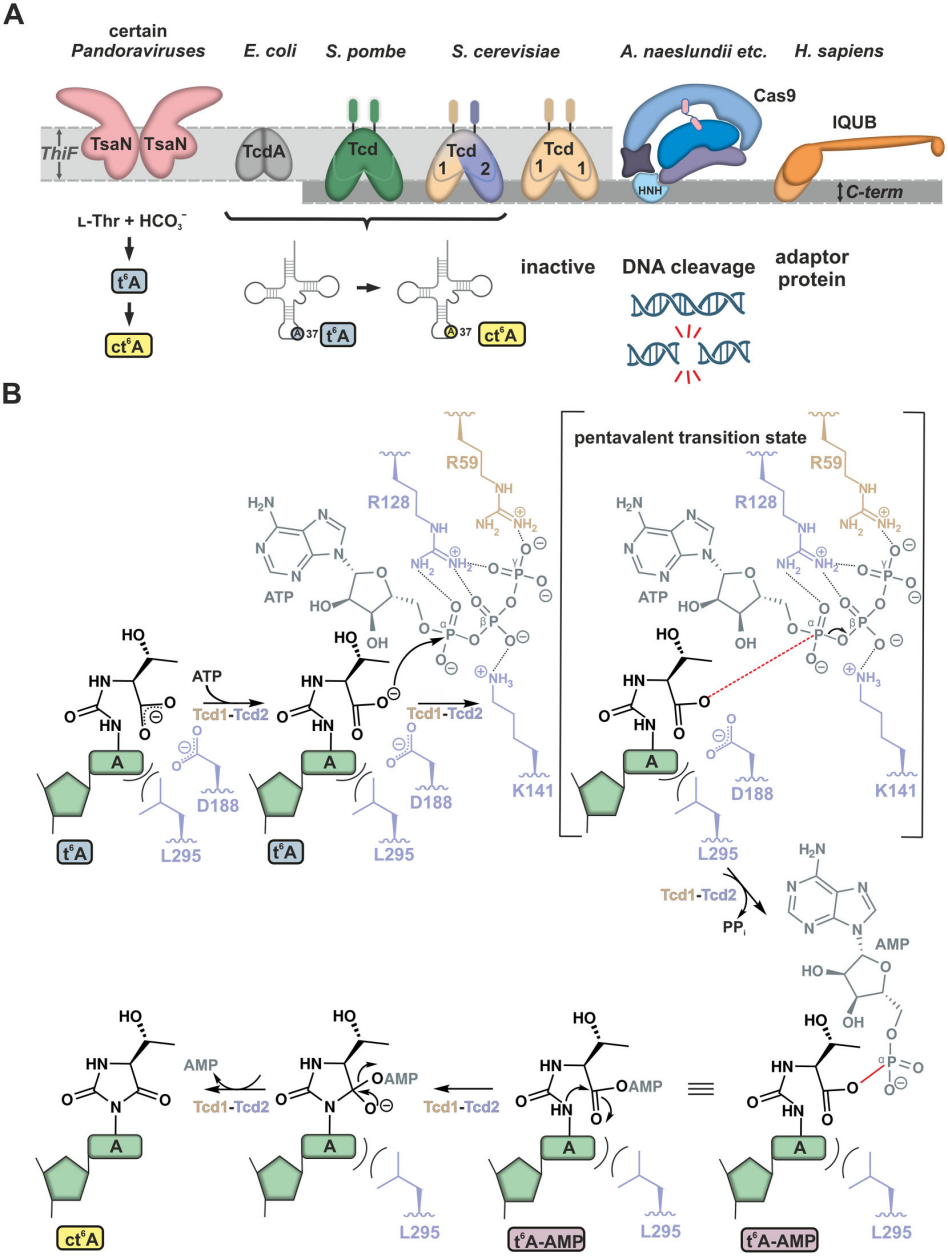
Evolutionary and mechanistic aspects of yeast Tcd enzymes. (**A**) Schematic comparison of Tcd enzymes reported from viruses, bacteria and yeasts (left). All Tcds share the N-terminal ThiF domain. TsaN from pandoraviruses acts on the nucleotide/nucleoside level but not on tRNA. Yeast Tcd enzymes exist as homo- or heterodimers and their C-terminal domain resembles the HNH domain of Cas9 enzymes as well as the C-terminus of IQUB, an adapter protein for the radial spoke 1 complex (right). Parts of this figure have been created in BioRender. Huber, E. (2026) https://BioRender.com/4gxkbsv and https://BioRender.com/h55raqy. For the schematic illustration of Cas9 a similar picture from https://geneticeducation.co.in/cas9-protein-structure-function-types-and-importance/ served as a blueprint. (**B**) The proposed reaction mechanism of ct^6^A formation in yeast relies on proximity and correct positioning of the reactants. Key residues involved in the reaction sequence in *S. cerevisiae* are depicted in light yellow (Tcd1) and light blue (Tcd2), respectively. Asp188 helps polarizing the carboxylic group of t^6^A for the subsequent nucleophilic attack onto ATP. Leu295 provides hydrophobic stabilization of the adenine base, while depicted lysine and arginine residues coordinate the triphosphate. According to Schmelz and Naismith, 2009 [64], the conversion of t^6^A to t^6^A-AMP proceeds via a pentavalent transition state.

Yeast and *E. coli* Tcd enzymes differ in their C-terminal domain architecture (Fig. 1C). In contrast to *E. coli* TcdA, the C-terminal domain of yeast Tcds shares structural similarity with the catalytic HNH domain of CRISPR–Cas9 endonucleases (Fig. 9A) but is likely catalytically inactive. Mutagenesis data imply that the C-terminal domain—like the much smaller and structurally unrelated C-terminal segment of TcdA [19]—is relevant for tRNA binding. Yet, most of the residues involved in tRNA recognition in TcdA are not conserved in the yeast homologues and vice versa (Supplementary Fig. S1), thus leaving the impact of the different C-terminal domains between bacterial and eukaryotic Tcds on substrate specificity and conversion enigmatic. Notably, the C-terminal domain of yeast Tcds is also part of the human protein IQUB [IQ (I, isoleucine; Q, glutamine) motif and ubiquitin domain containing; residues 640–751, Fig. 9A], which anchors the radial spoke 1 complex to the A microtubule in axonemes, required for ciliary movements [60, 61], but the exact function of this structural element in this context is unknown as well.

We here report structural data on Tcd1 that imply a V-shape for yeast Tcd enzymes. Although AlphaFold 3 predictions of yeast Tcd enzymes with and without tRNA are in agreement with this shape, we also noted differences (Fig. 8). According to AlphaFold 3, the C-terminal domains within *S. pombe* Tcd and the *S. cerevisiae* Tcd1–Tcd2 complex are closer together than expected from the Tcd1 X-ray structure. The larger distance in the Tcd1 X-ray structure may be a result of intermolecular contacts made via the C-terminal domain in the crystal lattice (Supplementary Fig. S26). The AlphaFold 3 model of the Tcd1–Tcd2–tRNA complex further indicates that the C-terminal domains may rotate up to 135 ° towards each other after tRNA binding (Fig. 8) and that both undergo extensive tRNA interactions with their positively charged helix α1 (Fig. 2A). This model explains the functional need of both C-terminal domains in the Tcd1–Tcd2 complex for activity (Fig. 7B) and confirms that the phosphate binding site observed in the SpTcd^366-485^ X-ray structure is indeed functionally relevant (Fig. 2B and Supplementary Fig. S5D).

Both yeast and *E. coli* Tcd enzymes can be co-purified with tRNAs. For the yeast enzymes we found that the majority of the co-purified tRNAs carry a NNU anticodon, which matches the reported function of Tcd enzymes to decode ANN codons in mRNAs [16, 19, 20]. The co-purified tRNAs were decorated with t^6^A, ct^6^A or none of the two modifications (Supplementary Fig. S14). While *E. coli* TcdA can be purified without tRNA under high salt conditions [19, 20] (Supplementary Fig. S16), this was not feasible for the yeast enzymes. The reason for this observation is unknown.

Studies with Tcd1 alone revealed that it is unable to associate with tRNAs (Supplementary Fig. S8C) and to convert them (Fig. 7B). The absence of positively charged surface patches (Fig. 4C) explains the inability to bind nucleic acids but questions the need for AMP binding (Fig. 4B). Most likely Tcd1 and Tcd2 arose by gene duplication and subsequent diversification during which the AMP-binding site of Tcd1 (Asp105, Lys129) has been retained. However, the Walker motif of Tcd1 (Gly-x-Gly-Glu-x-Gly versus Gly-x-Gly-Gly-x-Gly) contains an unusual Glu residue that sterically hinders ATP binding (Supplementary Figs S1 and S10G). Additionally, Tcd1 misses Arg128 (replaced by Thr116, Fig. 6D and Supplementary Fig. S1), a residue that has been proven crucial in Tcd2 (Fig. 7C).

Although we here propose that Tcd1 on its own is an inactive Tcd, it is essentially required for the stability and solubility of Tcd2 *in vitro* (Fig. 5 and Supplementary Fig. S7) and for ct^6^A formation (Fig. 7B). This biophysical and functional interdependence of Tcd1 and Tcd2 is explained by the formation of a heterodimer. A structural model of the Tcd1–Tcd2 complex illustrates that Tcd2 provides the basic surface patch required for tRNA binding (Fig. 6B) as well as the catalytic residues Arg128 and Asp188, while Tcd1 complements the Tcd2 active site by contributing—similar as in ThiF [39] and MoeB [57]—a catalytically nonessential arginine finger (Arg59) (Fig. 6D). The fact that the arginine finger is not essential in these proteins may explain why *E. coli* TcdA lacks this structural feature (instead Gly15 is encoded; Supplementary Fig. S1). Nagy *et al*. proposed that the absence of a magnesium ion could affect the position of the arginine finger and hence explain its non-essential character in the proteins of the ThiF and MoeB family [58], but further studies on structure and catalytic parameters will be required to fully dissect the role of the arginine finger in these proteins.

We also inspected the primary sequence of *S. pombe* Tcd and found all key residues (including the arginine finger Arg111) encoded in this single subunit (Supplementary Fig. S1). Considering that *S. pombe* Tcd eluted as a protein with a much larger hydrodynamic radius than expected for its molecular weight (Supplementary Figs S2A, and S28A and B) and that its structure prediction features a positively charged surface patch (Supplementary Fig. S27), we assume that *S. pombe* Tcd forms a homodimer with two tRNA-binding sites and two catalytically active centers (Fig. 6D), similar as *E. coli* TcdA [19, 20].

In summary, Tcd enzymes from organisms that do not encode a second paralog harbor two functional active sites (as a result of their homodimeric assembly), while *S. cerevisiae* has retained only one catalytic center in the Tcd1–Tcd2 heterodimer. Although experimental evidence is lacking, it is tempting to speculate that the inactivation of one active site in the Tcd1–Tcd2 heterodimer might have allowed fine-tuning of Tcd levels and activity. Assuming constitutive and inducible expression of *TCD1* and *TCD2*, respectively, Tcd1 could reside as a nonfunctional homodimer in the outer mitochondrial membrane waiting to team-up with Tcd2 under suitable conditions. In this regard it is also worth to mention that Tcd1 is annotated as a phosphoprotein [62]. Its phosphorylation site, Ser259, is unique to yeast Tcd enzymes and localizes between the ThiF and C-terminal domains. It is therefore well conceivable, that Tcd1 acts as the regulatory subunit for Tcd2, allowing for tighter control of ct^6^A formation.

Tcd enzymes display homology to ThiF, MoeB and ubiquitin-activating E1 enzymes, which all use adenylation as a substrate activation step. Consistently, the ATP-dependent dehydration of t^6^A to ct^6^A has been proposed to involve transfer of an AMP moiety onto t^6^A [19]. According to our structural models and activity assay data, Asp188 is crucial for this adenylation, as it polarizes the carboxy group of t^6^A for its nucleophilic attack onto the α-phosphate of ATP (Fig. 9B). Asp188 is conserved in all yeast Tcds as well as in the *E. coli* proteins TcdA, ThiF, and MoeB (Supplementary Figs S1 and S23). Notably, in ThiF (Asp127) and MoeB (Asp130), this aspartate residue has been predicted to chelate a Mg^2+^ ion [39, 57].

In E1 enzymes, the adenylate is subsequently attacked by a reactive cysteine, leading to a covalently enzyme-linked thioester (Supplementary Fig. S29A). By contrast, although ThiF (Cys184) and MoeB (Cys187) share the same cysteine residues, these do not engage in thioester formation with their adenylated substrate [40, 63]. Instead, both thiamin and molybdenum cofactor biosynthesis use a sulfur transferase to produce a thiocarboxylate intermediate (Supplementary Fig. S29B and C). TcdA has been reported to encode the same cysteine residue (Cys234) as ThiF, MoeB, and E1 enzymes (Supplementary Fig. S23) and a thioester intermediate has been proposed [19] (Supplementary Fig. S29D), but its relevance for ct^6^A formation has so far not been proven. Strikingly, this cysteine residue is absent from yeast Tcds. In fact, SpTcd, ScTcd2, and two recently identified Tcd2 homologs (Hma1) from *Candida* species, that lack paralogs like Tcd1 [59], do not have a single cysteine residue in common that could serve for thioester formation (Supplementary Fig. S1). To exclude that yeast Tcds use a catalytic serine instead, we mutated Ser285 in the crossover loop of Tcd2 to alanine. This serine is also part of SpTcd and Hma1 proteins but not present in Tcd1 and TcdA. Although the mutant had a by 32.7% reduced ct^6^A/t^6^A ratio compared to the corresponding WT strain (Fig. 7C), this rather moderate effect excludes that Ser285 engages in a transient covalent bond during the catalytic cycle of Tcd2. The crossover loop, in which Ser285 is encoded (Supplementary Fig. S1), is a flexible region that in MoeB [57], the ThiS–ThiF complex [65], TcdA [19, 20] and Tcd1 structures is disordered but that is thought to contribute to substrate binding [39]. In agreement, mutations in the crossover loop have been shown to impair ThiF activity [39]. Our Tcd1–Tcd2–tRNA model supports a role of the crossover loop in substrate recognition and even though Ser285 might not directly contact the tRNA, it is possible that altered dynamics of the crossover loop in the Ser285Ala mutant could dampen enzymatic activity.

We therefore propose that yeast Tcds provide a suitable chemical environment that allows for t^6^A adenylation and intramolecular cyclization without the need for a covalent enzyme intermediate (Supplementary Fig. S29E and Fig. 9B). This scenario requires that the protein side chains lining the active site favor a conformation of the adenylated substrate that is compatible with an intramolecular nucleophilic attack of the N6 lone pair onto the carbonyl carbon atom of the threonine side chain of adenylated t^6^A (Supplementary Fig. S29E and Fig. 9B).

Irrespective of any structural (C-terminal extension or not) or mechanistic differences (catalytic residue or not) between yeast Tcds and *E. coli* TcdA, both seem to rely on tRNA-bound t^6^A as substrate. At least TcdA does not convert chemically synthesized t^6^A [19] and we here provide evidence that yeast Tcds do not convert tRNA stem loops. In contrast, an evolutionary ancient prototype of Tcd enzymes, discovered in some Pandoraviruses, was reported to tRNA-independently catalyze the production of ct^6^A (Fig. 9A). On top of that, this enzyme, dubbed TsaN, uses its modular four-domain structure to build not only ct^6^A from t^6^A but also t^6^A from l-Thr, carbonate, and ATP [66]. While the biological function of TsaN for infection cycles of Pandoraviruses still remains elusive, the formation of ct^6^A has recently been shown to be required for virulence and host-induced filamentation of *Candida albicans*, one of the most relevant human pathogenic fungi. Since the corresponding Tcd enzyme Hma1 is absent from humans, its inhibition could offer new avenues for antifungal drug therapies in the future [59].

## Supplementary Material

gkag376_Supplemental_File

## Data Availability

Coordinates and structure factors have been deposited in the Protein Data Bank (entry codes for *S. pombe* Tcd^366-485^: 9HMO (https://doi.org/10.2210/pdb9hmo/pdb) and 9TZG (https://doi.org/10.2210/pdb9tzg/pdb); entry codes for *S. cerevisiae* Tcd1^50-429^: 9HMP (https://doi.org/10.2210/pdb9hmp/pdb) and 9TZH (https://doi.org/10.2210/pdb9tzh/pdb); see also Supplementary Table S1).
