# Supplementary material for: Structural and biochemical characterization of yeast Tcd enzymes installing the post-transcriptional modification ct6A in tRNA
Source: Nucleic Acids Res. 2026 May 5;54(9):gkag376. doi: 10.1093/nar/gkag376 (PMC13139853; doi:10.1093/nar/gkag376)
Supplement: gkag376_Supplemental_File [file gkag376_supplemental_file.pdf]

Structural and biochemical characterization of yeast Tcd enzymes installing the post-transcriptional modification ct^{6}A in tRNA

Julia Hirschmann[1,#], Rachel Sonntag[1,#], Matthias Heiss[2], Ewa Wegrzyn[2], Wolfgang Heinemeyer[1], Thomas Carell[2], Eva M. Huber[1,*]

[1]Technical University of Munich, TUM School of Natural Sciences, Center for Functional Protein Assemblies, 85747 Garching, Germany

[2]Ludwig-Maximilians University Munich, Department of Chemistry and Pharmacy, 81377 München, Germany

[*]correspondence: eva.huber@tum.de
[#]These authors contributed equally to this work

Table of Contents

1. Supplementary figures **S2**

2. Supplementary tables **S42**

3. Supplementary references **S72**

SUPPLEMENTARY FIGURES

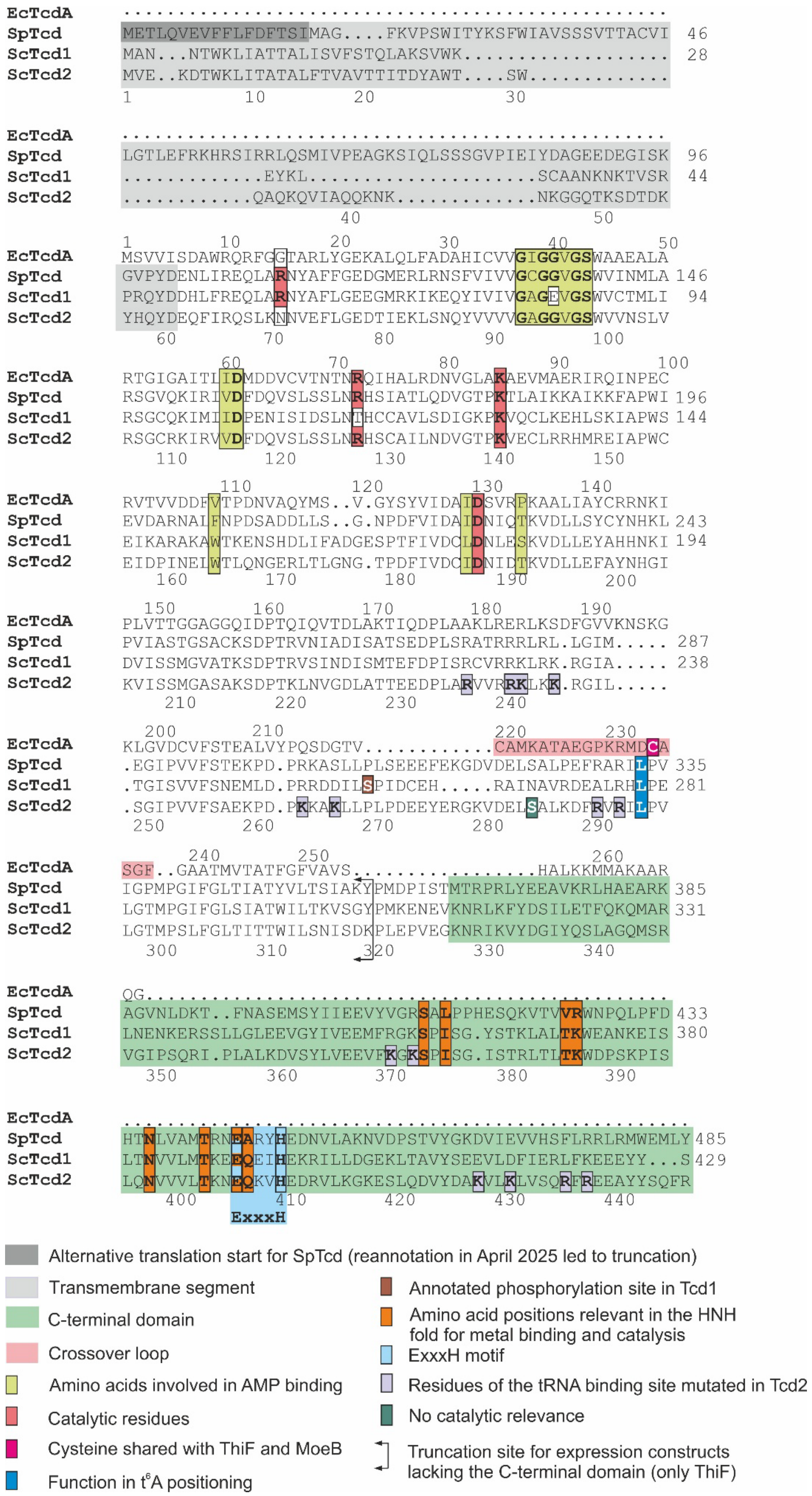

Supplementary Figure 1: Sequence alignment of TcdA (*E. coli*), SpTcd (*S. pombe*), ScTcd1 (*S. cerevisiae*) and ScTcd2 (*S. cerevisiae*) in the one-letter code. Each sequence is numbered. The color legend is shown at the bottom of the figure. Please note that the original sequence of *S. pombe* Tcd shown here has been re-annotated in Uniprot in April 2025, leading to a protein sequence that is N-terminally truncated by 17 amino acids (see dark gray N-terminal segment).

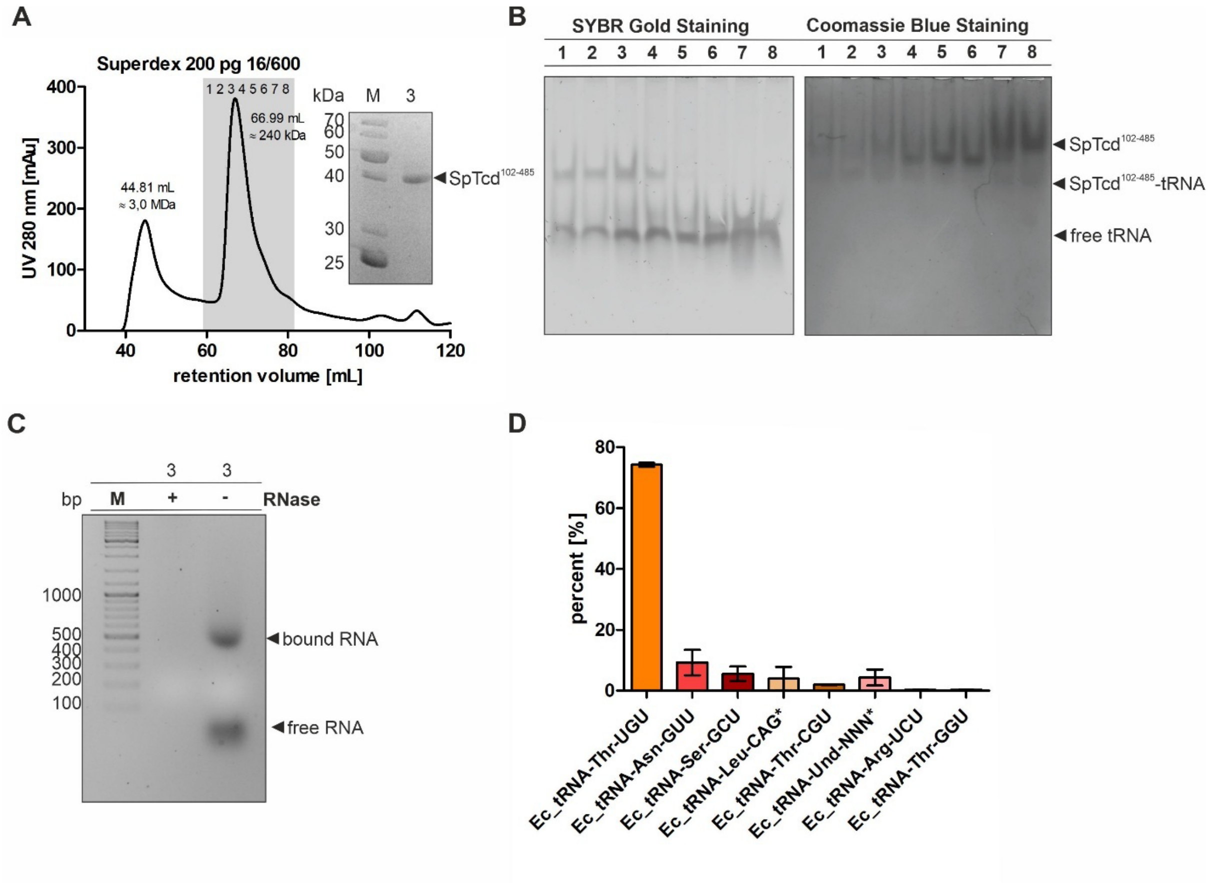

Supplementary Figure 2: Purification of *S. pombe* Tcd$^{102\text{-}485}$ from *E. coli.*

(A) Superdex 200 pg 16/600 size exclusion chromatogram of SpTcd purification. The tailing of the peak at ~67 mL is a result of two overlapping peaks (see also panel B). The apparent relative molecular mass (M_r) of SpTcd calculated from the calibration curve in Supplementary Figure 28B exceeds the theoretic molecular weight for a dimeric Tcd enzyme with two tRNAs bound (~146 kDa). Since SpTcd$^{102\text{-}485}$ is heavily prone to aggregation, the increased M_r value might reflect the tendency of SpTcd$^{102\text{-}485}$ to form even tetramers or higher order oligomers. A fraction from sample 3 was analyzed by SDS-PAGE (12%), and revealed a band at ~40 kDa corresponding to Tcd$^{102\text{-}485}$ (43.1 kDa). (B) Samples from the peak in panel A were analyzed by native PAGE. Staining with SYBR Gold for RNA and Coomassie Blue for protein revealed bands at the same height, indicating co-purification of Tcd with *E. coli* tRNA in the front peak (samples 1-4). By contrast, samples 5-8 contained mostly Tcd without bound tRNA. (C) Agarose gel (2% (w/v)) analysis of sample 3 from the size exclusion chromatography shown in panel A with and without RNAse treatment. After digestion with RNAse (1 mg/mL) for 15 min the bands disappear, confirming the presence of a ribonucleic acid. (D) Next-generation sequencing of tRNAs co-purified with SpTcd. tRNA$^{Thr(UGU)}$ accounted for about 75% of total tRNAs. Additionally, small quantities of tRNA$^{Asn(GUU)}$ and tRNA$^{Ser(GCU)}$ were identified. Shown is a mean value from two independent measurements ± standard error of the mean (SEM). Undefined ('Und') tRNAs or tRNAs with codons other than NNU are most likely non-specifically bound and marked with an asterisk.

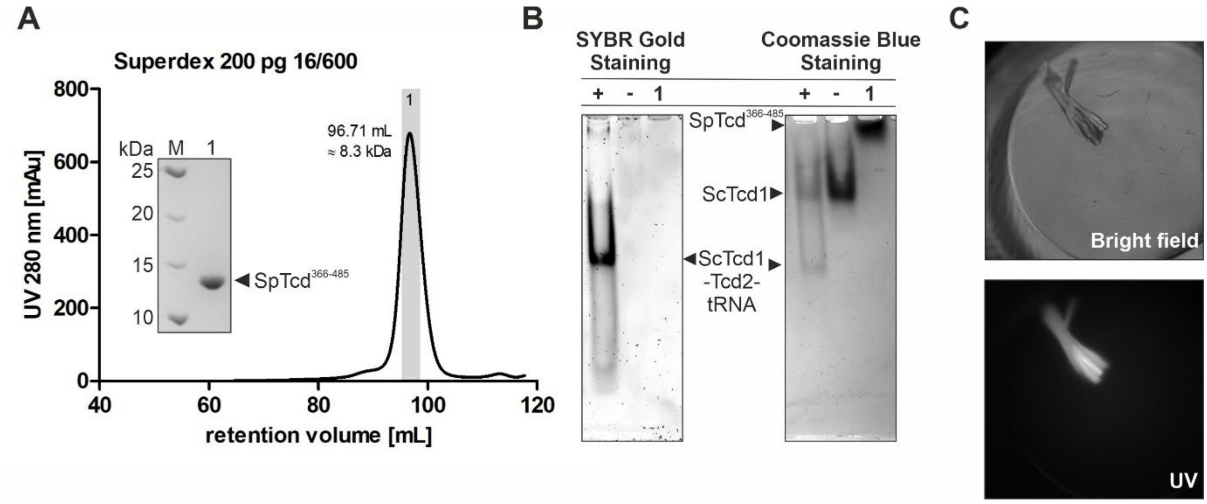

Supplementary Figure 3: Purification of *S. pombe* Tcd$^{366-485}$ from *E. coli.*

(A) Superdex 200 pg 16/600 size exclusion chromatography profile of SpTcd$^{366-485}$. Peak fractions corresponding to the retention volume of 96.71 mL were pooled and used for crystallization. The M_r value was calculated from the calibration curve in Supplementary Figure 28B. SDS-PAGE (15%) revealed a distinct band below 15 kDa, corresponding to the molecular weight of SpTcd$^{366-485}$ (14.2 kDa). (**B)** Native PAGE for SpTcd$^{366-485}$ (lane 1), stained with SYBR Gold for RNA and Coomassie Blue for protein. Purified ScTcd1-Tcd2-tRNA complex served as a positive (+) and purified ScTcd1 as a negative control (-). SpTcd$^{366-485}$ revealed only a band in the Coomassie Blue staining, indicating no tRNA binding. **(C)** SpTcd$^{366-485}$ crystals were grown from a solution containing a 1:1 ratio of protein (10 mg/mL) and 0.2 M NaCl, 0.1 M citrate pH 5.5, 1 M $(NH_4)_2HPO_4$, final pH 7.6

A

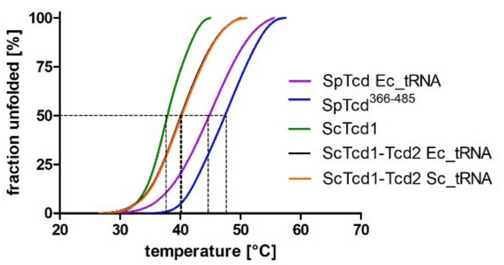

B

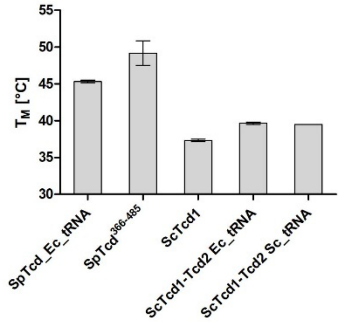

C

ScTcd1-Tcd2 Ec_tRNA

	1	2	3	4	5	6	7	8	9	10	11	12
A	40	40,5	40,5	40,5	40,5	40,5	39	39	39	39	39	39
B	40,5	41	41	41	42	42	39	39	39	39,5	39,5	40,5
C	37,5	39,5	41	41	40	39	37	38	37,5	38	37	36
D	38	38,5	34,5	38	38	38	35,5	35,5	36	36	36	35,5
E	36	36	36	36,5	35,5	35,5	31	32	31	28,5	31	29,5
F	36	35,5	36,5	35,5	35,5	35	28	31,5	32	31,5	32	32
G	35,5	35,5	35,5	35	31	34	30,5	31,5	30,5	30,5	31	31
H	34,5	34,5	34,5	33,5	32	32	30,5	28	29,5	29,5	29	29,5

D

ScTcd1

	1	2	3	4	5	6	7	8	9	10	11	12
A	39	40	40	40,5	40,5	41	37,5	38	38,5	39	39	39,5
B	40,5	41	41,5	42	42	42,5	38,5	39	39,5	40	40,5	41
C	39	39,5	40	40,5	41,5	41,5	37	37,5	38	39	39	37,5
D	38,5	38,5	38	39	37,5	37,5	37	36,5	36,5	37	37	37
E	35	34,5	35,5	35,5	35	35,5	33	33	32	30,5	32,5	31,5
F	35,5	35,5	36	35,5	35,5	35	30	33,5	32,5	32	33	33,5
G	34,5	35,5	35	34	30	35	31,5	31,5	31,5	31,5	31,5	32
H	32	33,5	32,5	33	32	32	30	29	30	30	30	30

E

ScTcd1-Tcd2 Ec_tRNA with 5 mM ATP and 5 mM $MgCl_2$

	1	2	3	4	5	6	7	8	9	10	11	12
A	40,5	41,5	41,5	41,5	41,5	42	39,5	40	40	40	40	40
B	41,5	42	42,5	42,5	43	43	40,5	41	41	41,5	41,5	41,5
C	41	41,5	42	42,5	43	43	39,5	40	40,5	40,5	40,5	39,5
D	36	36	35	36	36	36	36	33	34	35	35	35
E	34	34,5	35	34,5	34,5	35	30,5	31	30,5	29	31	30
F	35	34,5	35	35	34,5	34	30	31,5	31	30,5	32	31,5
G	34	34,5	34	31,5	28,5	32	30,5	31	30,5	30,5	31	30,5
H	34	32,5	31,5	31,5	31	31	30,5	27,5	30	30	30	30,5

F

	1	2	3	4	5	6	7	8	9	10	11	12	
A													100 mM MES pH 5.5
B													100 mM MES pH 6.0
C													100 mM MES pH 6.5
D													100 mM MES pH 7.0
E													100 mM TRIS pH 7.5
F													100 mM TRIS pH 8.0
G													100 mM TRIS pH 8.5
H													100 mM TRIS pH 9.0
	50	100	150	200	250	300	50	100	150	200	250	300	mM NaCl
	10	10	10	10	10	10	0	0	0	0	0	0	% Glycerol

Supplementary Figure 4: Thermal shift assays of purified yeast Tcd enzymes. **(A)** Melting curves represent the normalized average of three measurements per protein preparation. The black curve is congruent with the orange one. Note that SpTcd, SpTcd$^{366\text{-}485}$ and ScTcd1 were tested for their thermostability in Tris buffer at pH 7.5, while for ScTcd1-Tcd2 an optimized buffer with Bis-Tris pH 6.5 was used (see Supplementary Table 17). **(B)** Melting temperatures deduced from the inflection points of the melting curves from panel A are shown as mean ± SEM. **(C-F)** In the course of optimizing the protein purification procedure, buffer screens were performed under different conditions (see panel F). From the heat maps illustrating the melting temperatures of different proteins under various conditions (panels C-E), it becomes evident, that the thermostability of Tcd enzymes is increased at slightly acidic pH and in the presence of 10% (v/v) glycerol, as well as 5 mM $MgCl_2$ and 5 mM ATP. Based on this experiment, the purification buffers for Tcd1 and the Tcd1-Tcd2-tRNA complex were adapted (see Supplementary Table 17). However, ATP was not included as its UV absorbance at 260 nm would have interfered with protein detection at 280 nm.

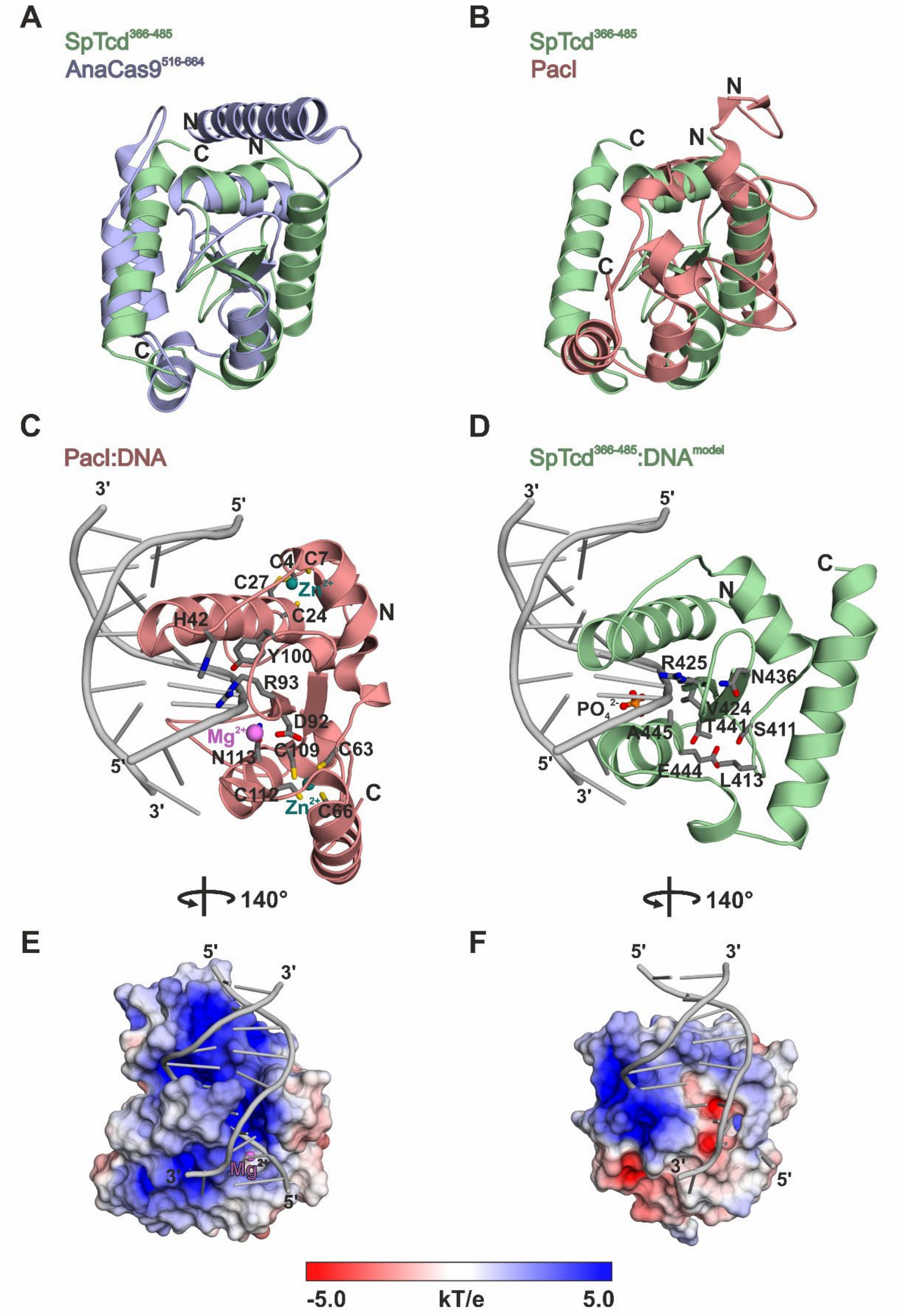

Supplementary Figure 5: Comparison of SpTcd$^{366\text{-}485}$ with its closest structural relatives (Supplementary Table 2).

(A) Superposition of SpTcd$^{366\text{-}485}$ with the Cas9 endonuclease from *Actinomyces naeslundii* (AnaCas9, PDB code 4OGC (1)). **(B)** Superposition of SpTcd$^{366\text{-}485}$ with the restriction endonuclease PacI (PDB code 3LDY (2)), for which only the protein is shown. **(C)** Ribbon illustration of the PacI:DNA complex (PDB code 3LDY (2)). PacI

features two $(CxxC)_2$ zinc fingers and has a magnesium ion bound at the active site. The catalytic residues are His42, Arg93 and Tyr100 (2). **(D)** Modelling of a $SpTcd^{366-485}$-DNA complex by superposition of the PacI:DNA structure onto $SpTcd^{366-485}$. For clarity the PacI protein is not shown. Arg425 of $SpTcd^{366-485}$ is positioned similarly as Arg93 of PacI. Nonetheless, $SpTcd^{366-485}$ lacks the catalytic Tyr and His of PacI and does not encode any equivalent residues, implying that $SpTcd^{366-485}$ is likely catalytically inactive. However, the phosphate ion co-crystallized with $SpTcd^{366-485}$ localizes in close proximity to the modelled DNA, suggesting that the nucleic acid binding region between PacI and $SpTcd^{366-485}$ might be conserved. **(E)** Electrostatic surface potential (-5.0 kT/e (red) to 5.0 kT/e (blue)) for the PacI-DNA complex shown in panel C. **(F)** Electrostatic surface potential (-5.0 kT/e (red) to 5.0 kT/e (blue)) for the modeled $SpTcd^{366-485}$-DNA complex shown in panel D. $SpTcd^{366-485}$ has less positive surface charges compared to PacI.

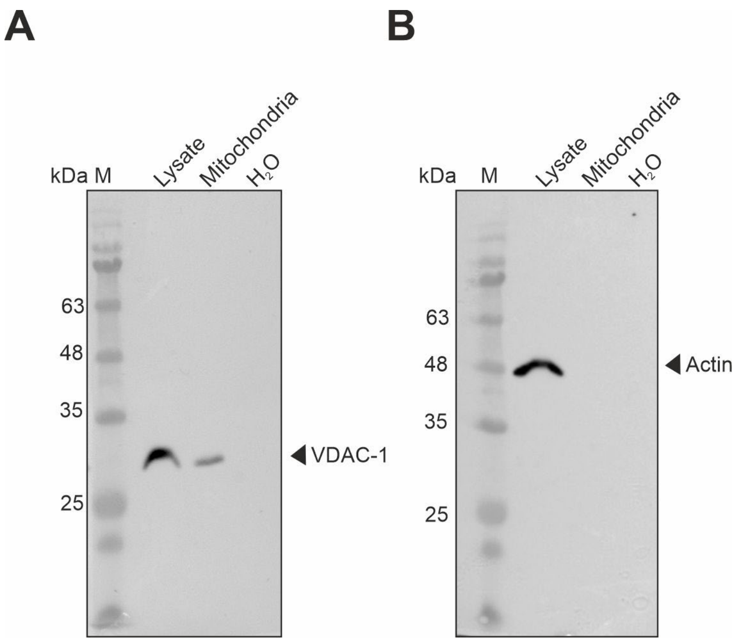

Supplementary Figure 6: Control Western blots for yeast mitochondria isolation. Whole cell lysate and purified mitochondria of the WT *S. cerevisiae* strain BY4741 were probed for the presence of the mitochondrial marker VDAC-1 (A) or the cytosolic protein actin (B). Water served as a negative control for potential background signals. As expected VDAC-1 (theoretical molecular weight: 30.4 kDa) was present in whole cell lysate and in the isolated mitochondria, while actin (theoretical molecular weight: 41.7 kDa) was detectable only in the lysate but not in mitochondria, excluding any contamination of the mitochondrial fraction with cytosolic proteins.

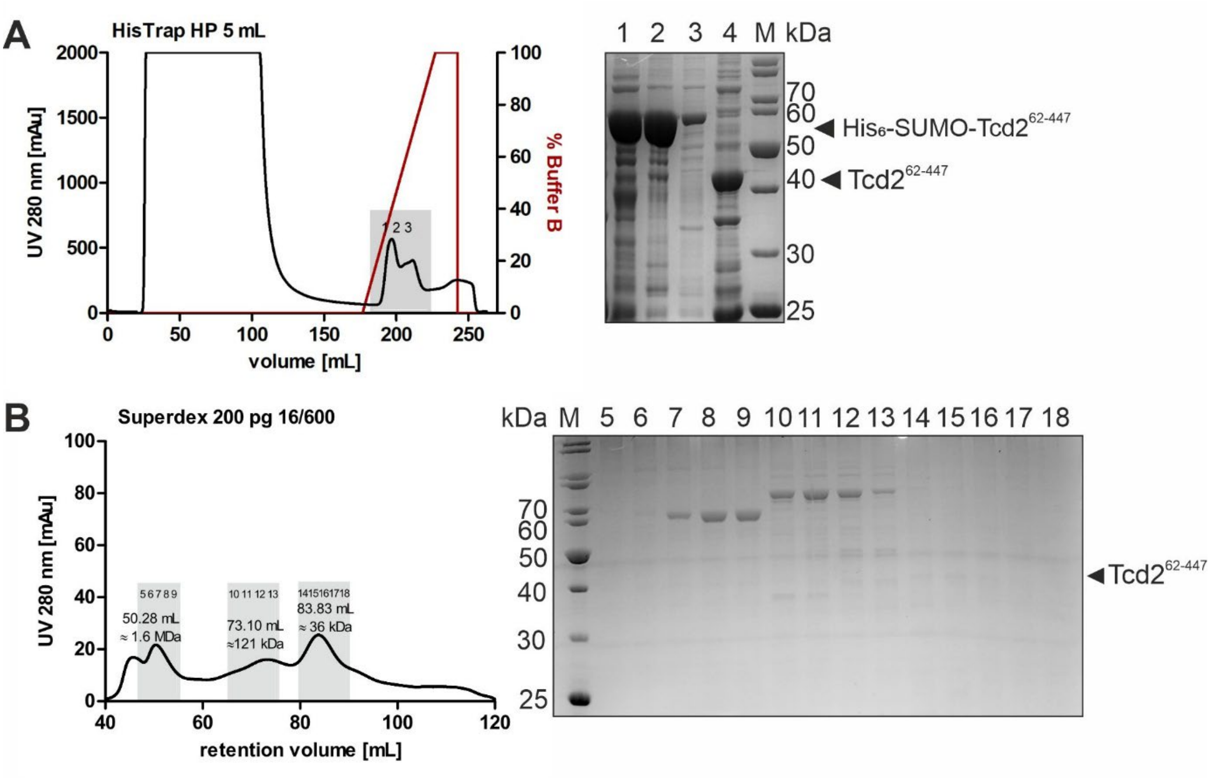

Supplementary Figure 7: Purification of *S. cerevisiae* Tcd2$^{62\text{-}447}$ from *E. coli*.

(A) Chromatogram of the 1. Ni affinity purification (left) and the corresponding SDS-PAGE (right, 12%). Peak fractions 1-3 show a clear band that corresponds to His$_6$-SUMO-tagged Tcd2$^{62\text{-}447}$ (56.8 kDa). After digestion of the pooled fractions 1-3 with SUMO protease (lane 4), a pronounced band corresponding to the molecular weight of Tcd2$^{62\text{-}447}$ (43.3 kDa) is visible. **(B)** Superdex 200 16/600 size exclusion chromatogram (left) and SDS-PAGE (right, 12%) of Tcd2$^{62\text{-}447}$. Neither fraction from the three peaks contained a band of 43.3 kDa size corresponding to Tcd2$^{62\text{-}447}$. M_r values were calculated from the calibration curve in Supplementary Figure 28B.

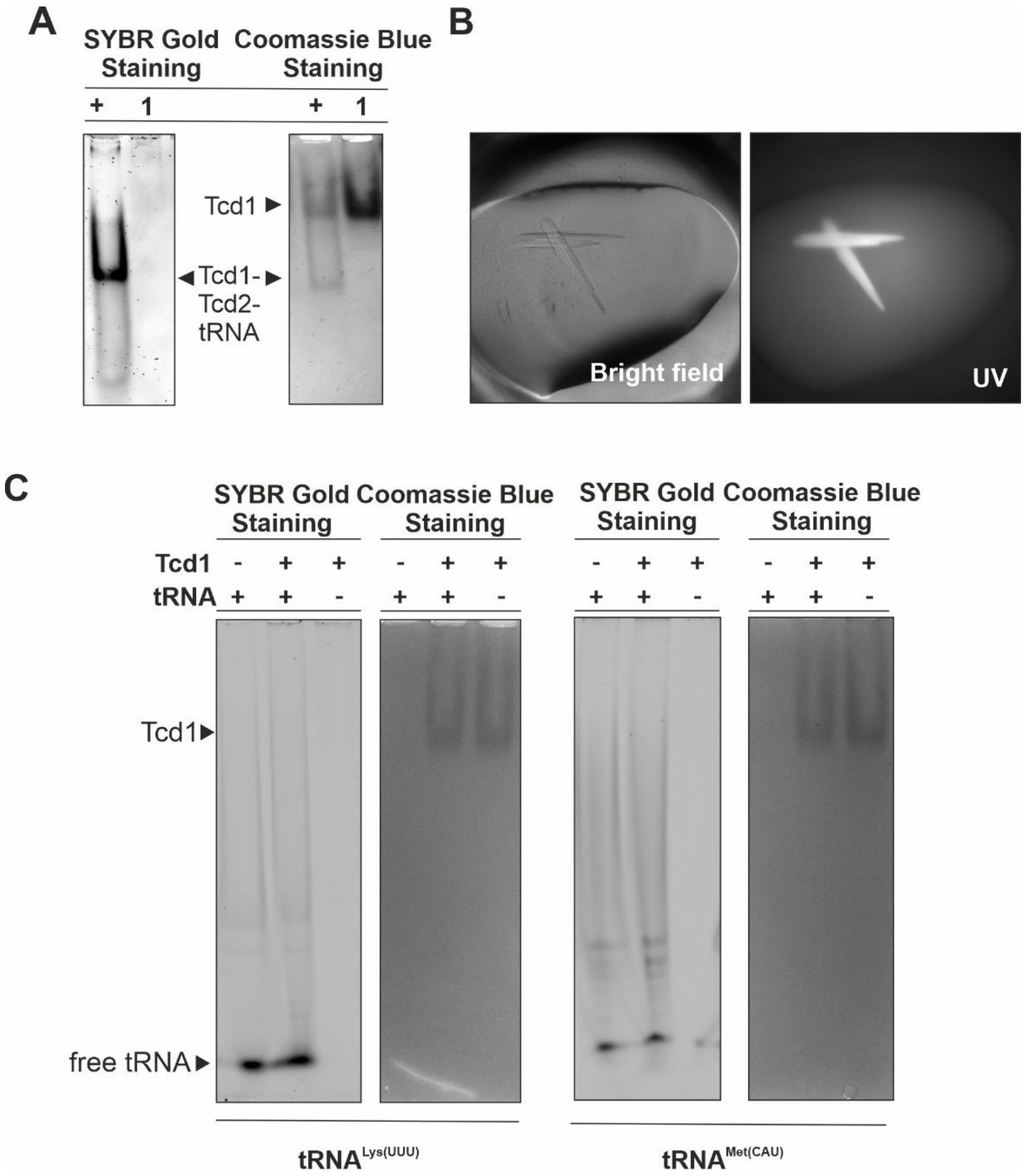

Supplementary Figure 8: Biophysical analysis and crystallization of *S. cerevisiae* Tcd1.

(A) Native PAGE stained with SYBR Gold for RNA and Coomassie Blue for protein; Tcd1 (lane 1) revealed only a band in the Coomassie Blue staining, indicating no tRNA binding. Purified Tcd1-Tcd2-tRNA complex (+) served as a positive control. **(B)** Tcd1 crystals were grown from a 2:1 mixture of protein (20 mg/mL), 3 mM AMP and 1.98 M sodium malonate pH 7.0. **(C)** EMSA of purified Tcd1 (10 µg), mixed with two different *in vitro* transcribed tRNAs (200 ng). Both SYBR Gold and Coomassie Blue staining are shown. For tRNA gene sequences see Supplementary Table 15.

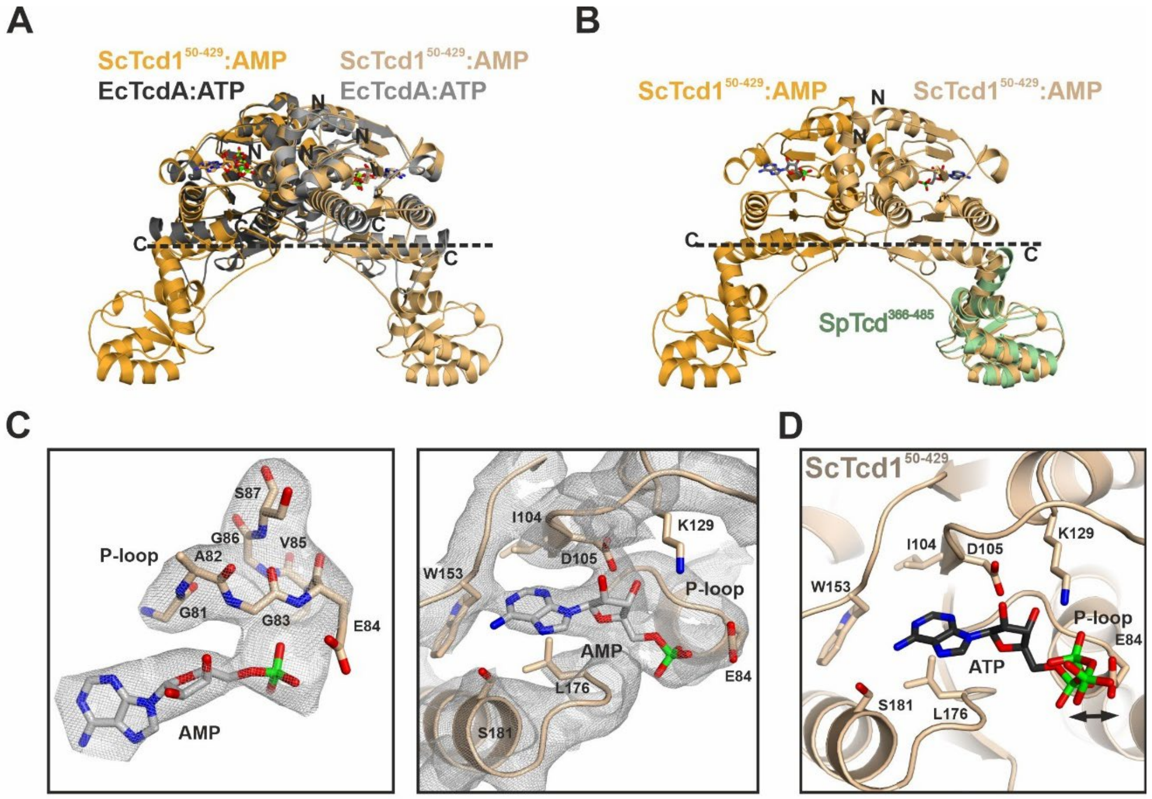

Supplementary Figure 9: Comparison of the Tcd1 homodimer from *S. cerevisiae* with *E. coli* TcdA and Tcd$^{366\text{-}485}$ from *S. pombe*.

(A) Superposition of the Tcd1 homodimer (orange) with the TcdA homodimer (grey, PDB ID: 4D79 (3)) illustrates their structural similarity. TcdA perfectly fits the N-terminal domain (residues 50-301) of Tcd1 (above the dotted line, r.m.s.d. 1.2 Å over 337 C$^{\alpha}$ atoms; sequence identity 22.1%, sequence similarity 43.1%). **(B)** Superposition of Tcd$^{366\text{-}485}$ from *S. pombe* (green) with *S. cerevisiae* Tcd1 (orange). The C-terminal domains from *S. pombe* Tcd (residues 366-485) and *S. cerevisiae* Tcd1 (residues 312-429) are structurally highly similar to each other (r.m.s.d. 1.97 Å over C$^{\alpha}$ 100 atoms; sequence identity 24.0%, sequence similarity 47.9%). (**C**) The mF$_O$-DF$_C$ Polder omit map (4) (gray mesh, contoured to 4 σ) for AMP and the P-loop (residues 81-87) is shown on the left. On the right the 2F$_O$-F$_C$ electron density map (gray mesh, contoured to 1 σ) for AMP and the surrounding protein chain is depicted. Both maps have been calculated from the PDB entry 9TZH.

(D) Structural model for ATP binding to Tcd1. ATP has been modelled into the cofactor binding pocket of Tcd1 by superposition with the TcdA:ATP structure (PDB code 4D79 (3)). For clarity, the TcdA protein is not shown. The model indicates that ATP binding to Tcd1 might be hindered by steric clashes with Glu84 (black double arrow).

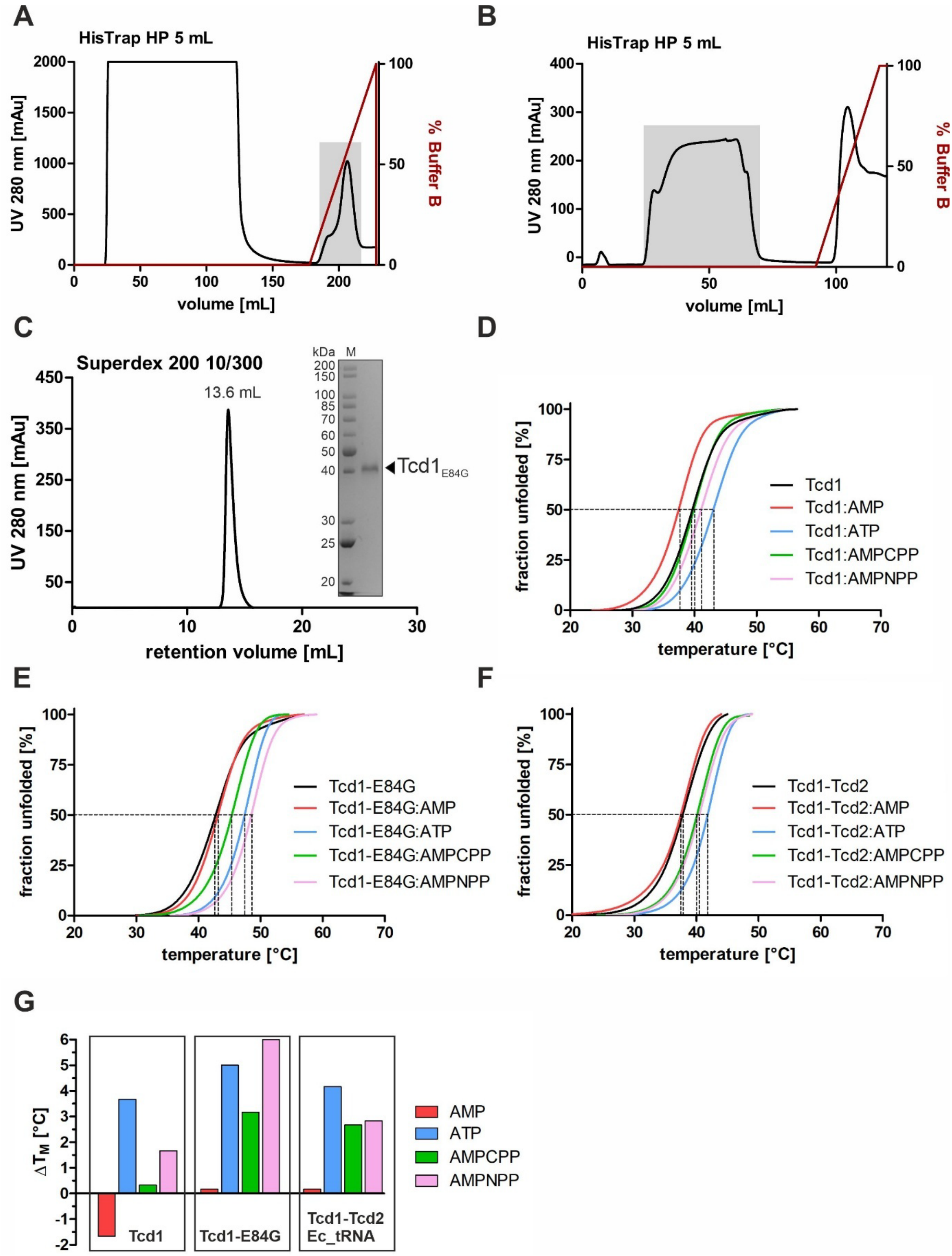

Supplementary Figure 10: Purification and analysis of the Tcd1$^{50\text{-}429}$-E84G mutant.

(A-C) To test the impact of Glu84 on nucleotide binding to Tcd1, a Tcd1-E84G mutant was created and purified by (A) Ni affinity chromatography, followed by tag removal with TEV protease, (B) reverse Ni affinity chromatography and (C) size exclusion

chromatography (Superdex 200 Increase 10/300 GL column). Fractions coloured in grey were pooled and used for the next chromatographic step. The purity and correct size of the sample was confirmed by SDS-PAGE (calculated molecular weight: 43.4 kDa). **(D-F)** Thermal shift assays of purified (D) *S. cerevisiae* Tcd1, (E) Tcd1-E84G and (F) Tcd1-Tcd2-tRNA complex in the absence or presence of 2 mM of the indicated nucleotide (analog). The Tcd1-Tcd2-tRNA complex was obtained from the purifications shown in Figure 5 and Supplementary Figure 12. Melting curves represent the normalized average of three measurements per protein preparation. The assay was conducted for all proteins in 100 mM Bis-Tris pH 6.5, 150 mM NaCl, 5 mM $MgCl_2$, 10 % (v/v) glycerol, 3 mM DTT. **(G)** Differences in melting temperatures deduced from panels D-F are shown normalized to the respective protein without nucleotide. The results indicate that the Tcd1-E84G mutant is stabilized much better by ATP, AMPCPP or AMPNPP compared to WT Tcd1, supporting the proposed role of Glu84 in hindering ATP binding to Tcd1.

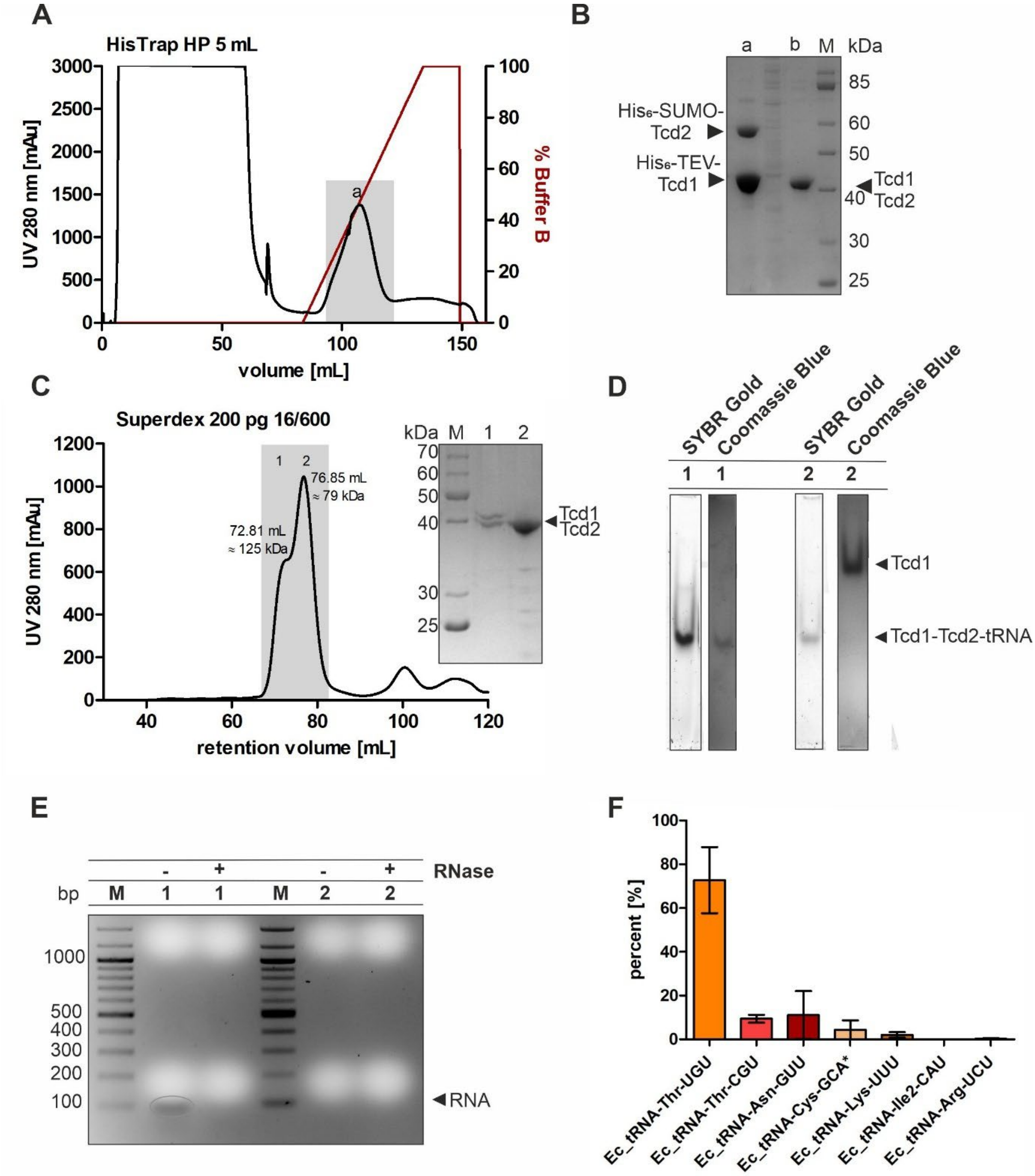

Supplementary Figure 11: Co-purification of *S. cerevisiae* Tcd1$^{50\text{-}429}$ and Tcd2$^{62\text{-}447}$ from *E. coli*.

(A) Ni affinity chromatography profile. **(B)** SDS-PAGE (12%) confirmed the presence of His$_6$-SUMO-Tcd2$^{62\text{-}447}$ (56.8 kDa) and His$_6$-TEV-Tcd1$^{50\text{-}429}$ (45.5 kDa) in sample a. After tag cleavage (lane b), both proteins run as a single band at about 40 kDa (Tcd1$^{50\text{-}429}$: 43.5 kDa, Tcd2$^{62\text{-}447}$: 43.3 kDa). **(C)** Superdex 200 16/600 size exclusion chromatogram. M_r values were calculated from the calibration curve in Supplementary Figure 28B. A 12% SDS-PAGE revealed a double band in the front peak (presumably Tcd1-Tcd2 with tRNA (~117 kDa)) and a single band in the rear peak (presumably a

Tcd1 dimer only (~87 kDa)). **(D)** Native PAGE with protein samples 1 and 2 from the size exclusion chromatography shown in panel C. While sample 1 shows bands at the same height in the SYBR Gold and Coomassie Blue staining, indicating co-purification of a nucleic acid, sample 2 generates only a light band in the SYBR Gold staining (originating from the overlap of peaks 1 and 2 in the size exclusion chromatogram shown in panel C) and a significant protein band that does not co-migrate with a nucleic acid. **(E)** Agarose gel (2%) performed with protein samples 1 and 2 from the size exclusion chromatography shown in panel C. The co-purified nucleic acid in sample 1 can be removed by RNase proving its RNA nature. **(F)** Next-generation sequencing revealed that $tRNA^{Thr(UGU)}$ accounted for more than 70% of total tRNAs present in sample 1. $tRNA^{Thr(GCU)}$ and $tRNA^{Asn(GUU)}$ were detected in lower quantities. Shown is the mean value from two independent biological samples ± SEM. tRNAs with codons other than NNU are most likely non-specifically bound and marked with an asterisk.

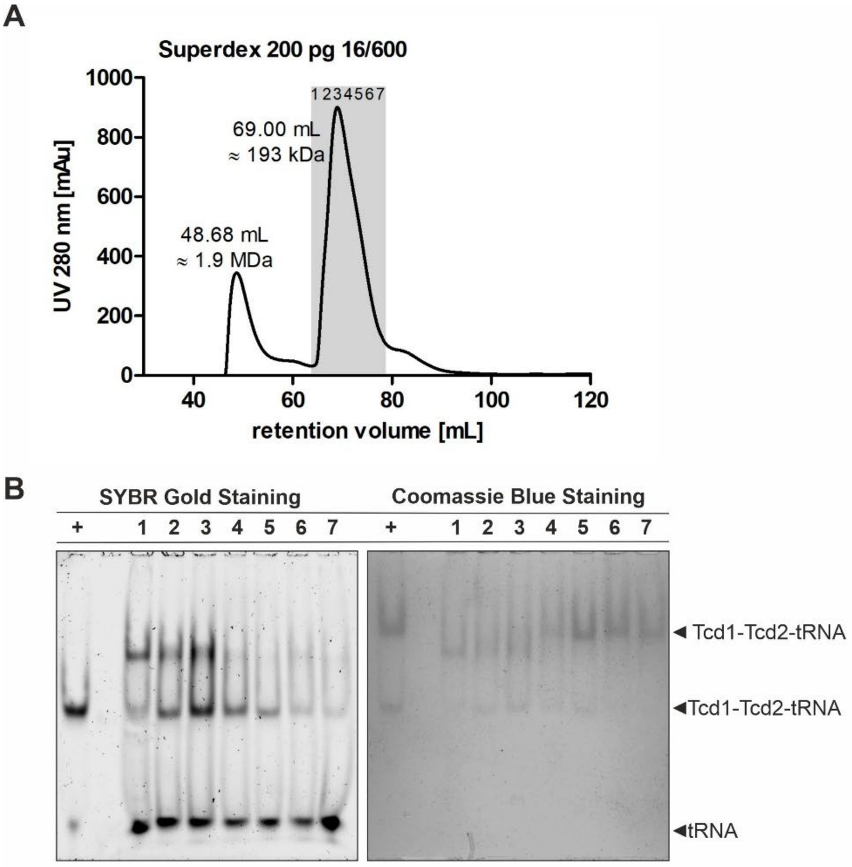

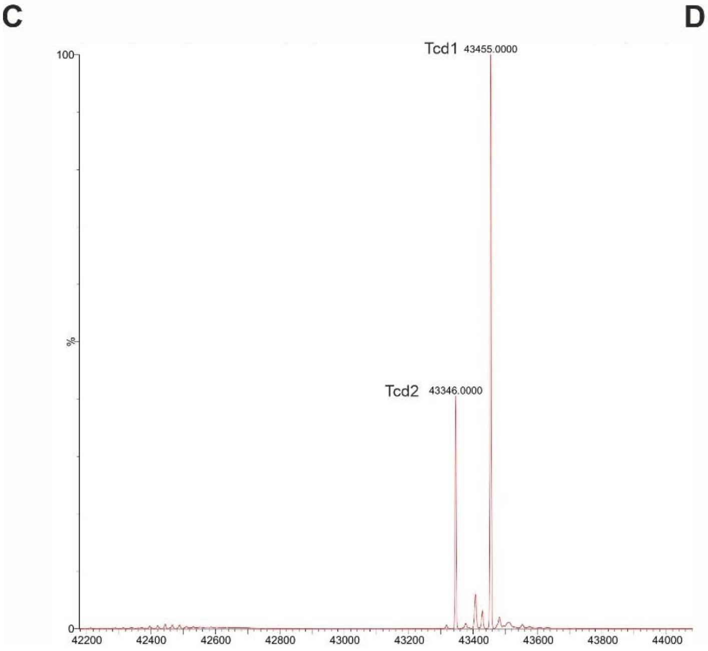

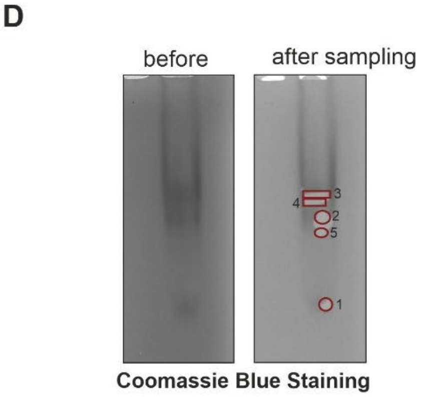

Sample	Hit 1	Hit 2
1	*S. cerevisiae* Tcd2	*S. cerevisiae* Tcd1
2	*S. cerevisiae* Tcd2	*S. cerevisiae* Tcd1
3	*S. cerevisiae* Tcd1	*S. cerevisiae* Tcd2
4	*S. cerevisiae* Tcd2	*S. cerevisiae* Tcd1
5	*S. cerevisiae* Tcd2	*S. cerevisiae* Tcd1

Supplementary Figure 12: Protein purification of the *S. cerevisiae* Tcd1$^{50\text{-}429}$-Tcd2$^{62\text{-}447}$-tRNA complex with only Tcd2 being tagged. See also Figure 5.
(A) Superdex 200 pg 16/600 size exclusion chromatography profile after tag cleavage. Fractions 1-7 are indicated. The same panel is also shown in Figure 5C. M_r values were calculated from the calibration curve in Supplementary Figure 28B. Earlier

chromatographic steps in the purification process are shown in Figure 5A, B and D. **(B)** Native PAGE of peak fractions 1-7 from panel A after tag cleavage; Tcd1-Tcd2-tRNA complex as purified in Supplementary Figure 11 was used as a positive control (+). The staining with SYBR Gold and Coomassie Blue reveals bands at the same height, suggesting tRNA binding. **(C)** ESI-MS analysis of pooled SEC fractions (1-7, see panel A) confirms the presence of both Tcd2$^{62\text{-}447}$ and Tcd1$^{50\text{-}429}$. Calculated molecular weight for Tcd1$^{50\text{-}429}$: 43,455 Da; and Tcd2$^{62\text{-}447}$: 43,346 Da. **(D)** Since samples 1-7 yielded multiple bands on native PAGE (panel B), additional peptide mass fingerprinting was performed. A sample of pooled SEC fractions (1-7, see panel A) was separated by native PAGE and stained with Coomassie Blue (left). All visible spots were stamped out (right) and analyzed. Notably, all samples contained both Tcd1 and Tcd2 (see Table).

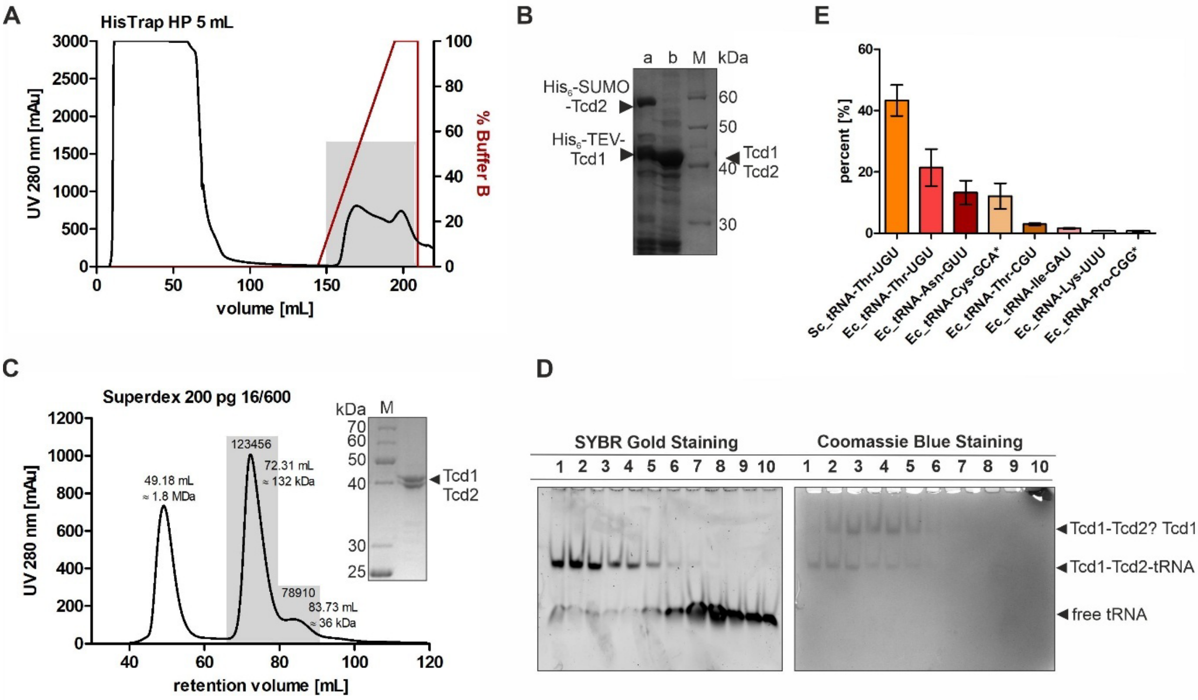

Supplementary Figure 13: Purification of the *S. cerevisiae* Tcd1$^{50\text{-}429}$-Tcd2$^{62\text{-}447}$-tRNA complex after co-expression with *S. cerevisiae* tRNA$^{Thr(UGU)}$ in *E. coli*.

(A) Ni affinity chromatography with pooled fractions shaded in gray. **(B)** SDS-PAGE (12%) confirmed the presence of His$_6$-SUMO-Tcd2 (56.8 kDa) and His$_6$-TEV-Tcd1 (45.5 kDa) in the pooled fractions from panel A (lane a). After proteolytic digest, both proteins migrate as a single band at about 40 kDa (Tcd1: 43.5 kDa, Tcd2: 43.3 kDa) (lane b). **(C)** Size exclusion profile with M_r values calculated from the calibration curve in Supplementary Figure 28B. Pooled peak fractions were analyzed by SDS-PAGE (12%), showing a band for Tcd1 and Tcd2 at 43 kDa. **(D)** Native PAGE with peak fractions from panel C. Lanes 1 to 6 show bands at the same height in SYBR Gold and Coomassie Blue staining, indicating tRNA binding. Additional bands for protein and tRNA only may arise from disrupted protein-tRNA complex. Lanes 7 to 10 show only free tRNA in the SYBR Gold staining. **(E)** Next-generation sequencing of co-purified tRNAs. The most frequently detected tRNA, observed with an incidence of nearly 45%, was tRNA$^{Thr(UGU)}$ from *S. cerevisiae*. *E. coli* tRNA$^{Thr(UGU)}$ was present at 25%. Little amounts of *E. coli* tRNA$^{Asn(GUU)}$ were also detected. tRNAs with codons other than NNU are most likely non-specifically bound and marked with an asterisk. Shown is the mean value from two independent purifications (one of which is shown in panels A-C) ± SEM.

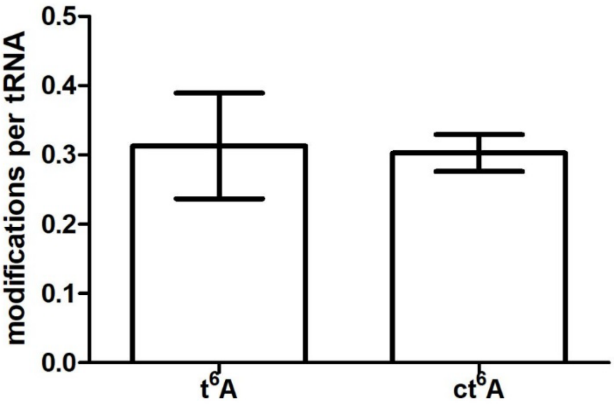

Supplementary Figure 14: t^{6}A and ct^{6}A contents in *S. cerevisiae* Tcd1$^{50\text{-}429}$-Tcd2$^{62\text{-}447}$-tRNA complex samples purified from *E. coli*.
Protein-tRNA complex samples were analyzed for their t^{6}A and ct^{6}A content by HLPC-MS. Amounts are given as modifications per tRNA. t^{6}A and ct^{6}A were equally present: About one out of 3.2 tRNAs contained t^{6}A and one out of 3.2 tRNAs contained ct^{6}A. 1.2 tRNAs appeared to contain none of the two modifications. Shown is the mean of three extractions ± SEM.

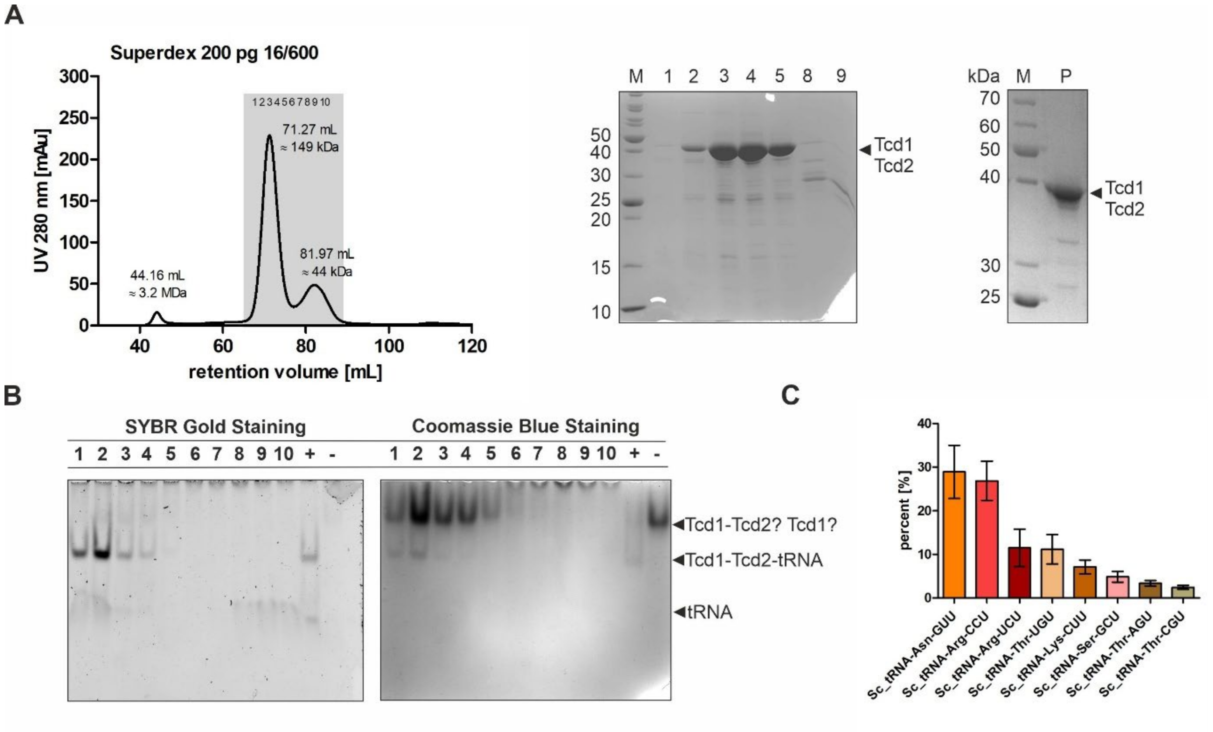

Supplementary Figure 15: Characterization of the native Tcd1$^{50-429}$-Tcd2$^{62-447}$-tRNA complex purified from *S. cerevisiae*.

(A) Superdex 200 pg 16/600 size exclusion chromatography profile (left), with M_r values calculated from the calibration curve in Supplementary Figure 28B, and SDS-PAGEs (middle and right) of selected SEC fractions. The band at ~40 kDa corresponds to the molecular weight of Tcd1 (43.5 kDa) and Tcd2 (43.4 kDa). Pooled fractions (P) from a second independent purification were resolved on a 12% SDS-PAGE (right) at lower concentration (right). **(B)** Native PAGE of the peak fractions 1-10 shown in panel A, a positive control (Tcd1-Tcd2-tRNA complex purified from *E. coli*; +) and a negative control (Tcd1; -). The staining with SYBR Gold and Coomassie Blue reveals bands at the same height, suggesting tRNA binding. **(C)** Next-generation sequencing of the co-purified tRNAs revealed that tRNA$^{Asn(GUU)}$ and tRNA$^{Arg(CCU)}$ had the highest occurrence rate, with a frequency of almost 30% each. tRNA$^{Arg(UCU)}$, tRNA$^{Thr(UGU)}$, tRNA$^{Lys(CUU)}$, tRNA$^{Ser(GCU)}$, tRNA$^{Thr(AGU)}$ and tRNA$^{Thr(CGU)}$ were also detected but in smaller quantities. Shown is the mean value from two independent biological samples/purifications and measurements ± SEM.

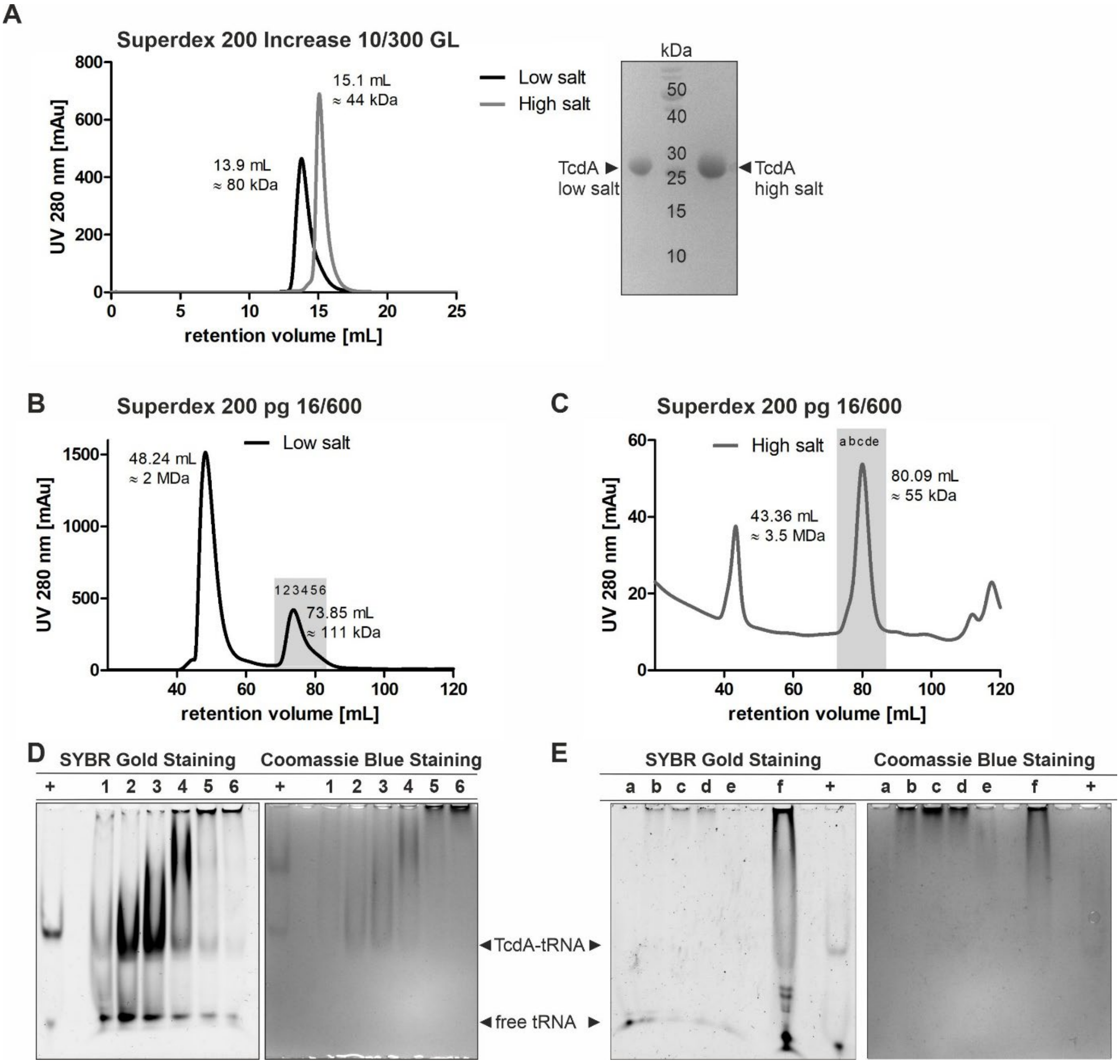

Supplementary Figure 16: Characterization of full-length *E. coli* TcdA.
(A-C) Size exclusion profiles of full-length *E. coli* TcdA (28.6 kDa) purified under low (black, 150 mM NaCl) and high (gray, 300 mM NaCl) salt conditions. Samples from preparative size exclusion chromatography (Superdex 200 pg 16/600, panels B and C) were additionally analyzed on an analytical column (Superdex 200 Increase 10/300 GL, panel A). M_r values were calculated from the calibration curves in Supplementary Figure 28B and D. Note that the peak for TcdA purified under low salt conditions consists of several overlapping peaks/coeluting species as confirmed by native PAGE (panel D). **(D-E)** Native PAGEs of SEC fractions from panels B and C. Bands at the same height in SYBR Gold and Coomassie Blue staining of panel D indicate that TcdA co-purified with tRNA under low salt conditions. By contrast, under high salt conditions (panel E), no nucleic acid was co-purified. The Tcd1-Tcd2-tRNA complex served as a positive control (+), lane f in panel E represents TcdA (sample c) incubated with 750 ng of *in vitro* transcribed $tRNA^{Thr(AGU)}$.

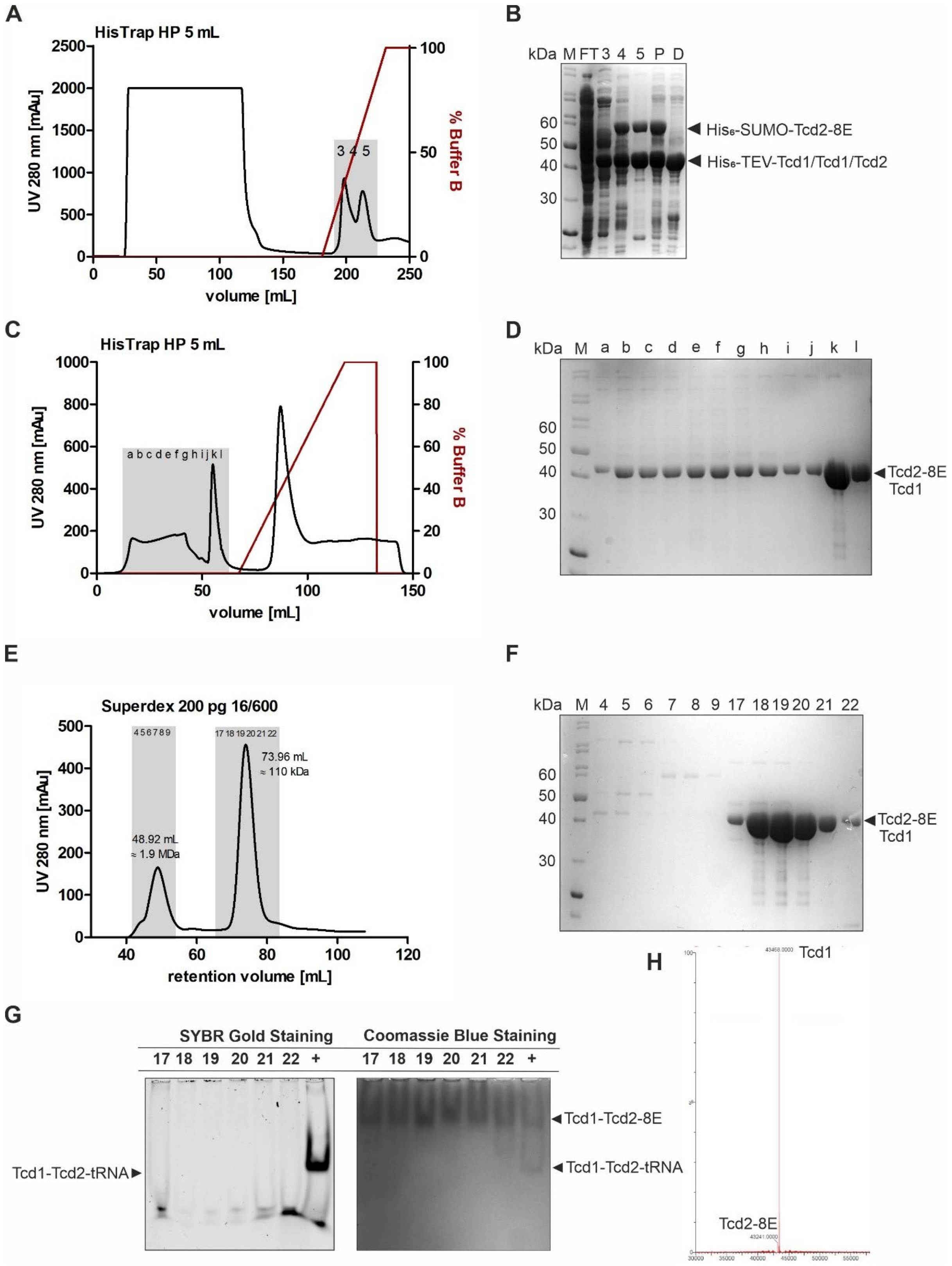

Supplementary Figure 17: Purification of *S. cerevisiae* Tcd1$^{50\text{-}429}$Tcd2$^{62\text{-}447}$-8E from *E. coli*.

(A) First Ni affinity chromatography. **(B)** 12% SDS-PAGE of selected fractions from panel A (FT = flow through, P = Pool of fractions 4 and 5). His$_6$-TEV-Tcd1$^{50\text{-}429}$ (45.5

kDa) and His_6-SUMO-Tcd2$^{62\text{-}447}$-8E (56.5 kDa) are clearly present in fractions 4 and 5. After tag removal by protease digestion (sample D), both Tcd1$^{50\text{-}429}$ (43.5 kDa) and Tcd2$^{62\text{-}447}$-8E (43.2 kDa) migrate as one band. **(C)** Reverse Ni affinity chromatography to separate cleaved from uncleaved protein, tags and proteases. **(D)** Fractions of the flow through from panel C were analyzed on a 12% SDS-PAGE. **(E)** Size exclusion chromatography (Superdex 200 pg 16/600) indicates an aggregate peak (~49 mL) that seems to mainly consist of aggregated tRNA (no prominent protein bands were detectable by SDS-PAGE, see panel F), and the target peak at ~74 mL. M_r values were calculated from the calibration curve in Supplementary Figure 28B. **(F)** 12% SDS-PAGE of selected fractions from panel E. **(G)** Native PAGE with selected fractions from the size exclusion chromatography shown in panel E. The nucleic acid staining on the left indicates no tRNA binding in the SEC fractions 17-22. Purified WT Tcd1-Tcd2-tRNA complex served as a positive control (+). **(H)** A sample of the pooled fractions 17-22 was analyzed by ESI-MS. Both Tcd1$^{50\text{-}429}$ and Tcd2$^{62\text{-}447}$-8E could be identified (expected molecular weight for Tcd1$^{50\text{-}429}$: 43,468 Da; for Tcd2$^{62\text{-}447}$-8E: 43,241 Da), suggesting that Tcd1$^{50\text{-}429}$ and Tcd2$^{62\text{-}447}$-8E form a heterocomplex that fails to bind tRNA.

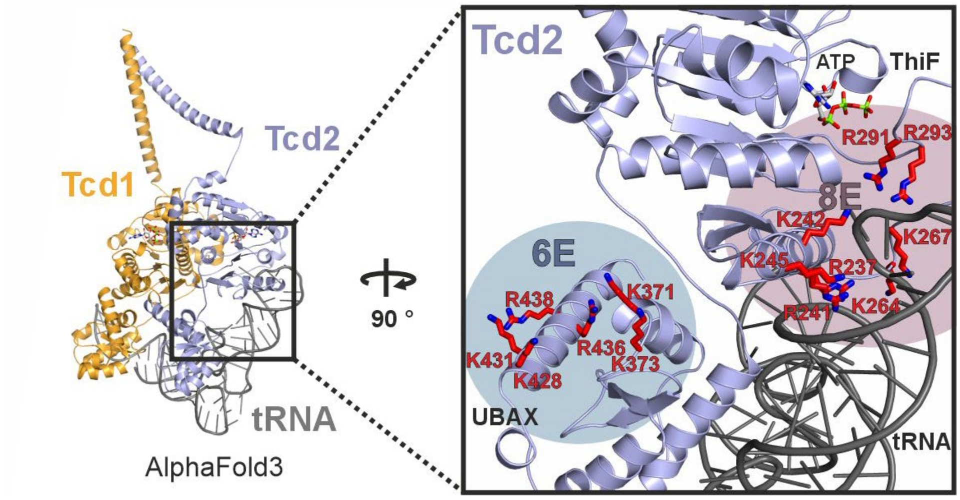

Supplementary Figure 18: Mapping sites of mutation on *S. cerevisiae* Tcd2.
AlphaFold 3 (5) prediction of the *S. cerevisiae* Tcd1-Tcd2-tRNA complex (left) with zoom-in picture on Tcd2 (right). Two patches (shaded in red and blue, respectively) of in total 14 basic amino acids (lysines or arginines; red sticks) are present in Tcd2. Simultaneous mutation of the eight basic residues clustered in the N-terminal ThiF domain of Tcd2 (red shaded zone) to glutamates (construct 8E), led to an RNA-free protein preparation (Supplementary Figure 17), suggesting that the 8E area is indeed relevant for tRNA binding.

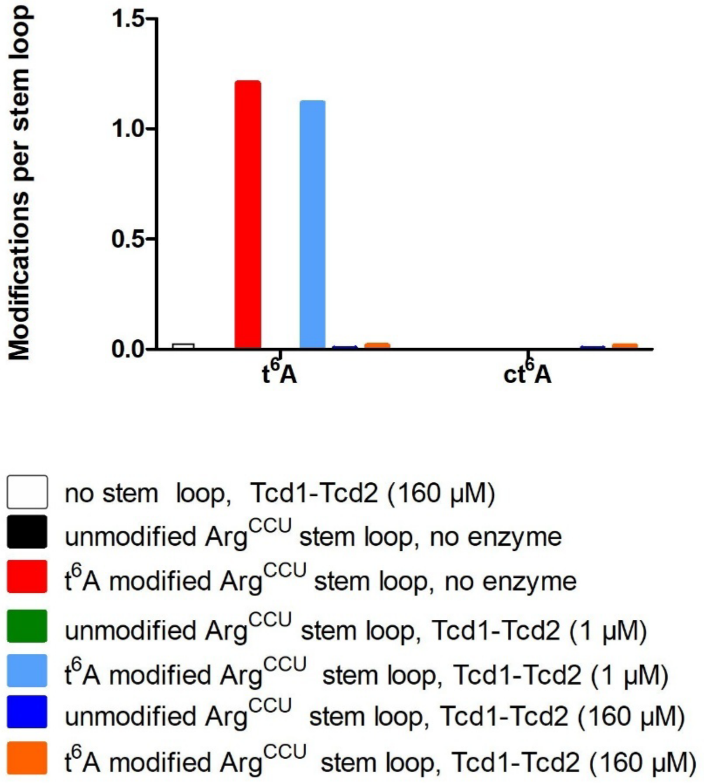

Supplementary Figure 19: *In vitro* activity assay for *S. cerevisiae* Tcd1$^{50\text{-}429}$-Tcd2$^{62\text{-}447}$ with chemically synthesized, biotinylated tRNA stem loops. Chemically synthesized, biotinylated, unmodified or t^{6}A-modified tRNA$^{Arg(CCU)}$ stem loop (16 µM; for sequences see Supplementary Table 3) was incubated at 22°C for 16 h with *S. cerevisiae* Tcd1$^{50\text{-}429}$-Tcd2$^{62\text{-}447}$ complex (0, 1, 160 µM) purified from *E. coli*. Because the Tcd1-Tcd2 preparations always contain tRNA co-purified from *E. coli*, the synthetic stem loops were afterwards extracted using the biotin label, hydrolyzed and quantified by HPLC-MS. No ct^{6}A was detectable. While the t^{6}A modification of the substrate was detectable after incubation with 1 µM enzyme complex, the signal completely vanished after incubation with 160 µM Tcd1-Tcd2-tRNA heterocomplex. A possible explanation was co-precipitation of the enzyme and with the stem loops during purification. The assay was not further optimized. Instead, the *in vivo* set-up was used.

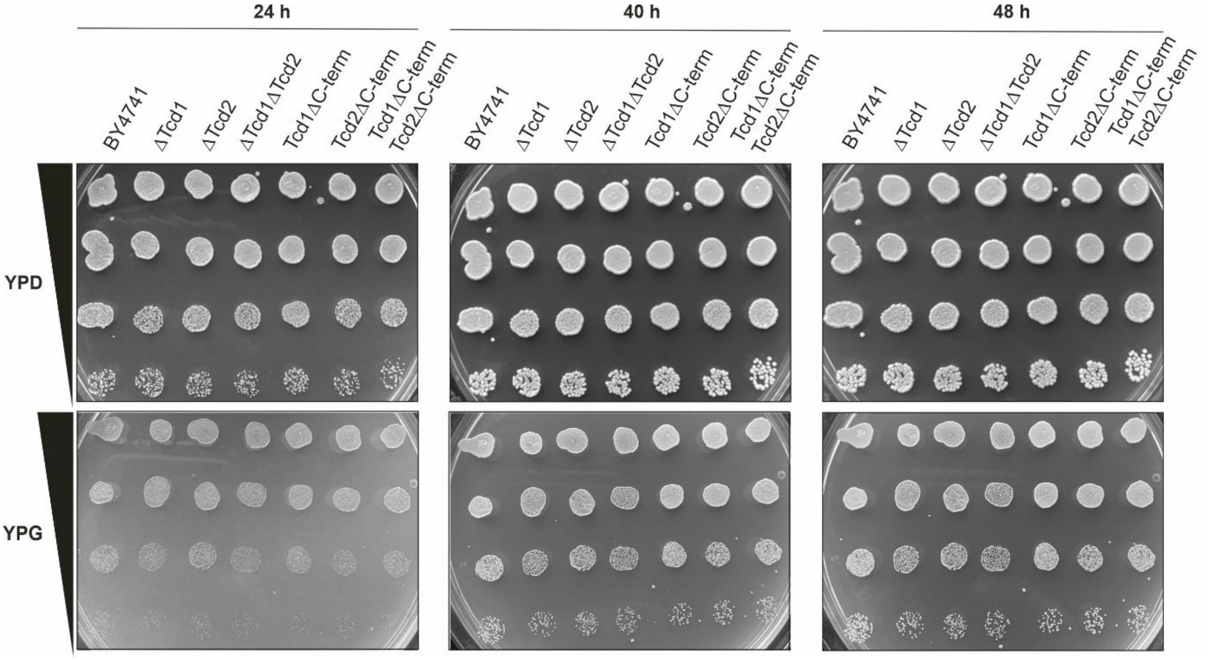

Supplementary Figure 20: Droptest of *S. cerevisiae* knockout strains.Serial dilutions of yeast cells were spotted on YPD (Yeast Peptone Dextrose) and YPG (Yeast Peptone Glycerol) plates and grown for 2 days at 30°C.

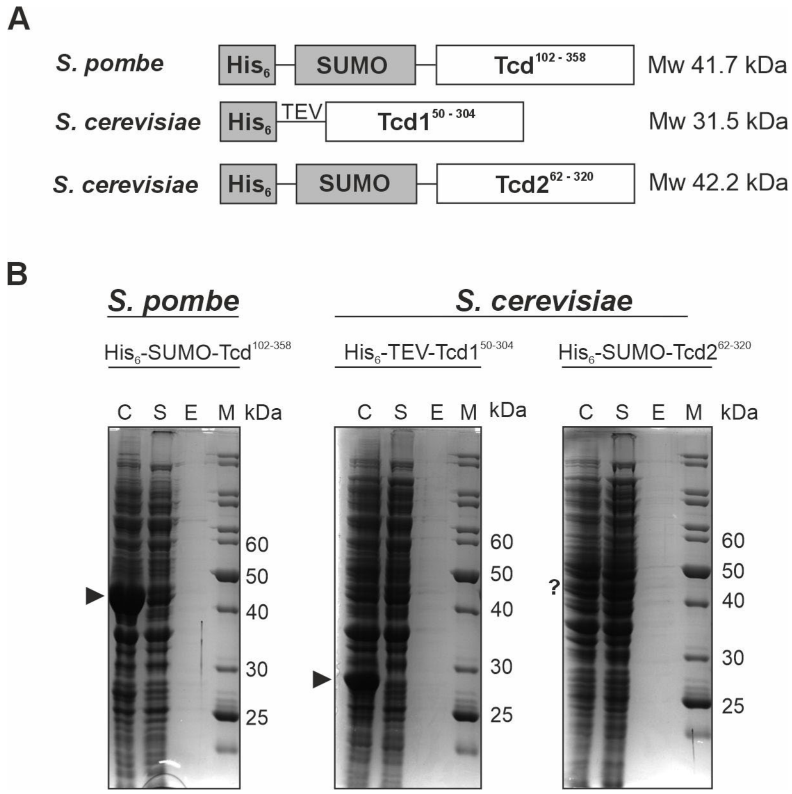

Supplementary Figure 21: The isolated N-terminal ThiF domains of yeast Tcd enzymes are not accessible to purification under the chosen conditions.
(A) Schematic illustration of the expression constructs used to purify yeast Tcd enzymes without their C-terminal domain and their corresponding molecular weights. **(B)** 12% SDS-PAGE of selected fractions from test expression experiments at 20°C and quick Ni affinity purification with single-use Ni-NTA columns: whole cells (C), supernatant after cell disruption (S), elution fraction (E). For *S. pombe* Tcd and *S. cerevisiae* Tcd1 insoluble protein production was noted, whereas *S. cerevisiae* Tcd2 was either not produced or instable and degraded, as no significant protein band at the expected size was detectable under the chosen conditions.

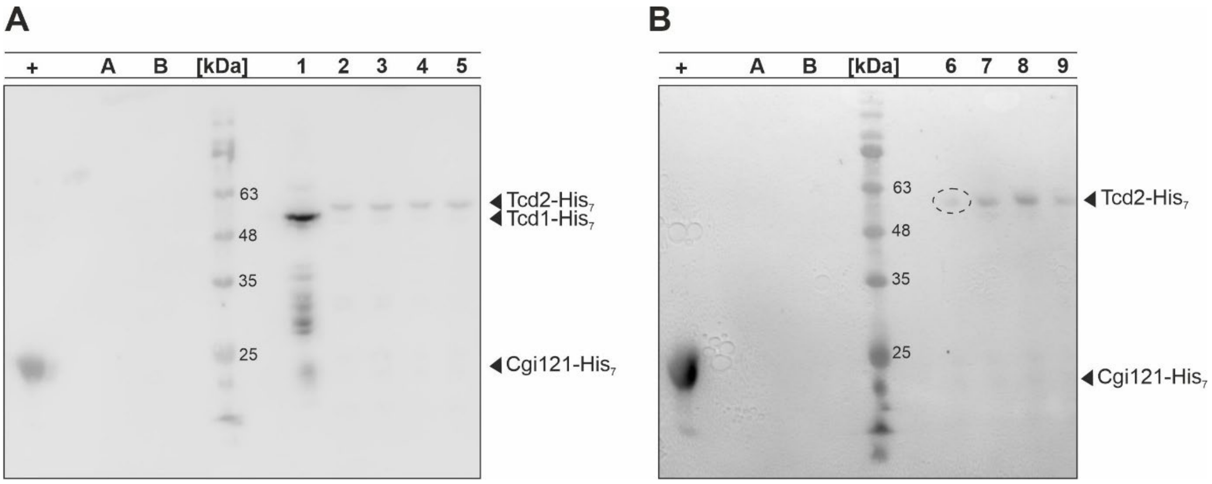

Supplementary Figure 22: α-His Western blots of solubilized mitochondria isolated from diverse *S. cerevisiae* strains.

Mitochondria were isolated from various *S. cerevisiae* strains and probed by Western blotting with an α-His antibody (Supplementary Table 14) for the production of mutant C-terminally His_7-tagged Tcd1 or Tcd2 versions.

Lane +: purified His_7-tagged protein Cgi121 – that is part of the KEOPS complex involved in t^6A formation – served as a positive control; lanes A (WT *S. cerevisiae* BY4741) and B (*Δtcd1Δtcd2*) served as negative controls; lanes 1-9: Yeast strain *Δtcd1Δtcd2* transformed with pBEVY-GL plasmids coding for C-terminally His_7-tagged Tcd1 or 2 variants (lane 1: $Tcd1_{R59A}$, lane 2: $Tcd2_{R128A}$, lane 3: $Tcd2_{K141A}$, lane 4: $Tcd2_{D188A}$, lane 5: $Tcd2_{S285A}$, lane 6: $Tcd2_{R291A}$, lane 7: $Tcd2_{R291E}$, lane 8: $Tcd2_{R293E}$, and lane 9: $Tcd2_{L295A}$; see also Supplementary Table 11).

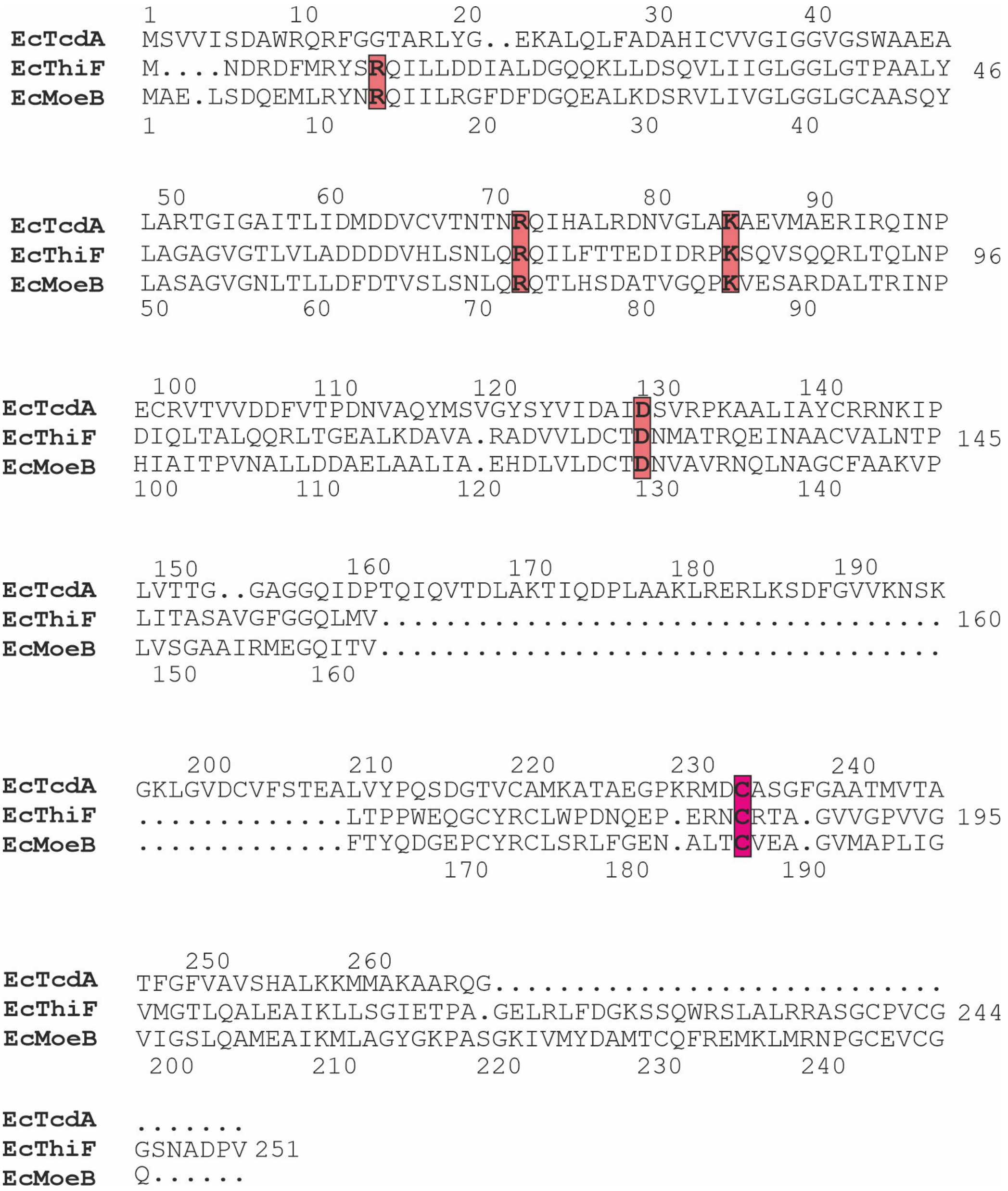

Supplementary Figure 23: Sequence alignment of *E. coli* TcdA, ThiF and MoeB. The catalytic cysteine of ThiF, Cys184 (pink), required for formation of a covalent acyl disulfide linkage (6,7), is conserved in TcdA and MoeB, although it probably has no function in the latter two proteins. For *E. coli* TcdA, we here propose a crucial catalytic function of Asp130 (red) which is also conserved in ThiF, MoeB and yeast Tcds (Supplementary Figure 1). The equivalent of Asp130 in Tcd2, Asp188, is essential for ct^{6}A formation (Supplementary Figure 1 and Figure 7C).

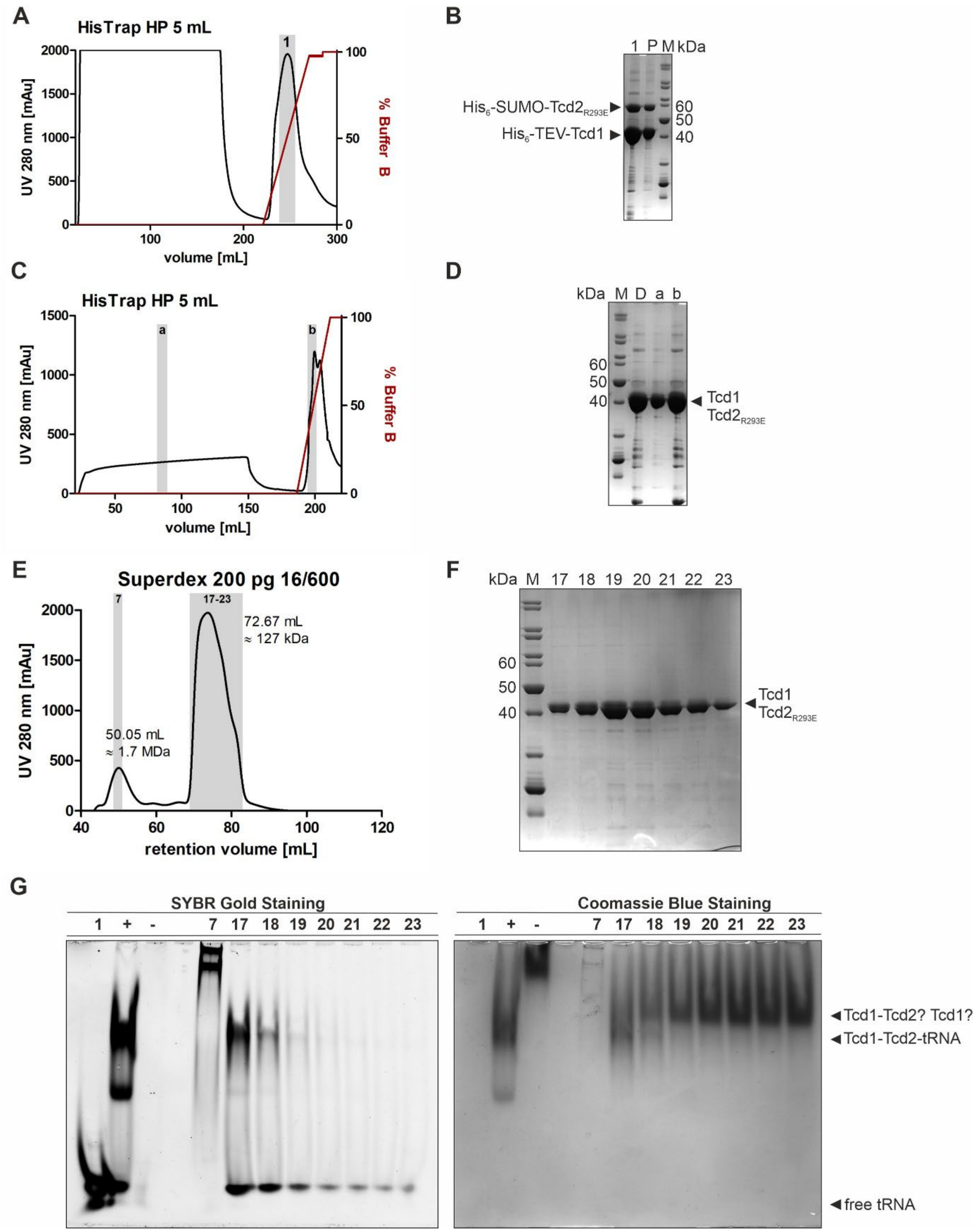

Supplementary Figure 24: Purification of *S. cerevisiae* Tcd1$^{50\text{-}429}$Tcd2$^{62\text{-}447}$-R293E from *E. coli*.

(A) First Ni affinity chromatography. **(B)** 12% SDS-PAGE of fraction 1 from Ni affinity chromatography shown in panel A. His$_6$-TEV-Tcd1 (45.5 kDa) and His$_6$-SUMO-Tcd2-R293E (56.5 kDa) are clearly present in fraction 1. Sample P denotes precipitate that arose in fraction 1 upon short-term storage. **(C)** Second Ni affinity chromatography to

separate cleaved from uncleaved protein, tags and proteases after the addition of TEV and SUMO proteases. **(D)** Fractions of the flow through and elution from panel C were separated on a 12% SDS-PAGE. Upon tag removal Tcd1 (43.5 kDa) and Tcd2-R293E (43.3 kDa) migrate as a single band at about 40 kDa. **(E)** Size exclusion chromatography profile (Superdex 200 pg 16/600) with M_r values calculated from the calibration curve in Supplementary Figure 28B. **(F)** 12% SDS-PAGE of selected fractions from panel E. **(G)** Native PAGE with selected fractions from panel E. Free tRNA (yeast tRNA mix, lane 1) and purified WT Tcd1-Tcd2-tRNA complex (lane +) served as positive controls, while yeast Sua5, a protein that is involved in t^6A formation but does not bind nucleic acids, was used as a negative control (-). The nucleic acid staining on the left indicates tRNA binding in the SEC fractions 17-19, implying that the mutation R293E does not prevent tRNA binding.

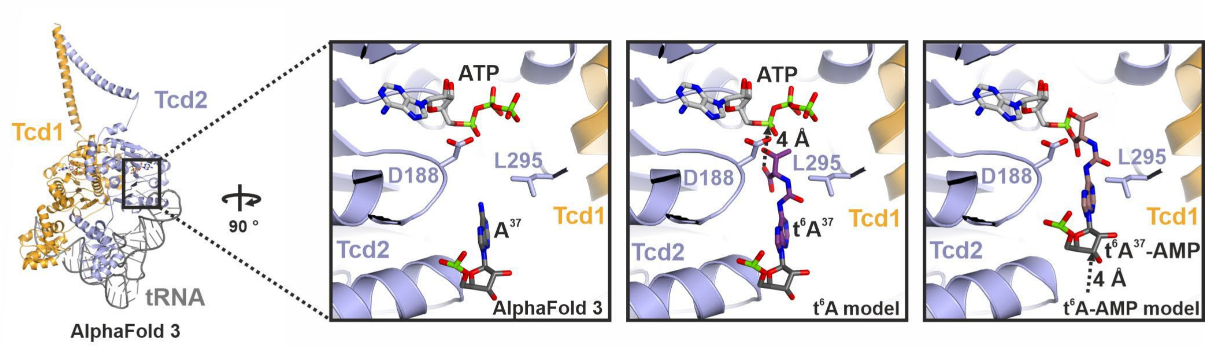

Supplementary Figure 25: Catalytic model for the conversion of t^6A to t^6A-AMP in Tcd enzymes.

Based on the AlphaFold 3 prediction of the *S. cerevisiae* Tcd1-Tcd2-tRNA complex (cartoon image on the very left), adenosine 37 in the active site of Tcd2 (leftmost framed panel) was extended to a t^6A moiety (framed middle panel). The t^6A moiety was built in Sybyl 8.0 (8) and minimized by 100 steps of Powell's method. Coot (9) was used to generate restraints (prodrg-ify this residue tool) and to fit the ligand to the AlphaFold 3 model of the Tcd1-Tcd2-tRNA complex. The conformation of t^6A was chosen such that adenylation and cyclization are feasible. However, it appeared that the distance of A37 (modelled by AlphaFold 3) and ATP is too far for adenylation of t^6A (black dotted arrow in framed middle panel). In fact, the t^6A moiety has to move about 4 Å deeper into the active site pocket either before or during the formation of t^6A-AMP (rightmost framed panel; black dotted arrow). After adenylation, cyclization of t^6A to ct^6A is conceivable without the help of any catalytic residue (see also Supplementary Figure 29E and Figure 9B).

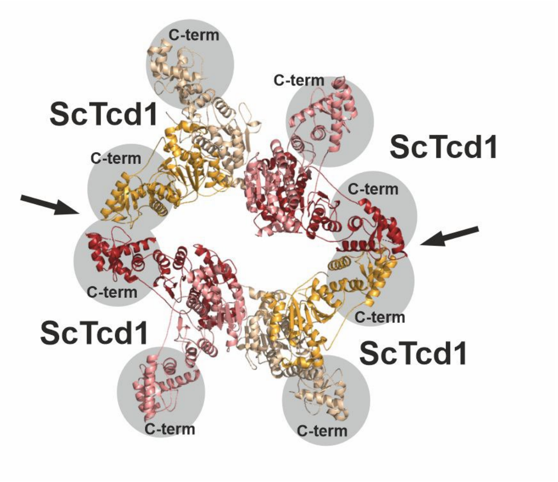

Supplementary Figure 26: The C-terminal domain of *S. cerevisiae* Tcd1 forms intermolecular contacts in the crystal lattice.
Four ScTcd1 homodimers of the crystal lattice are illustrated as cartoon models in reddish and yellowish respectively. Their C-terminal domains (C-term) are highlighted against a gray background and labelled. Crystal contacts via the C-terminal domain might explain their bigger distance in the ScTcd1 homodimer compared to the AlphaFold 3 predictions shown in Figure 8B.

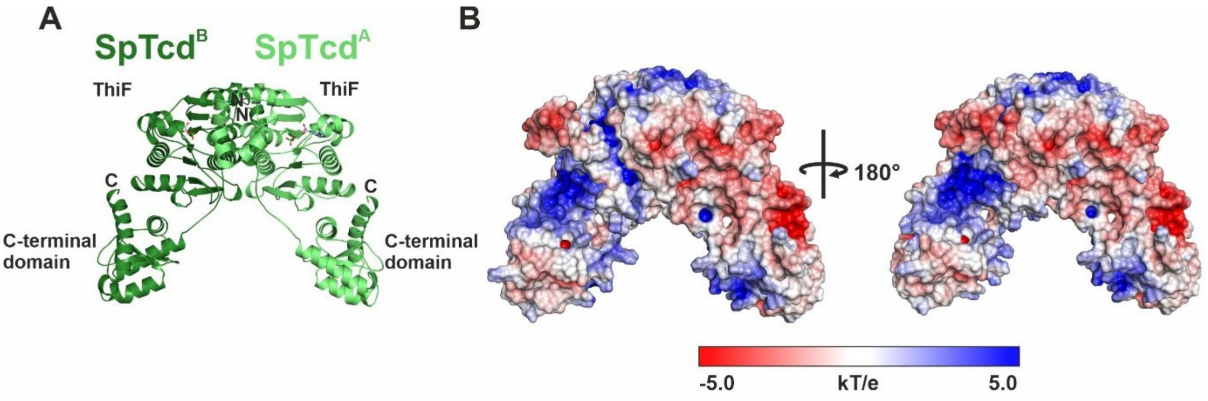

Supplementary Figure 27: Structural model of *S. pombe* Tcd.
(A) Ribbon illustration of the SpTcd homodimer. **(B)** Electrostatic surface illustrations of the SpTcd homodimer with colors ranging from -5.0 kT/e (red) to 5.0 kT/e (blue). Each SpTcd subunit harbors a characteristic positively charged patch, responsible for tRNA binding on opposite sides of the homodimer.

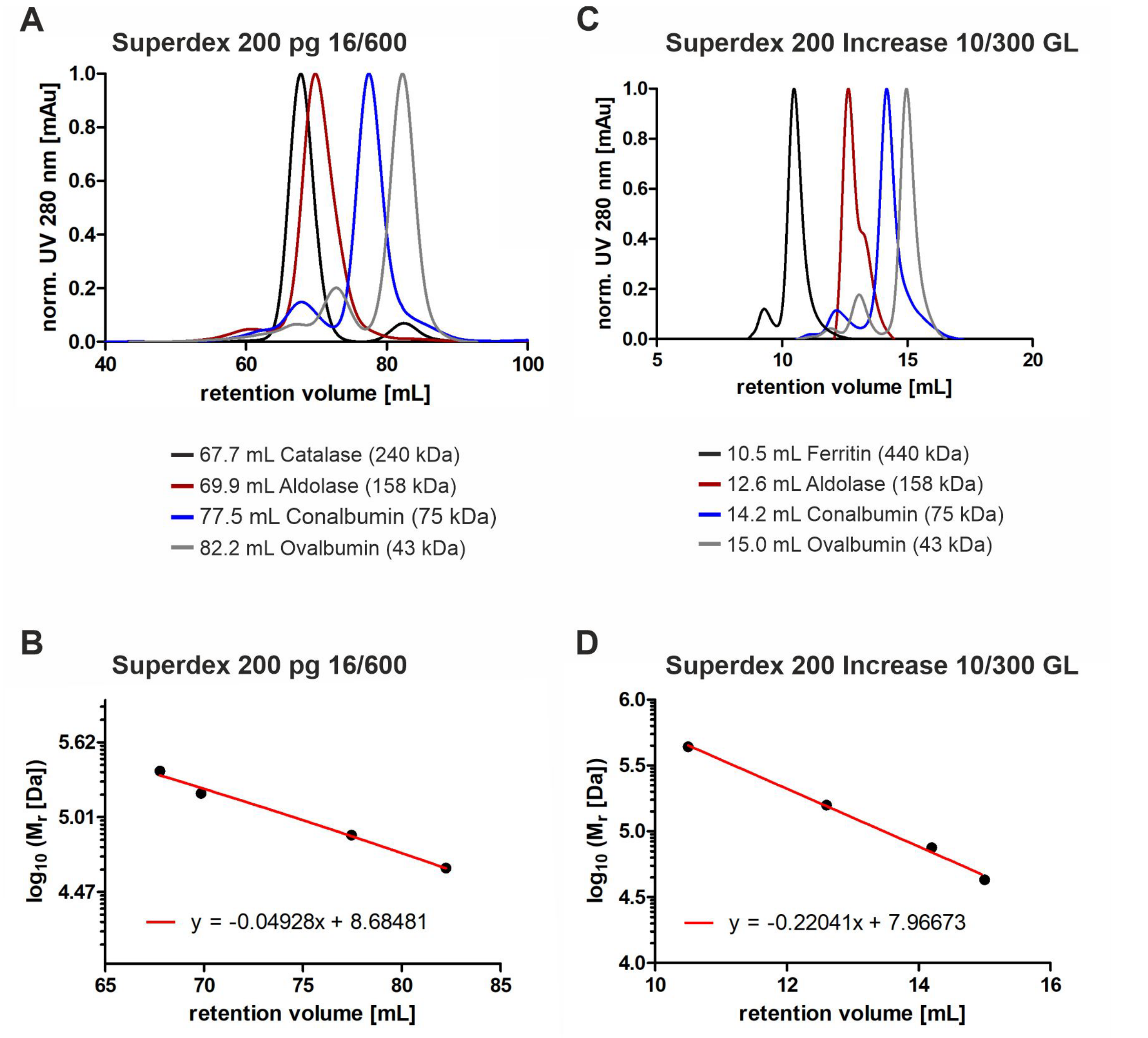

Supplementary Figure 28: Calibration of size exclusion chromatography columns.

(A, C) Elution profiles of marker proteins on (A) a Superdex 200 pg 16/600 column and (C) a Superdex 200 Increase 10/300 GL column.

(B, D) Retention volumes determined from panel A and C were plotted against the logarithm of the molecular weight of the corresponding proteins (black circles) and fitted. Equations for the linear regression line (red) are provided. Please note that size exclusion chromatography separates proteins based on their hydrodynamic radius, which is dependent on size and shape of the macromolecule and thus gives only a rough estimation of the molecular weight or oligomeric state.

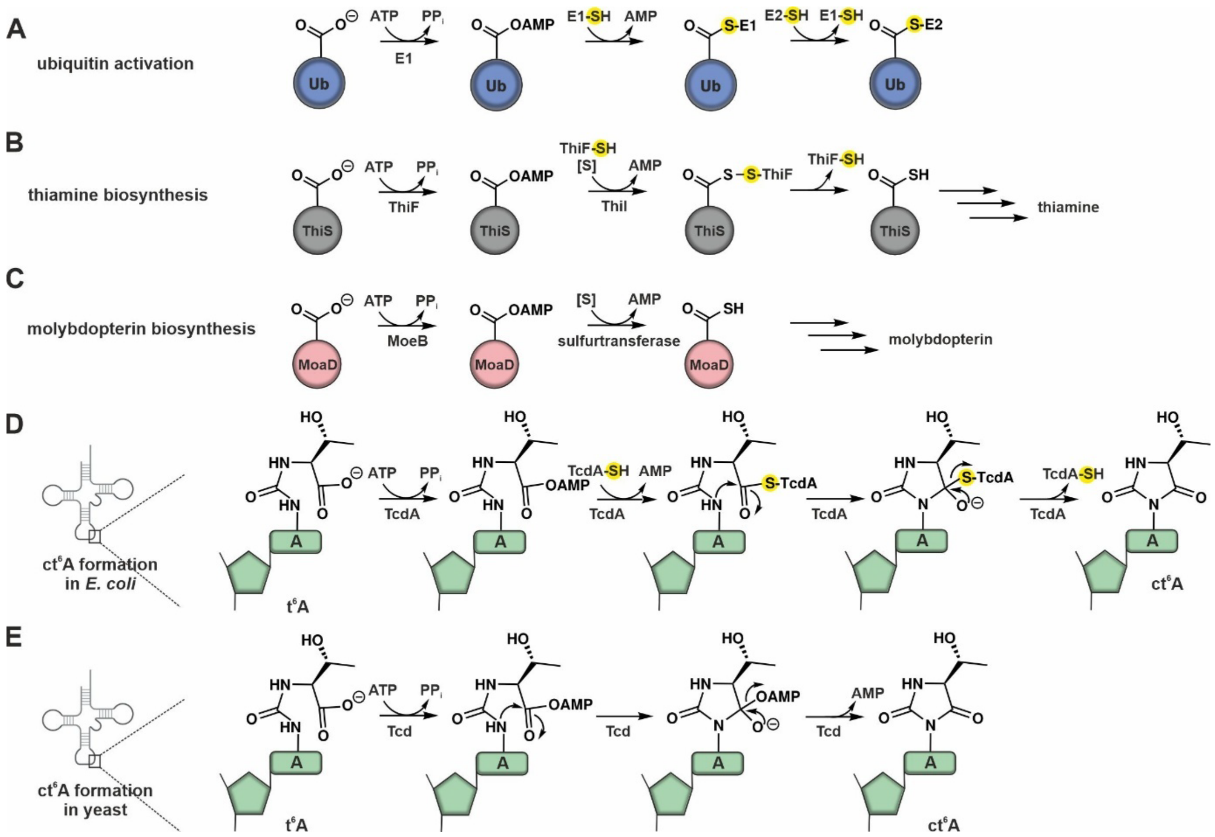

Supplementary Figure 29: Mechanistic comparison of ct^{6}A formation in yeast and bacteria as well as related modification systems.

(A) Mechanism of ubiquitin (Ub) activation by E1 enzymes and transfer onto E2 enzymes. E1 enzymes show structural and mechanistic similarities to ThiF (B), MoeB (C) and Tcd enzymes (D-E). **(B)** As part of the thiamine biosynthesis pathway in *E. coli*, ThiS is modified by the proteins ThiF and ThiI. This reaction sequence involves a covalent acyl disulfide linkage between ThiS and ThiF (6,7). **(C)** During molybdopterin biosynthesis MoaD is adenylated by MoeB but the formation of the reactive MoaD thiocarboxylate does not involve a covalent linkage with MoeB (10), which contrasts thiamine biosynthesis. **(D)** Tcd enzymes catalyze the ATP dependent cyclisation of t^{6}A to ct^{6}A on tRNA. For *E. coli* TcdA, a reaction mechanism involving a catalytic cysteine and leading to the oxazolone isoform of ct^{6}A has been proposed (11). Today, ct^{6}A is known to adopt the hydantoin isoform *in vivo* and the mechanistic proposal of Kim *et al.*, 2015 has been adapted accordingly here. Experimental proof for the importance of a catalytic TcdA cysteine is however lacking. **(E)** Yeast Tcd enzymes lack the catalytic cysteine of *E. coli* TcdA. We therefore propose that the enzymatic environment is sufficient to catalyze intramolecular cyclisation of the t^{6}A-AMP intermediate (Supplementary Figure 25 and Figure 9B).

The tRNA shown in panels D and E has been created in BioRender. Huber, E. (2026) https://BioRender.com/h55raqy.

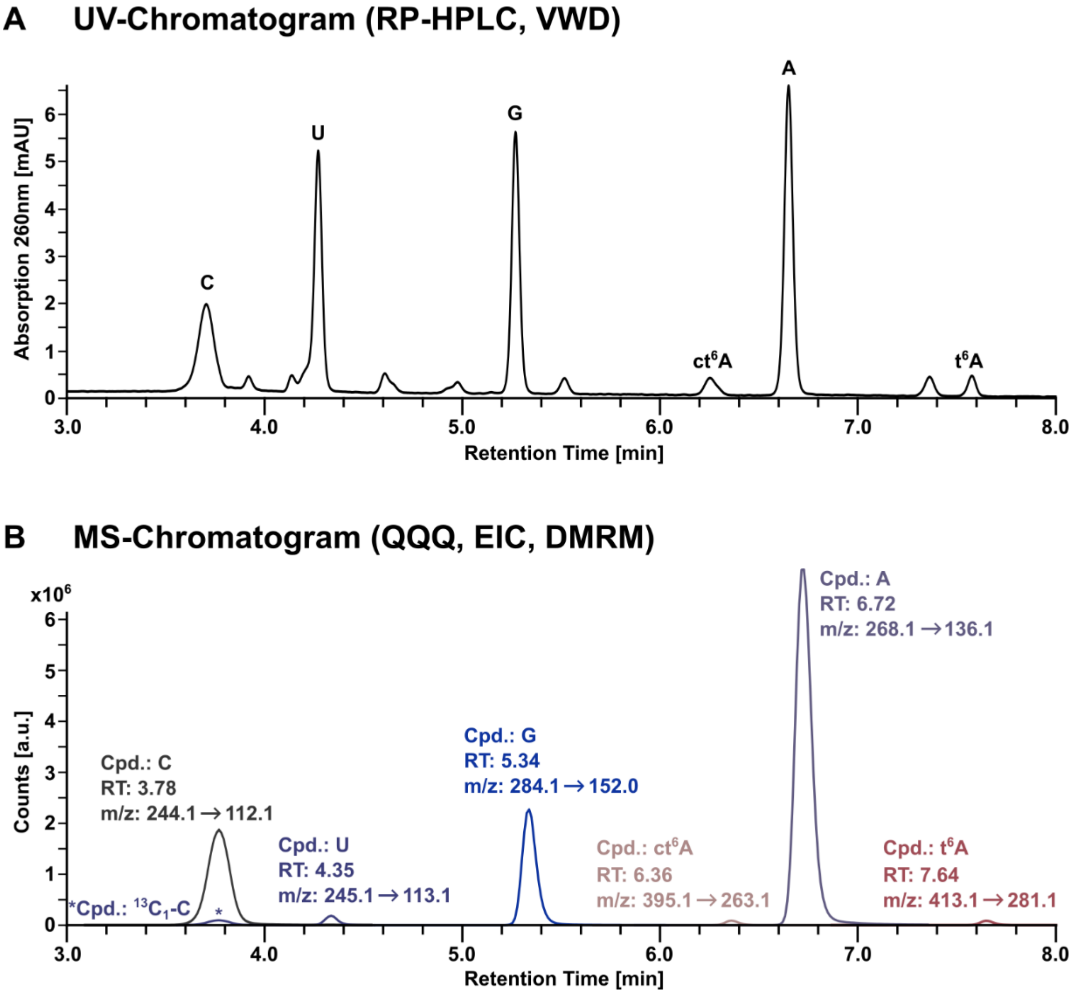

Supplementary Figure 30: Representative (A) UV- and (B) MS-chromatograms obtained from the same measurement corresponding to calibration level 10.
At this calibration level, 25 pmol of canonical nucleosides (C, U, G, A) and 1.25 pmol of the modifications (t^6A, ct^6A) were injected. The y-axis shows milli-absorbance units (mAU) for UV detection and ion counts (arbitrary units, a. u.) for MS detection. In the UV chromatogram additional peaks are visible, originating from other modified nucleosides present in the standardized calibration mix; these modifications were not analyzed in this study.

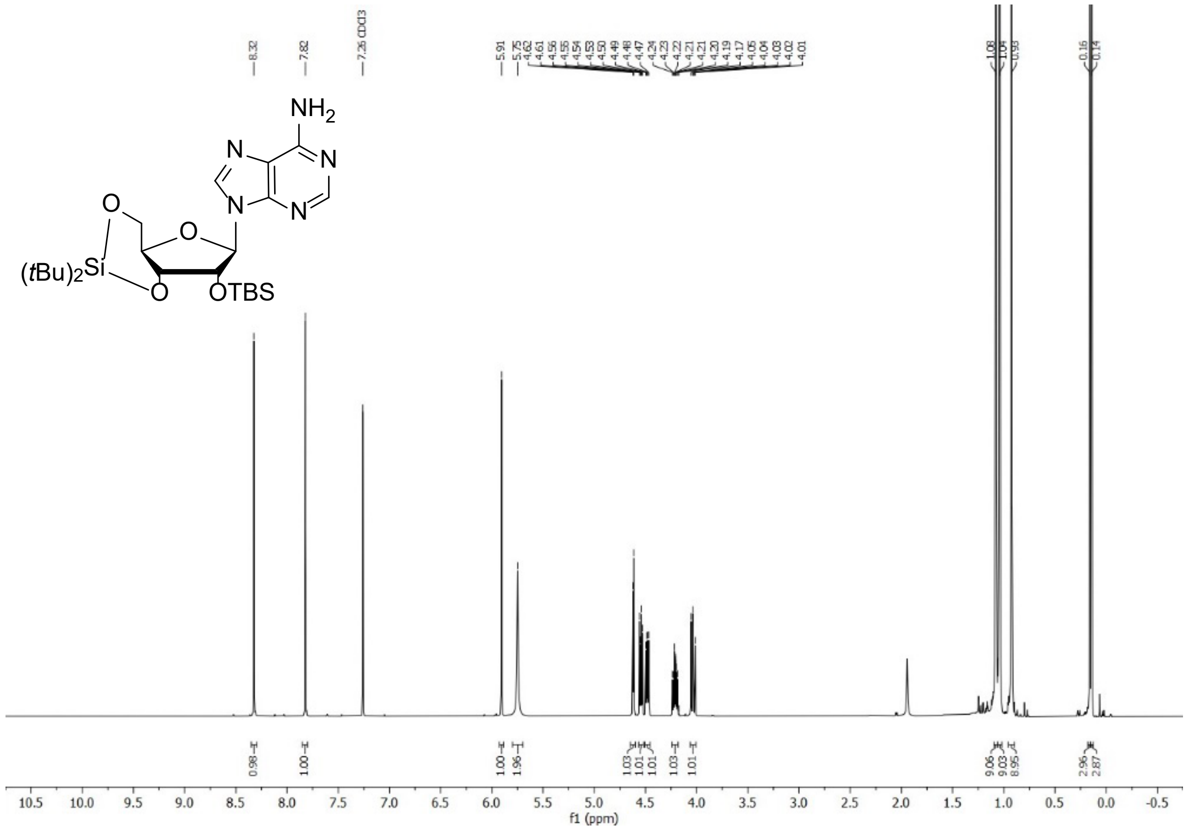

Supplementary Figure 31: ^{1}H-NMR spectrum of compound 2.

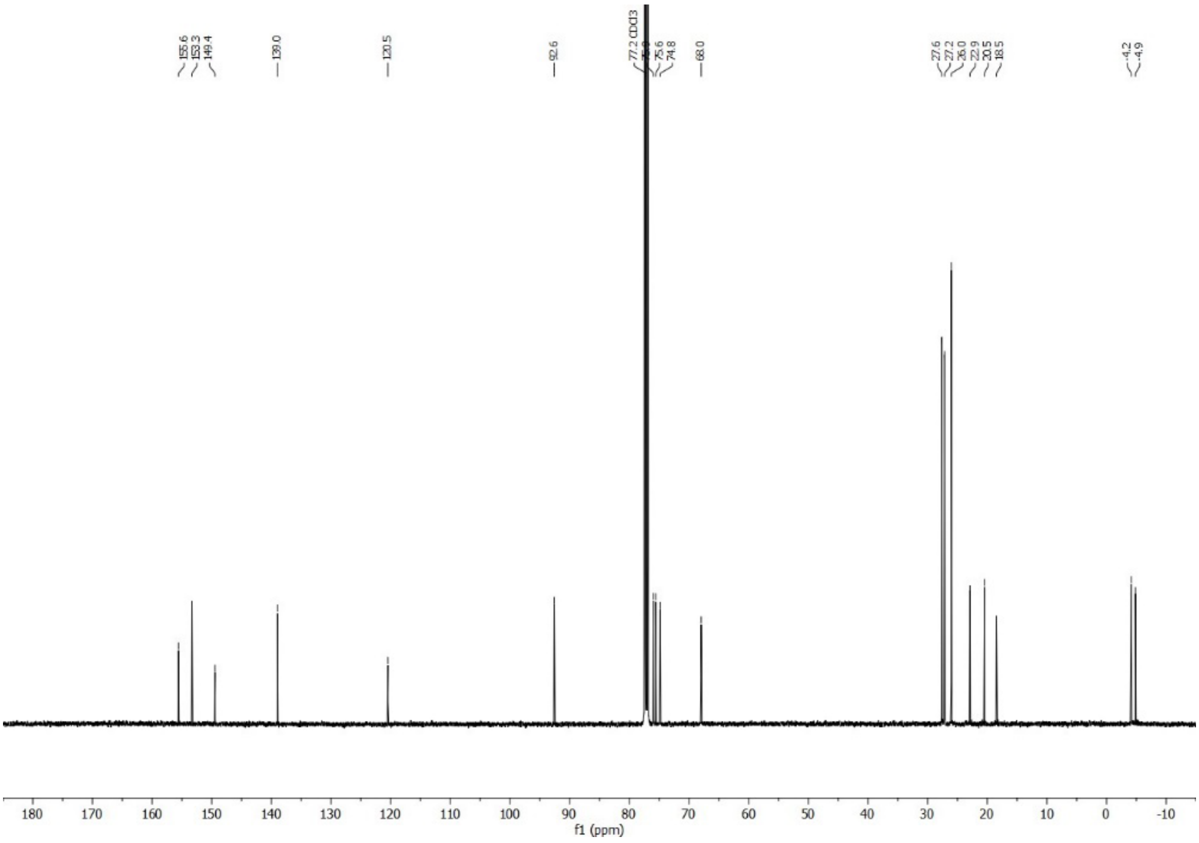

Supplementary Figure 32: ^{13}C-NMR spectrum of compound 2.

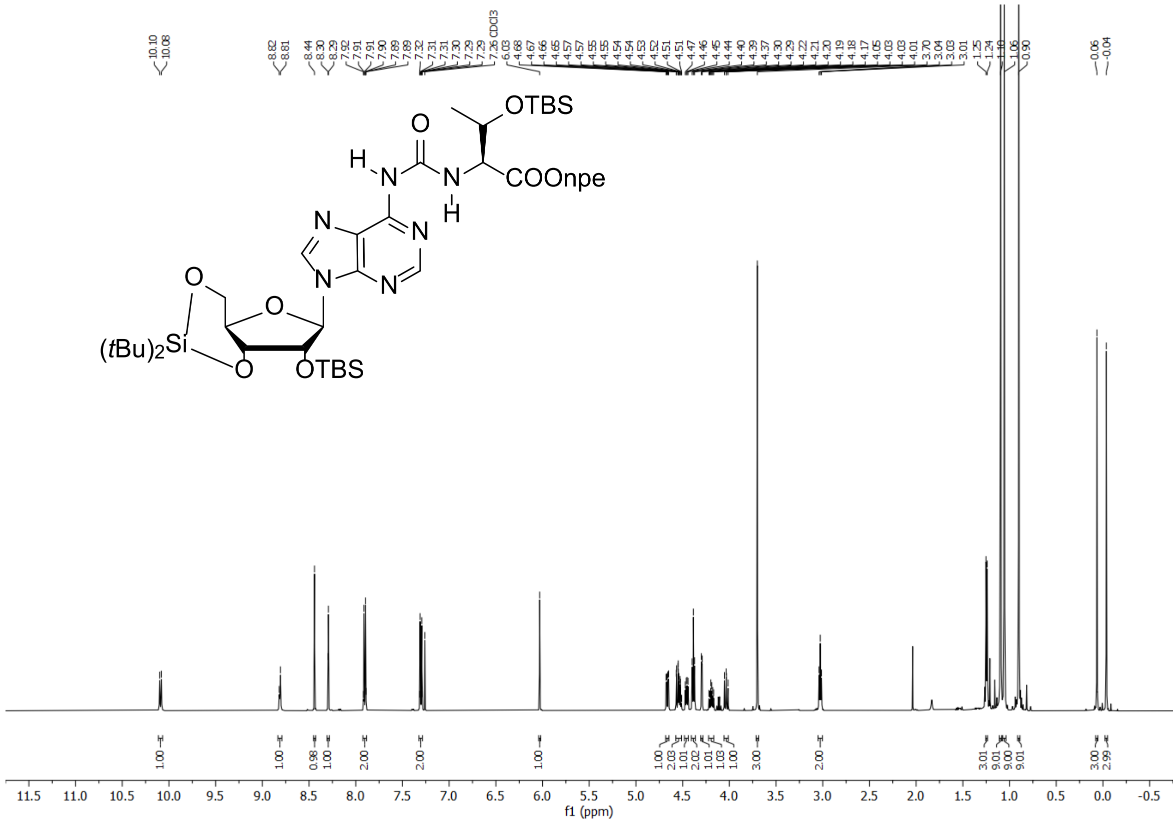

Supplementary Figure 33: ^{1}H-NMR spectrum of compound 3.

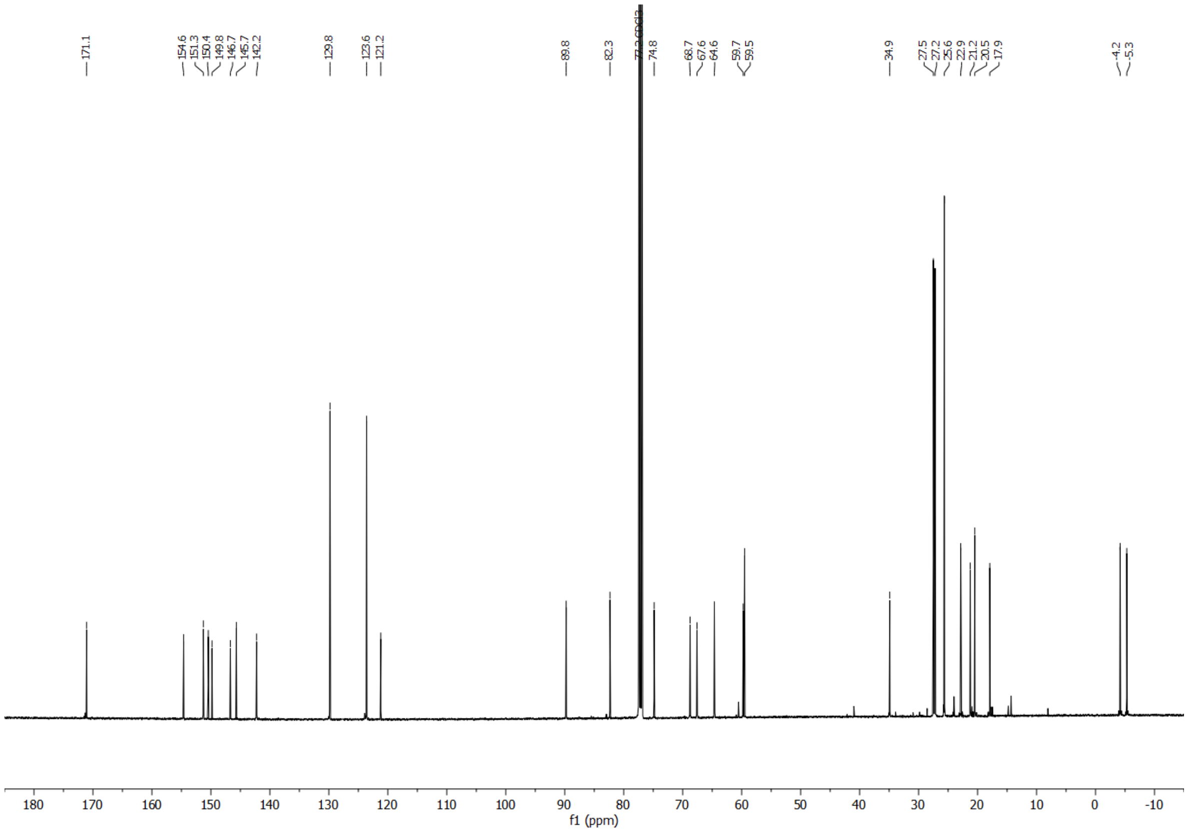

Supplementary Figure 34: ^{13}C-NMR spectrum of compound 3.

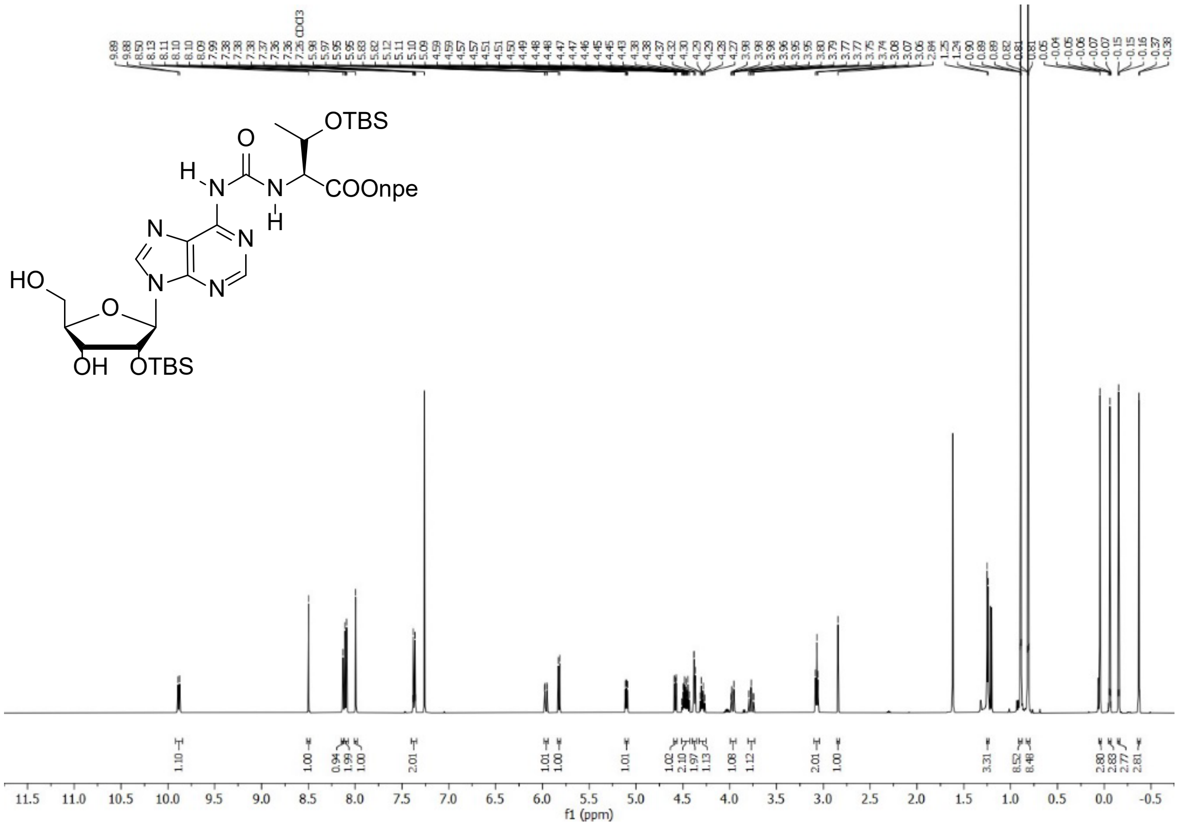

Supplementary Figure 35: ^{1}H-NMR spectrum of compound 4.

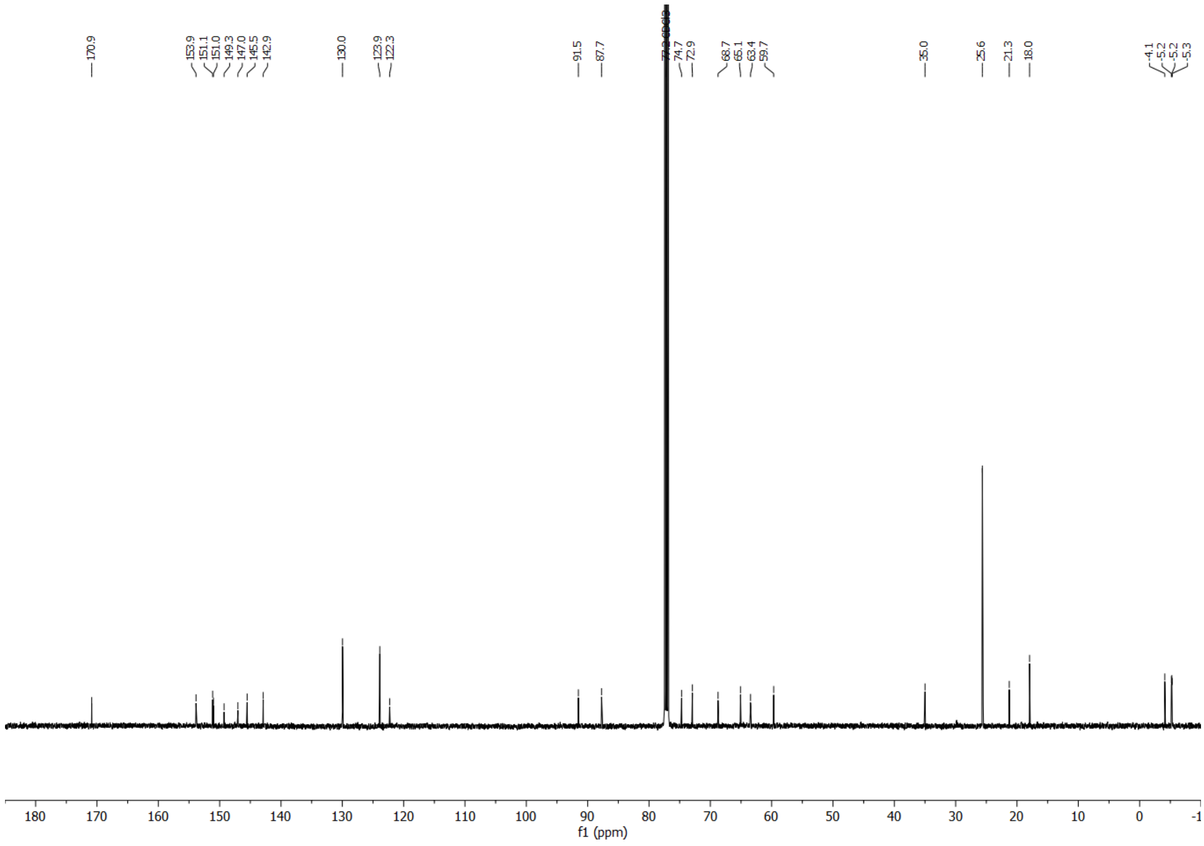

Supplementary Figure 36: ^{13}C-NMR spectrum of compound 4.

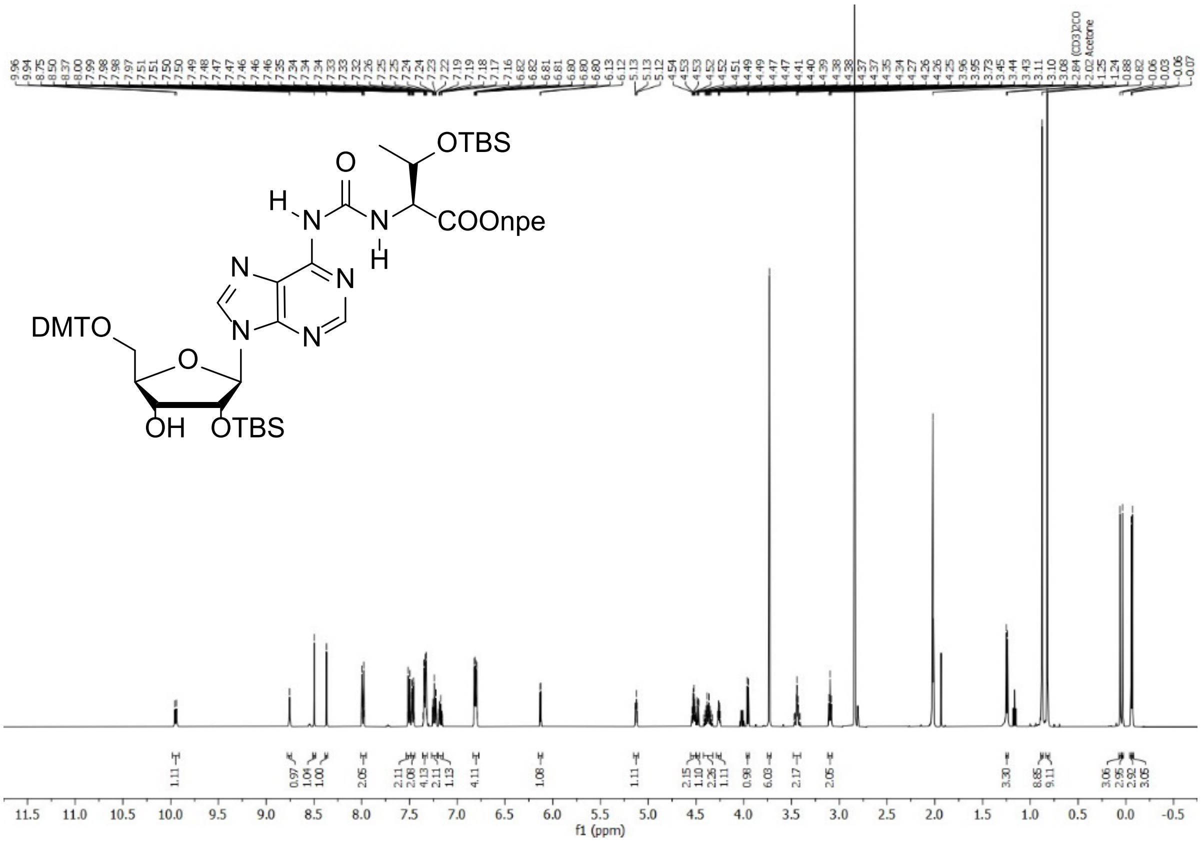

Supplementary Figure 37: ^{1}H-NMR spectrum of compound 5.

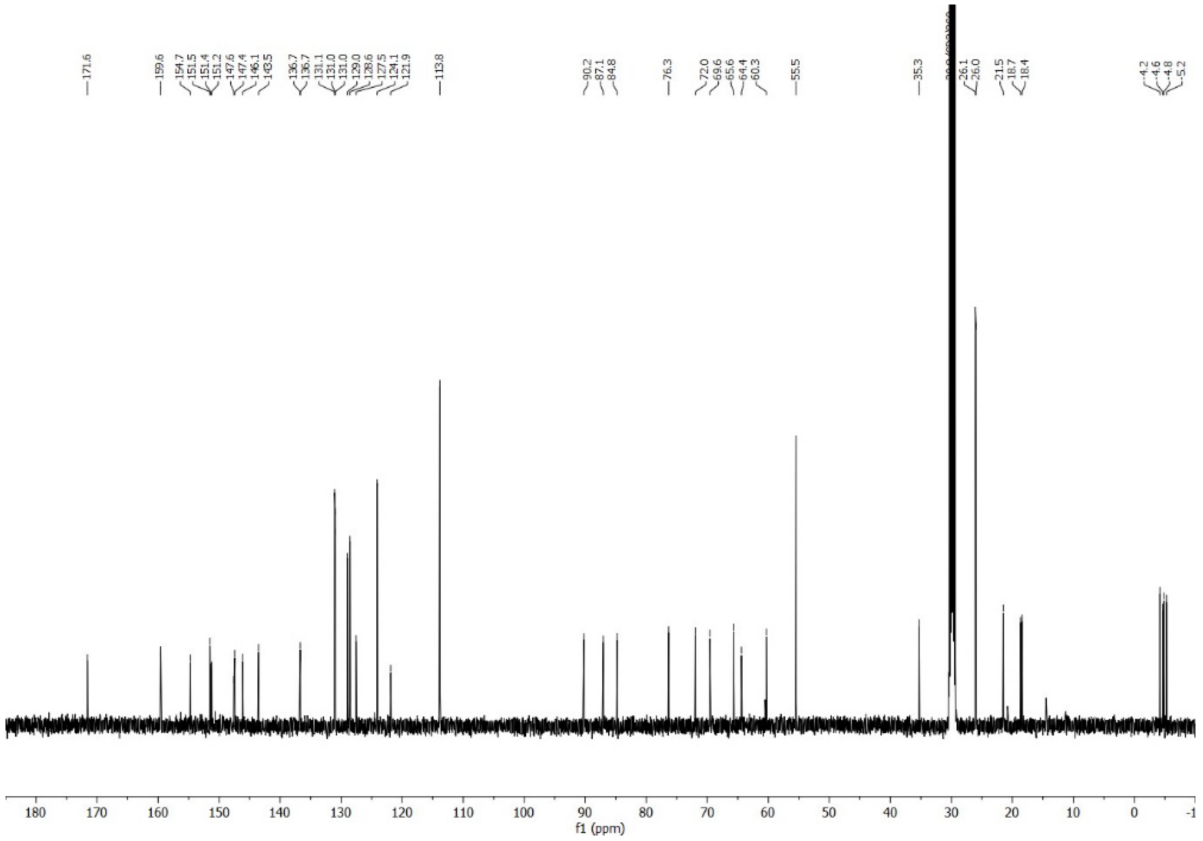

Supplementary Figure 38: ^{13}C-NMR spectrum of compound 5.

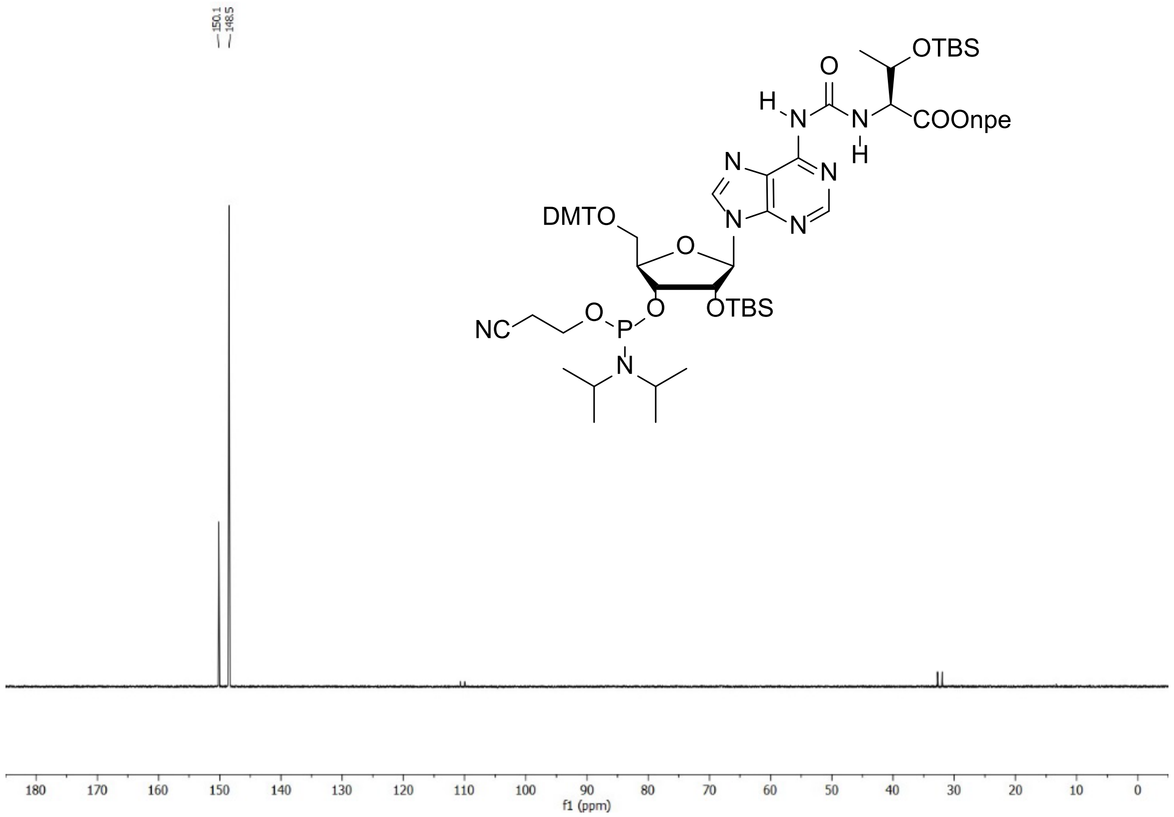

Supplementary Figure 39: ^{31}P-NMR spectrum of compound 6.

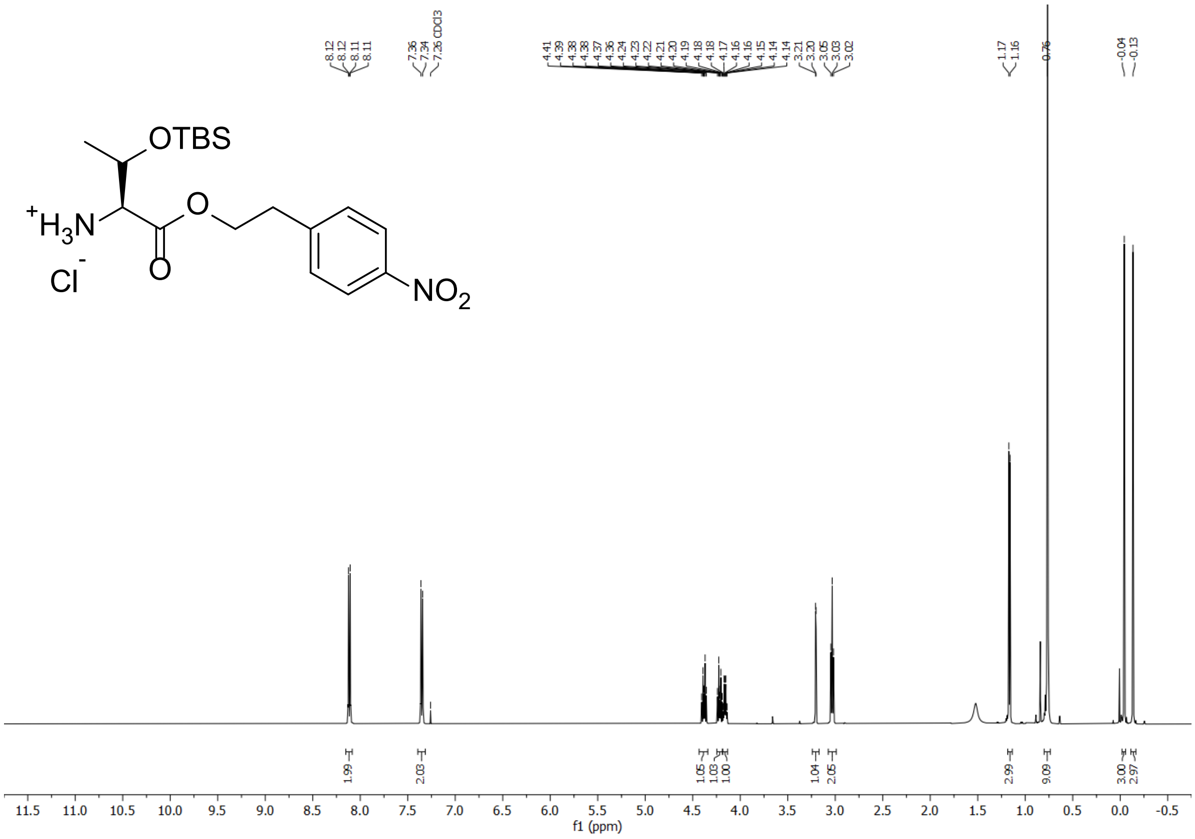

Supplementary Figure 40: ^{1}H-NMR spectrum of compound 9.

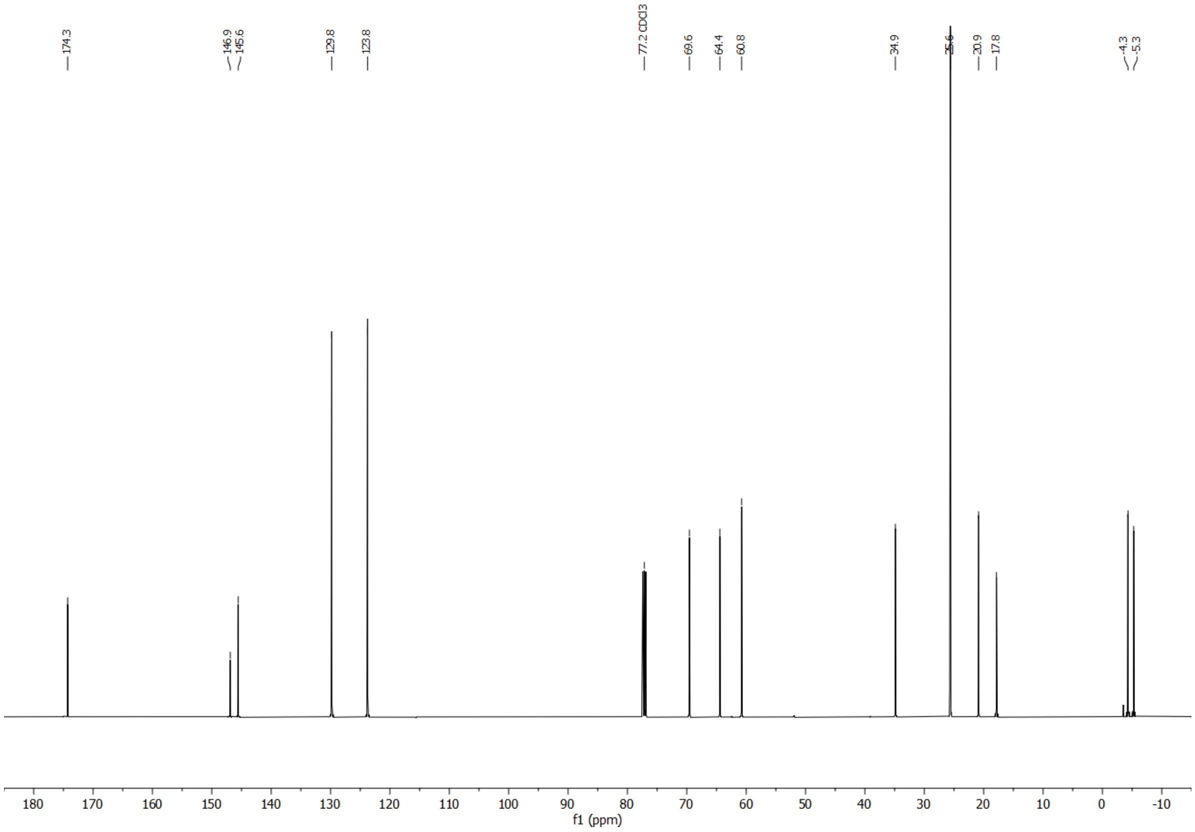

Supplementary Figure 41: ^{13}C-NMR spectrum of compound 9.

SUPPLEMENTARY TABLES

Supplementary Table 1: X-ray data collection and refinement statistics

	**SpTcd[366-485*]**	**SpTcd[366-485#]**
Crystal parameters		
Space group	$P2_12_12_1$	$P2_12_12_1$
Cell constants	a= 32.4 Å	a= 32.5 Å
	b= 55.5 Å	b= 55.2 Å
	c= 69.4 Å	c= 68.2 Å
	α=β=γ= 90°	α=β=γ= 90°
Molecule/AU[a]	1	1
Data collection		
Beam line	X06SA, SLS	X06SA, SLS
Wavelength (Å)	1.0	1.0
Resolution range (Å)[b]	43-1.75 (1.85-1.75)	43-1.55 (1.65-1.55)
No. observations[b]	64,007 (10,018)	113,789 (20,045)
No. unique reflections[b, c]	12,946 (1,906)	17,625 (2,945)
Completeness (%)[b]	98.1 (96.5)	95.7 (95.8)
R_{meas} (%)	8.1 (66.3)	10.9 (210.1)
R_{merge} (%)[b, d]	7.2 (59.6)	11.9 (227.3)
R_{pim} (%)	3.6 (28.9)	4.7 (87.1)
cc1/2	1.0 (0.9)	1.0 (0.5)
I/σ (I)[b]	15.0 (4.0)	7.8 (1.1)
Refinement	REFMAC5	Phenix.refine
Resolution range (Å)	30-1.75	43-1.55
No. refl. working set	12,297	17,609
No. refl. test set	648	879
No. non hydrogen atoms	1,065	1,030
Solvent	56	50
R_{work}/R_{free} (%)[e]	17.2 / 21.2	20.0/22.3
r.m.s.d. bond (Å) / angle (°)[f]	0.003 / 1.114	0.011/1.019
Average B-factor (Å^2)	27.6	25.7
Ramachandran Plot (%)[g]	95.8 / 4.2 / 0.0	96.6/3.4/0.0
PDB code	9HMO	9TZG

The two different data sets were collected from two crystals from the same crystallization condition, but evaluated differently:

*Criteria for resolution cutoff: R_{merge} in highest resolution shell < 60%, I/σ (I) in highest resolution shell > 2

#Criteria for resolution cutoff: cc1/2 in highest resolution shell > 30%, I/σ (I) in highest resolution shell > 1

[a] Asymmetric unit

[b] The values in parentheses for resolution range, No. observations, No. unique reflections, completeness, R_{merge} and I/σ (I) correspond to the highest resolution shell

[c] Data reduction was carried out with XDS and from a single crystal. Friedel pairs were treated as identical reflections

[d] $R_{merge}(I) = \Sigma_{hkl}\Sigma_j \mid I(hkl)_j - \langle I(hkl)\rangle \mid / \Sigma_{hkl} \Sigma_j I(hkl)_j$, where $I(hkl)_j$ is the jth measurement of the intensity of reflection hkl and $\langle I(hkl)\rangle$ is the average intensity

[e] $R = \Sigma_{hkl} \mid |F_{obs}| - |F_{calc}| \mid / \Sigma_{hkl} |Fobs|$, where R_{free} is calculated without a sigma cut off for a randomly chosen 5% of reflections, which were not used for structure refinement, and R_{work} is calculated for the remaining reflections

[f] Deviations from ideal bond lengths/angles

[g] Percentage of residues in favored region / allowed region / outlier region

	ScTcd1$^{50-429}$: AMP*	ScTcd1$^{50-429}$: AMP$^{\#}$
Crystal parameters		
Space group	$P6_522$	$P6_522$
Cell constants	a=b= 259.4 Å<br>c= 236.1 Å<br>α=β=90°<br>γ=120°	a=b= 259.4 Å<br>c= 236.1 Å<br>α=β=90°<br>γ=120°
Molecule/AU[a]	2 homodimers	2 homodimers
Data collection		
Beam line	X06SA, SLS	X06SA, SLS
Wavelength (Å)	1.0	1.0
Resolution range (Å)[b]	49-4.0 (4.1-4.0)	49-3.7 (3.8-3.7)
No. observations[b]	224,488 (16,364)	284,370 (22,698)
No. unique reflections[b, c]	39,562 (2,794)	49,801 (3,806)
Completeness (%)[b]	98.8 (99.7)	98.8 (99.8)
R_{meas} (%)	22.0 (89.0)	28.6 (179.5)
R_{merge} (%)[b, d]	17.1 (81.2)	26.0 (164.1)
R_{pim} (%)	9.2 (36.8)	12.0 (73.5)
cc1/2	1.0 (0.7)	1.0 (0.4)
I/σ (I)[b]	8.1 (2.3)	6.7 (1.2)
Refinement	REFMAC5	Phenix.refine
Resolution range (Å)	30-4.0	49-3.7
No. refl. working set	37,500	49,661
No. refl. test set	1,974	2,484
No. non hydrogen atoms	11,707	11,689
Solvent	17	4
R_{work}/R_{free} (%)[e]	25.5 / 29.9	27.3 / 28.5
r.m.s.d. bond (Å) / angle (°)[f]	0.003 / 1.234	0.002 / 0.551
Average B-factor ($Å^2$)	114.1	116.1
Ramachandran Plot (%)[g]	87.0 / 12.0 / 1.0	93.1 / 6.7 / 0.2
PDB code	9HMP	9TZH

The same diffraction data have been evaluated in two different ways:

*Criteria for resolution cutoff: R_{merge} in highest resolution shell < 60%, I/σ (I) in highest resolution shell > 2

#Criteria for resolution cutoff: cc1/2 in highest resolution shell > 30%, I/σ (I) in highest resolution shell > 1

[a] Asymmetric unit

[b] The values in parentheses for resolution range, No. observations, No. unique reflections, completeness, R_{merge} and I/σ (I) correspond to the highest resolution shell

[c] Data reduction was carried out with XDS and from a single crystal. Friedel pairs were treated as identical reflections

[d] $R_{merge}(I) = \Sigma_{hkl}\Sigma_j \mid I(hkl)_j - \langle I(hkl)\rangle \mid / \Sigma_{hkl} \Sigma_j I(hkl)_j$, where $I(hkl)_j$ is the jth measurement of the intensity of reflection hkl and <I(hkl)> is the average intensity

[e] $R = \Sigma_{hkl} \mid |F_{obs}| - |F_{calc}| \mid / \Sigma_{hkl} |Fobs|$, where R_{free} is calculated without a sigma cut off for a randomly chosen 5% of reflections, which were not used for structure refinement, and R_{work} is calculated for the remaining reflections

[f] Deviations from ideal bond lengths/angles

[g] Percentage of residues in favored region / allowed region / outlier region

Supplementary Table 2. Proteins related to SpTcd$^{366\text{-}485}$ according to Dali search

PDB entry code	*Z-score*	*R.m.s.d. [Å]*	*Identity [%]*	*Protein*
4OGC	5.9	2.9	11	Crystal structure of the Type II-C Cas9 enzyme from Actinomyces naeslundii
5VGB	5.9	2.5	13	Crystal structure of NmeCas9 HNH domain bound to anti-CRISPR AcrIIC1
6JDV	5.7	2.9	15	Crystal structure of Nme1Cas9 in complex with sgRNA and target DNA (ATATGATT PAM) in catalytic state
8FMF	5.6	3.2	13	Structure of CBASS Cap5 from Pseudomonas syringae as an activated tetramer with the cyclic dinucleotide 3'2'-c-diAMP ligand (1 tetramer in the AU)
6J9N	5.6	2.7	14	NmeHNH+AcrIIC3
6GHC	5.5	3.3	16	Modification dependent EcoKMcrA restriction endonuclease
8D2K	5.3	3.4	10	Structure of Acidothermus cellulolyticus Cas9 ternary complex (Cleavage Intermediate 2)
5ZMM	5.2	3.9	12	Structure of the Type IV phosphorothioation-dependent restriction endonuclease ScoMcrA
7MPZ	5.2	2.8	18	HNH Nuclease Domain from G. stearothermophilus Cas9
6GHS	4.9	3.2	10	Modification dependent TagI restriction endonuclease

The Dali server (12) identified only few structures that display 3D similarity to SpTcd$^{366\text{-}485}$ (search was performed on the 24th of April 2024). The 10 best hits are shown and listed according to their Z-score. Redundant protein hits are not listed. The 76th hit of the Dali search was the restriction enzyme PacI (PDB entry code 3LDY) with Z-score of 4.4, r.m.s.d. 3.1 Å and 14% sequence identity. The Z-score cutoff for a strong match depends on the size of the query protein and is for SpTcd$^{366\text{-}485}$ ≥8 (cutoff = n/10-4, where n is the number of residues in the query structure) (13).

Supplementary Table 3: Chemically synthesized tRNA stem loops

Stem loop	Sequence
Ile_AAU (unmodified)	5'-CCGUGCU**AAU**<u>A</u>ACGCGG-3'
Arg_CCU (unmodified)	5'-biotin-UCUCCCU**CCU**<u>A</u>AGGAGA-3'
Arg_CCU_t^{6}A (modified)	5'-biotin-UCUCCCU**CCU**<u>A*</u>AGGAGA-3'

Adenosine 37 is underlined, the anticodon is printed bold and A* represents t^6 modified adenosine.

Supplementary Table 4: Summary of Tcd2 mutants

**Name**	**Mutations retained in the construct**	**Purification successful?***	**tRNA binding?**
Tcd2-14E	R237E, R241E, K242E, K245E, K264E, K267E, R291E, R293E, K371E, K373E, K428E, K431E, R436E, R438E	-	**n.a.**
Tcd2-6E	K371E, K373E, K428E, K431E, R436E, R438E	-	**n.a.**
Tcd2-8E (see Supplementary Figure 17)	R237E, R241E, K242E, K245E, K264E, K267E, R291E, R293E	+	-
Tcd2-8E-E241R	R237E, K242E, K245E, K264E, K267E, R291E, R293E	+	-
Tcd2-8E-E245K	R237E, R241E, K242E, K264E, K267E, R291E, R293E	+	-
Tcd2-8E-E293R	R237E, R241E, K242E, K245E, K264E, K267E, R291E	+	-
Tcd2-8E-E237R-E241R	K242E, K245E, K264E, K267E, R291E, R293E	+	-
Tcd2-8E-E242K-E245K	R237E, R241E, K264E, K267E, R291E, R293E	-	**n.a.**
Tcd2-8E-E264K-E267K	R237E, R241E, K242E, K245E, R291E, R293E	+	-
Tcd2-8E-E291R-E293R	R237E, R241E, K242E, K245E, K264E, K267E	-	**n.a.**
Tcd2-8E-E242K-E245K-E291R-E293R	R237E, R241E, K264E, K267E	+	+

Tcd2-8E-E264K-E267K-E291R-E293R	R237E, R241E, K242E, K245E	+	-
Tcd2-8E-E237R-E241R-E264K-E267K	K242E, K245E, R291E, R293E	+	-
Tcd2-8E-E242K-E245K-E264K-E267K-E291R-E293R	R237E, R241E	+	-
Tcd2-R291A	R291A	+	+
Tcd2-R293E<br>*(see* Supplementary *Figure 24)*	R293E	+	+

* 'successful' means that both Tcd1 and Tcd2 were confirmed to be present after the first Ni affinity chromatography by SDS-PAGE and/or later during the purification by ESI-MS.
n.a. For protein complexes that could not be isolated, tRNA binding could not be determined.

Supplementary Table 5: DNA primer list for protein production in *E. coli*

#	Primer name	Primer sequence (5' → 3')	Use
**1**	Tcd_F	CCAAGATCTGAAAATCTGATACGTGAACAG	Amplification of *S. pombe* $TCD^{304\text{-}1458}$ for insertion into $pET28b^{Kan}$-His_6-SUMO via *Bgl*II
**2**	Tcd_R	CCACTGCAGTCAATAAAGCATTTCCCACATTC	Amplification of *S. pombe* $TCD^{304\text{-}1458}$ for insertion into $pET28b^{Kan}$-His_6-SUMO via *Pst*I/*Pst*I-HF<br>Amplification of *S. pombe* $TCD^{1096\text{-}1458}$ for insertion into pRSET A^{Amp}-His_6-TEV via *Pst*I/*Pst*I-HF
**3**	Tcd_Cterm_F	CCAAGATCTATGACGCGTCCTCGCTTATAC	Amplification of *S. pombe* $TCD^{1096\text{-}1458}$ for insertion into pRSET A^{Amp}-His_6-TEV via *Bgl*II/*Bam*HI-HF
**4**	spTcdThiF_F	TGCTAAATACtaaATGGATCCCATTTCTACCATGAC	Q5 mutagenesis primer to introduce a stop codon after the ThiF domain in $pET28b^{Kan}$-His_6-SUMO-$TCD^{304\text{-}1458}$
**5**	spTcdThiF_R	ATAGAAGTCAAAACATAAGTAGCAATTGTAAG	
**6**	pRSET-Tcd1_F	CCAGGATCCGACCACTTATTTAGGGAAC	Amplification of *S. cerevisiae* $TCD1^{148\text{-}1290}$ for insertion into pRSET A^{Amp}-His_6-TEV via *BamH*I-HF
**7**	pRSET-Tcd1_R	CCACTGCAGCTAAGAATAGTACTCCTCC	Amplification of *S. cerevisiae* $TCD1^{148\text{-}1290}$ for insertion into pRSET A^{Amp}-His_6-TEV or pBEVY-$L^{Amp,\ Leu}$ via PstI-HF
**8**	Tcd1-E84A_F	CGGAGCTGGAGCGGTGGGATCGT	Q5 mutagenesis primer for pRSET A^{Amp}-His_6-TEV-$TCD1^{148\text{-}1290}$ to generate pRSET A^{Amp}-His_6-TEV-$TCD1^{148\text{-}1290}$-E84G
**9**	Tcd1-E84A_R	ACTATAACTATATACTGTTCTTTGATCTTC	
**10**	scTcd1ThiF_F	GTCAGGCTATtaaATGAAAGAAAATGAGGTCAAAAATAG	Q5 mutagenesis primer to introduce a stop codon after the ThiF domain in pRSET A^{Amp}-His_6-TEV-$TCD1^{148\text{-}1290}$
**11**	scTcd1ThiF_R	ACTTTGGTTAAAATCCATGTCGC	
**12**	T7_F	TAATACGACTCACTATAGGG	Amplification of $TCD1^{148\text{-}1290}$ and flanking regions from pRSET A^{Amp}-His_6-TEV-$TCD1^{148\text{-}1290}$ for subsequent insertion into MSC2 of pACYCDuet-1 via *Nde*I-HF and *Kpn*I-HF
**13**	T7_R	GCTAGTTATTGCTCAGCGG	
**14**	AatII_pACYC_F	TTTGCGCCATTCGATGACGTCCGGGATCTCGACG	Quikchange mutagenesis primer for conversion of the *Eco*NI

**15**	AatII_pACYC_R	CGTCGAGATCCCGGACGTCATCGAATGGCGCAAA	restriction site into a *Aat*II recognition motif in pACYC^{Cm}-His$_6$-TEV-*TCD1*$^{148-1290}$
**16**	tRNA^{Thr}_F	CCAACGACGTCCGGGATCTCGACGCTCTCCC	Amplification of tRNA$^{Thr(UGU)}$ for insertion into pACYC^{Cm}-His$_6$-TEV-*TCD1*$^{148-1290*}$ (MCS1) via *Aat*II-HF and *Hind*III-HF
**17**	tRNA^{Thr}_R	CTGTTCGACTTAAGCATTATGCG	
**18**	tRNA^{Arg}/^{Asn}_F	CGACTCCTGCATTAGGCCCATCAAAAAA	Amplification of tRNA$^{Arg(CCT)}$ or tRNA$^{Asn(GTT)}$ for insertion into pACYC^{Cm}-His$_6$-TEV-*TCD1*$^{148-1290*}$ (MCS1) via Gibson assembly
**19**	tRNA^{Arg}/^{Asn}_R	TATGCGGCCGCAAGCTTAAAAAAAATCCT	
**20**	pACYC-tRNA_F	AAGCTTGCGGCCGC	Amplification of pACYCDuet-1^{Cm} for Gibson assembly with tRNA fragments
**21**	pACYC-tRNA_R	CCTAATGCAGGAGTCGCATAAGG	
**22**	pRSET-Tcd2_F	CCAAGATCTGAACAGTTTATTCGCCAATC	Amplification of *S. cerevisiae* *TCD2*$^{184-1344}$ for insertion into pRSET A^{Amp}-His$_6$-TEV via *Bgl*II/*Bam*HI-HF and *Pst*I/*Pst*I-HF
**23**	pRSET-Tcd2_R	CCACTGCAGTCATCTGAATTGAGAGTAATAAGC	
**24**	8WT_F	CCGAAGAAGACCCGCTGGCTCGTGTTGTTCGT	Amplification of the 8WT fragment from pETDuet^{Amp}-His$_6$-SUMO-*TCD2*$^{184-1344}$ for insertion into pETDuet^{Amp}-His$_6$-SUMO-*TCD2*$^{184-1344}$-*14E* to create pETDuet^{Amp}-His$_6$-SUMO-*TCD2*$^{184-1344}$-*6E*
**25**	8WT_R	GTACCCAGAACCGGCAGGATACGAACACGGAA	
**26**	8WT_vec_F	TGAAAGACTTCCGTGTTCGTATCCTGCCGGTTCTGGGTACTATGCC	Amplification of pETDuet^{Amp}-His$_6$-SUMO-*TCD2*$^{184-1344}$-*14E* for insertion of the 8WT fragment by Gibson assembly *14E* to create pETDuet^{Amp}-His$_6$-SUMO-*TCD2*$^{184-1344}$-*6E*
**27**	8WT_vec_R	AGTTTACGACGAACAACACGAGCCAGCGGGTCTTCTTCG	
**28**	6WT_F	ACCTGGTTGAAGAAGTTTTCAAAGGTAAATCT	Amplification of the 6WT fragment from pETDuet^{Amp}-His$_6$-SUMO-*TCD2*$^{184-1344}$ for insertion into pETDuet^{Amp}-His$_6$-SUMO-*TCD2*$^{184-1344}$-*14E* to create pETDuet^{Amp}-His$_6$-SUMO-*TCD2*$^{184-1344}$-*8E*
**29**	6WT_R	TGAGAGTAGTAAGCTTCTTCACGGAAACGCTG	
**30**	6WT_vec_F	TGGTTTCTCAGCGTTTCCGTGAAGAAGCTTACTACTCTCAGTTCCGTTAACTGC	Amplification of pETDuet^{Amp}-His$_6$-SUMO-*TCD2*$^{184-1344}$-*14E* for insertion of the 6WT fragment by Gibson assembly to create pETDuet^{Amp}-His$_6$-SUMO-*TCD2*$^{184-1344}$-*8E*
**31**	6WT_vec_R	GAGATCGGAGATTTACCTTTGAAAACTTCTTCAACCAG	

**32**	Tcd2-8E-E241R_F	AGTTGTTCGTCGTGAACTGAAAGAACGTGGTATC	Q5 mutagenesis primer for pETDuet^{Amp}-His$_6$-SUMO-*TCD2*$^{184-1344}$-*8E* to generate pETDuet^{Amp}-His$_6$-SUMO-*TCD2*$^{184-1344}$-*8E*-E241R
**33**	Tcd2-8E-E241R_R	TCAGCCAGCGGGTCTTCT	
**34**	Tcd2-8E-E242K_F	TGTTCGTGAAAAACTGAAAGAACGTG	Q5 mutagenesis primer for pETDuet^{Amp}-His$_6$-SUMO-*TCD2*$^{184-1344}$-*8E* to generate pETDuet^{Amp}-His$_6$-SUMO-*TCD2*$^{184-1344}$-*8E*-E242K
**35**	Tcd2-8E-E242K_R	ACTTCAGCCAGCGGGTCT	
**36**	Tcd2-8E-E245K_F	AGAACTGAAAAAACGTGGTATCC	Q5 mutagenesis primer for pETDuet^{Amp}-His$_6$-SUMO-*TCD2*$^{184-1344}$-*8E* to generate pETDuet^{Amp}-His$_6$-SUMO-*TCD2*$^{184-1344}$-*8E*-E245K
**37**	Tcd2-8E-E245K_R	TCACGAACAACTTCAGCC	
**38**	Tcd2-8E-E264K_F	ACCGGACCCGAAAAAAGCTGAAC	Q5 mutagenesis primer for pETDuet^{Amp}-His$_6$-SUMO-*TCD2*$^{184-1344}$-*8E* to generate pETDuet^{Amp}-His$_6$-SUMO-*TCD2*$^{184-1344}$-*8E*-E264K
**39**	Tcd2-8E-E264K_R	TTTTCAGCAGAGAAAACAACCG	
**40**	Tcd2-8E-E267K_F	GGAAAAAGCTAAACTGCTGCCGC	Q5 mutagenesis primer for pETDuet^{Amp}-His$_6$-SUMO-*TCD2*$^{184-1344}$-*8E* to generate pETDuet^{Amp}-His$_6$-SUMO-*TCD2*$^{184-1344}$-*8E*-E267K
**41**	Tcd2-8E-E267K_R	GGGTCCGGTTTTTCAGCAG	
**42**	Tcd2-8E-E293R_F	CTTCGAAGTTCGTATCCTGCCGG	Q5 mutagenesis primer for pETDuet^{Amp}-His$_6$-SUMO-*TCD2*$^{184-1344}$-*8E* to generate pETDuet^{Amp}-His$_6$-SUMO-*TCD2*$^{184-1344}$-*8E*-E293R
**43**	Tcd2-8E-E293R_R	TCTTTCAGAGCAGACAGTTC	
**44**	E237RinE241R_F	CCCGCTGGCTCGTGTTGTTCGTC	Q5 mutagenesis primer for pETDuet^{Amp}-His$_6$-SUMO-*TCD2*$^{184-1344}$-*8E*-E241R to generate pETDuet^{Amp}-His$_6$-SUMO-*TCD2*$^{184-1344}$-*8E*-E237R-E241R
**45**	E237RinE241R_R	TCTTCTTCGGTGGTAGCC	
**46**	E242KinE245K_F	TGTTCGTGAAAAACTGAAAAAACGTGG	Q5 mutagenesis primer for pETDuet^{Amp}-His$_6$-SUMO-*TCD2*$^{184-1344}$-*8E*-E245K to generate pETDuet^{Amp}-His$_6$-SUMO-*TCD2*$^{184-1344}$-*8E*-E242K-E245K
**47**	E242KinE245K_R	ACTTCAGCCAGCGGGTCT	
**48**	E264KinE267K_F	ACCGGACCCGAAAAAAGCTAAAC	Q5 mutagenesis primer for pETDuet^{Amp}-His$_6$-SUMO-*TCD2*$^{184-1344}$-*8E*-E267K to generate pETDuet^{Amp}-His$_6$-SUMO-*TCD2*$^{184-1344}$-*8E*-E264K-E267K
**49**	E264KinE267K_R	TTTTCAGCAGAGAAAACAACC	

**50**	E291RinE293R_F	GAAAGACTTCCGTGTTCGTATCCTGCC	Q5 mutagenesis primer for pETDuet^{Amp}-His$_6$-SUMO-*TCD2*$^{184\text{-}1344}$-*8E*-E293R to generate pETDuet^{Amp}-His$_6$-SUMO-*TCD2*$^{184\text{-}1344}$-*8E*-E291R-E293R
**51**	E291RinE293R_F	AGAGCAGACAGTTCGTCA	
**52**	E242K_E245K_F	AAAAAACGTGGTATCCTGTCTGGT	Q5 mutagenesis primer to introduce mutation E242K and E245K in *TCD2*$^{184\text{-}1344}$-8E based constructs; Glu237 and Glu241 retained
**53**	E242K_E245K_R	CAGTTTTTCACGAACAACTTCAGC	
**54**	E291R_E293R_F	CGAACTGTCTGCTCTGAAAGACTTCCGTGTTCGTATCCTGCCGGTTCTGGGTACTATGC	Quikchange mutagenesis primer to introduce mutation E291R and E293R in *TCD2*$^{184\text{-}1344}$-8E based constructs
**55**	E291R_E293R_R	GCATAGTACCCAGAACCGGCAGGATACGAACACGGAAGTCTTTCAGAGCAGACAGTTCG	
**56**	237-241_F	GAAGAAGACCCGCTGGCTCGTGTTGTTCGTCGTGAACTG	Quikchange mutagenesis primer to introduce mutation E237R and E241R in *TCD2*$^{184\text{-}1344}$-8E based constructs; Glu242 retained
**57**	237-241_R	CAGTTCACGACGAACAACACGAGCCAGCGGGTCTTCTTC	
**58**	264-267_F	GAAAAACCGGACCCGAAAAAAGCTAAACTGCTGCCGCTGC	Quikchange mutagenesis primer to introduce mutations E264K and E267K in *TCD2*$^{184\text{-}1344}$-8E based constructs
**59**	264-267_R	GCAGCGGCAGCAGTTTAGCTTTTTTCGGGTCCGGTTTTTC	
**60**	R291A_F	GAAAGACTTCGCGGTTCGTATCCTGCCG	Q5 mutagenesis primer for pETDuet^{Amp}-His$_6$-SUMO-*TCD2*$^{184\text{-}1344}$ to generate pETDuet^{Amp}-His$_6$-SUMO-*TCD2*$^{184\text{-}1344}$-R291A
**61**	R291A_R	AGAGCAGACAGTTCGTCA	
**62**	R293E_F	TACTATGCCGTCTCTGTTCGGTC	Q5 mutagenesis primer for pETDuet^{Amp}-His$_6$-SUMO-*TCD2*$^{184\text{-}1344}$ to generate pETDuet^{Amp}-His$_6$-SUMO-*TCD2*$^{184\text{-}1344}$-R293E
**63**	R293E_R	CCCAGAACCGGCAGGATTTCAACACGGAAG	
**64**	scTcd2ThiF_F	CTCTGACAAAtaaCTGGAACCGGTTGAAGG	Q5 mutagenesis primer to introduce a stop codon after the ThiF domain in pETDuet^{Amp}-His$_6$-SUMO-*TCD2*$^{184\text{-}1344}$
**65**	scTcd2ThiF_R	ATGTTAGATAAGATCCAGGTGGTG	

**66**	Tcd1_F	TTAAGTATAAGAAGGAGATATACATATGGACCACTTATTTAGGGAACAATTGG	Amplification of *S. cerevisiae* *TCD1*$^{148\text{-}1290}$ for insertion into MCS2 of pETDuet^{Amp}-His$_6$-SUMO-*TCD2*$^{184\text{-}1344}$ via Gibson assembly
**67**	Tcd1_R	GCAGCAGCCTAGGTTAACTAAGAATAGTACTCCTCCTCTTTAAAAAGCCT	
**68**	Tcd1_vec_F	TTAACCTAGGCTGCTGCCAC	Amplification of pETDuet^{Amp}-His$_6$-SUMO-*TCD2*$^{184\text{-}1344}$ for insertion of *S. cerevisiae* *TCD1*$^{148\text{-}1290}$ via Gibson assembly
**69**	Tcd1_vec_R	ATGTATATCTCCTTCTTATACTTAACTAATATACTAAGATGGGGAA	
**70**	TcdA_F	ACAGATCGGTGGATCCATGTCTGTGGTA	Insertion of TcdA (*E. coli*) into pETDuet^{Amp}
**71**	TcdA_R	GCCGTGTACAATACGATTAACCCTGACG	Insertion of TcdA (*E. coli*) into pETDuet^{Amp}
**72**	TcdA_vec_F	TCGTATTGTACACGGCCGC	Insertion of TcdA (*E. coli*) into pETDuet^{Amp}
**73**	TcdA_vec_R	GGATCCACCGATCTGTTCACG	Insertion of TcdA (*E. coli*) into pETDuet^{Amp}

Underlined bases represent recognition sites for restriction endonucleases used for cloning.

* The asterisk denotes a variant of pACYC^{Cm}-His$_6$-TEV-*TCD1*$^{148\text{-}1290}$ in which the *Eco*NI restriction site has been mutated to that of *Aat*II. For details see material and methods.

Supplementary Table 6: *E. coli* plasmids used in this study

Vector	Description
pET28b^{Kan}-His$_6$-SUMO-*TCD*$^{304\text{-}1458}$	Production of *S. pombe* His$_6$-SUMO-Tcd$^{102\text{-}485}$
pRSET A^{Amp}-His$_6$-TEV-*TCD*$^{1096\text{-}1458}$	Production of *S. pombe* His$_6$-TEV-Tcd$^{366\text{-}485}$
pET28b^{Kan}-His$_6$-SUMO-*TCD*$^{304\text{-}1071}$	Production of *S. pombe* His$_6$-SUMO-Tcd$^{102\text{-}358}$ (ThiF domain)
pRSET A^{Amp}-His$_6$-TEV-*TCD1*$^{148\text{-}1290}$	Production of *S. cerevisiae* His$_6$-TEV-Tcd1$^{50\text{-}429}$
pRSET A^{Amp}-His$_6$-TEV-*TCD1*$^{148\text{-}1290}$*-E84G*	Production of *S. cerevisiae* His$_6$-TEV-Tcd1-E84G
pRSET A^{Amp}-His$_6$-TEV-*TCD1*$^{148\text{-}912}$	Production of *S. cerevisiae* His$_6$-TEV-Tcd1$^{50\text{-}304}$ (ThiF domain)
pACYCDuet-1^{Cm}-His$_6$-TEV-*TCD1*$^{148\text{-}1290}$	Together with pETDuet-1^{Amp}- His$_6$-SUMO-*TCD2*$^{184\text{-}1344}$ used for co-production of *S. cerevisiae* His$_6$-TEV-Tcd1$^{50\text{-}429}$ and His$_6$-SUMO-Tcd2$^{62\text{-}447}$
pACYCDuet-1^{Cm}-*tRNA*$^{Thr(UGU)}$–His$_6$-TEV-*TCD1*$^{148\text{-}1290}$*<br>pACYCDuet-1^{Cm}-*tRNA*$^{Arg(CCU)}$–His$_6$-TEV-*TCD1*$^{148\text{-}1290}$*	Together with pETDuet-1^{Amp}- His$_6$-SUMO-*TCD2*$^{184\text{-}1344}$ used for co-production of *S. cerevisiae* His$_6$-TEV-Tcd1$^{50\text{-}429}$, His$_6$-SUMO-Tcd2$^{62\text{-}447}$ and tRNA$^{Thr(UGU)}$ or tRNA$^{Arg(CCU)}$
pRSET A^{Amp}-His$_6$-TEV-*TCD2*$^{184\text{-}1344}$	Production of *S. cerevisiae* His$_6$-TEV-Tcd2$^{62\text{-}447}$
pETDuet-1^{Amp}-His$_6$-SUMO-*TCD2*$^{184\text{-}1344}$	Together with pACYCDuet-1^{Cm}-*tRNA*^{XXX}–*His*$_6$*-TEV-TCD1*$^{148\text{-}1290}$ used for co-production of *S. cerevisiae* His$_6$-TEV-Tcd1$^{50\text{-}429}$, His$_6$-SUMO-Tcd2$^{62\text{-}447}$ and tRNA$^{Thr(UGU)}$ or tRNA$^{Arg(CCU)}$
pETDuet-1^{Amp}-His$_6$-SUMO-*TCD2*$^{184\text{-}1344}$*-14E*	Production of mutant Tcd2$^{62\text{-}447}$ (Supplementary Table 4)
pETDuet-1^{Amp}-His$_6$-SUMO-*TCD2*$^{184\text{-}1344}$*-6E*	
pETDuet-1^{Amp}-His$_6$-SUMO-*TCD2*$^{184\text{-}1344}$*-8E*	
pETDuet-1^{Amp}-His$_6$-SUMO-*TCD2*$^{184\text{-}1344}$*-E241R*	
pETDuet-1^{Amp}-His$_6$-SUMO-*TCD2*$^{184\text{-}1344}$*-E245K*	
pETDuet-1^{Amp}-His$_6$-SUMO-*TCD2*$^{184\text{-}1344}$*-E293R*	
pETDuet-1^{Amp}-His$_6$-SUMO-*TCD2*$^{184\text{-}1344}$*-E237R-E241R*	

pETDuet-1^{Amp}-His$_6$-SUMO-*TCD2*$^{184-1344}$-*E242K-E245K*	
pETDuet-1^{Amp}-His$_6$-SUMO-*TCD2*$^{184-1344}$-*E264K-E267K*	Production of mutant Tcd2$^{62-447}$ (Supplementary Table 4)
pETDuet-1^{Amp}-His$_6$-SUMO-*TCD2*$^{184-1344}$-*E291R-E293R*	
pETDuet-1^{Amp}-His$_6$-SUMO-*TCD2*$^{184-1344}$-*E242K-E245K-E291R-E293R*	
pETDuet-1^{Amp}-His$_6$-SUMO-*TCD2*$^{184-1344}$-*E264K-E267K-E291R-E293R*	
pETDuet-1^{Amp}-His$_6$-SUMO-*TCD2*$^{184-1344}$-*E237R-E241R-E264K-E267K*	
pETDuet-1^{Amp}-His$_6$-SUMO-*TCD2*$^{184-1344}$-*E242K-E245K-E264K-E267K-E291R-E293R*	
pETDuet-1^{Amp}-His$_6$-SUMO-*TCD2*$^{184-1344}$-*R291A*	
pETDuet-1^{Amp}-His$_6$-SUMO-*TCD2*$^{184-1344}$-*R293E*	
pETDuet-1^{Amp}-His$_6$-SUMO-*TCD2*$^{184-960}$	Production of His$_6$-SUMO-Tcd2$^{62-320}$ (ThiF domain)
pETDuet-1^{Amp}-His$_6$-SUMO-*TCD2*$^{184-1344}$–*TCD1*$^{148-1290}$	Co-production of His$_6$-SUMO-Tcd2$^{62-447}$ and untagged Tcd1$^{50-429}$ from a single plasmid
pETDuet-1^{Amp}-His$_6$-SUMO-*tcdA*	Production of *E. coli* His$_6$-SUMO-TcdA

Supplementary Table 7: Synthetic *S. cerevisiae* tRNA genes for co-expression in *E. coli*

Gene name	Gene sequence (5' → 3')
tRNA$^{Thr(UGU)}$ with lpp promoter and rrnc terminator	GGATCTCGACGCTCTCCCTTATGCGACTCCTGCATTAGGCC<br>CATCAAAAAAATATTCTCAACATAAAAAACTTTGTGTAATACTT<br>GTAACGCTGCCTCCTTAGCTTAGTGGTAGAGCGTTGCACT**TG**<br>**T**AATGCAAAGGTCGTTAGTTCAATTCTGACAGGTGGCAATCC<br>TTAGCGAAAGCTAAGGATTTTTTTTAAGCTTGCGGCCGCATA<br>ATGCTTAAGTCGAACAG
tRNA$^{Arg(CCU)}$ with lpp promoter and rrnc terminator	CCAACGACGTCCGGGATCTCGACGCTCTCCCTTATGCGACT<br>CCTGCATTAGGCCCATCAAAAAAATATTCTCAACATAAAAAAC<br>TTTGTGTAATACTTGTAACGCTGTTCCGTTGGCGTAATGGTA<br>ACGCGTCTCCCT**CCT**AAGGAGAAGACTGCGGGTTCGAGTCC<br>CGTACGGAACGATCCTTAGCGAAAGCTAAGGATTTTTTTTAA<br>GCTTGCGGCCGCATAATGCTTAAGTCGAACAG

Sequences encoding the *lpp* promoter and *rrnc* terminator are shaded in gray. The recognition site for the restriction endonuclease *Hin*dIII is underlined and the sequence for the anticodon is printed bold.

Supplementary Table 8: Synthetic genes and gene strands

Gene name	Gene sequence (5' → 3')
TCD2 $^{184\text{-}1344}$ optimized for *E. coli* codon usage	GAACAGTTCATCCGTCAGTCTCTGAAAAACAACGTTGAATT CCTGGGTGAAGACACCATCGAAAAACTGTCTAACCAGTACG TTGTTGTTGTTGGTGCTGGTGGTGTTGGTTCTTGGGTTGTT AACTCTCTGGTTCGTTCTGGTTGCCGTAAAATCCGTGTTGT TGACTTCGACCAGGTTTCTCTGTCTTCTCTGAACCGTCACT CTTGCGCTATCCTGAACGACGTTGGTACTCCGAAAGTTGAA TGCCTGCGTCGTCACATGCGTGAAATCGCTCCGTGGTGCG AAATCGACCCGATCAACGAACTGTGGACCCTGCAAAACGG TGAACGTCTGACCCTGGGTAACGGTACTCCGGACTTCATC GTTGACTGCATCGACAACATCGACACCAAAGTTGACCTGCT GGAATTCGCTTACAACCACGGTATCAAAGTTATCTCTTCTAT GGGTGCTTCTGCTAAATCTGACCCGACCAAACTGAACGTTG GTGACCTGGCTACCACCGAAGAAGACCCGCTGGCTCGTGT TGTTCGTCGTAAACTGAAAAAACGTGGTATCCTGTCTGGTA TCCCGGTTGTTTTCTCTGCTGAAAAACCGGACCCGAAAAAA GCTAAACTGCTGCCGCTGCCGGACGAAGAATACGAACGTG GTAAAGTTGACGAACTGTCTGCTCTGAAAGACTTCCGTGTT CGTATCCTGCCGGTTCTGGGTACTATGCCGTCTCTGTTCGG TCTGACCATCACCACCTGGATCTTATCTAACATCTCTGACAA ACCGCTGGAACCGGTTGAAGGTAAAAACCGTATCAAAGTTT ACGACGGTATCTACCAGTCTCTGGCTGGTCAGATGTCTCGT GTTGGTATCCCGTCTCAGCGTATCCCGCTGGCTCTGAAAGA CGTTTCTTACCTGGTTGAAGAAGTTTTCAAAGGTAAATCTCC GATCTCTGGTATCTCTACCCGTCTGACCCTGACCAAATGGG ACCCGTCTAAACCGATCTCTCTGCAAAACGTTGTTGTTCTG ACCAAAAACGAACAGAAAGTTCACGAAGACCGTGTTCTGAA AGGTAAAGAATCTCTGCAAGACGTTTACGACGCTAAAGTTC TGAAACTGGTTTCTCAGCGTTTCCGTGAAGAAGCTTACTAC TCTCAGTTCCGTTAA
His $_{6}$ ***-SUMO*** ^{star}	ATGGGTCATCATCACCATCATCACGGTTCTTTGCAGGATTC TGAAGTCAATCAGGAAGCAAAACCAGAAGTTAAACCGGAAG TAAAGCCAGAAACGCACATTAACCTTAAAGTGAGTGATGGA TCAAGCGAGATTTTCTTCAAGATCAAGAAAACAACTCCCTTA AGGAGATTGATGGAAGCGTTTGCCAAAAGACAAGGAAAGG AAATGGACTCCTTAACCTTTCTGTATGATGGCATTGAGATAC AAGCTGATCAAGCTCCTGAAGATCTAGATATGGAGGACAAT GACATCATTGAAGCACATAGAGAGCAAATAGGTGGG
TCD2 $^{184\text{-}1344}$ ***-14E*** optimized for *E. coli* codon usage	GAACAGTTCATCCGTCAGTCTCTGAAAAACAACGTTGAATT CCTGGGTGAAGACACCATCGAAAAACTGTCTAACCAGTACG TTGTTGTTGTTGGTGCTGGTGGTGTTGGTTCTTGGGTTGTT AACTCTCTGGTTCGTTCTGGTTGCCGTAAAATCCGTGTTGT TGACTTCGACCAGGTTTCTCTGTCTTCTCTGAACCGTCACT CTTGCGCTATCCTGAACGACGTTGGTACTCCGAAAGTTGAA TGCCTGCGTCGTCACATGCGTGAAATCGCTCCGTGGTGCG AAATCGACCCGATCAACGAACTGTGGACCCTGCAAAACGG

TGAACGTCTGACCCTGGGTAACGGTACTCCGGACTTCATC
GTTGACTGCATCGACAACATCGACACCAAAGTTGACCTGCT
GGAATTCGCTTACAACCACGGTATCAAAGTTATCTCTTCTAT
GGGTGCTTCTGCTAAATCTGACCCGACCAAACTGAACGTTG
GTGACCTGGCTACCACCGAAGAAGACCCGCTGGCTGAAGT
TGTTCGTGAAGAACTGAAAGAACGTGGTATCCTGTCTGGTA
TCCCGGTTGTTTTCTCTGCTGAAAAACCGGACCCGGAAAAA
GCTGAACTGCTGCCGCTGCCGGACGAAGAATACGAACGTG
GTAAAGTTGACGAACTGTCTGCTCTGAAAGACTTCGAAGTT
GAAATCCTGCCGGTTCTGGGTACTATGCCGTCTCTGTTCGG
TCTGACCATCACCACCTGGATCTTATCTAACATCTCTGACAA
ACCGCTGGAACCGGTTGAAGGTAAAAACCGTATCAAAGTTT
ACGACGGTATCTACCAGTCTCTGGCTGGTCAGATGTCTCGT
GTTGGTATCCCGTCTCAGCGTATCCCGCTGGCTCTGAAAGA
CGTTTCTTACCTGGTTGAAGAAGTTTTCGAAGGTGAATCTC
CGATCTCTGGTATCTCTACCCGTCTGACCCTGACCAAATGG
GACCCGTCTAAACCGATCTCTCTGCAAAACGTTGTTGTTCT
GACCAAAAACGAACAGAAAGTTCACGAAGACCGTGTTCTGA
AAGGTAAAGAATCTCTGCAAGACGTTTACGACGCTGAAGTT
CTGGAACTGGTTTCTCAGGAATTCGAAGAAGAAGCTTACTA
CTCTCAGTTCCGTTAA

Mutations in the TCD2$^{184\text{-}1344}$-14E gene

x DNA mutation

X mutant residue

GAACAGTTCATCCGTCAGTCTCTGAAAAACAACGTTGAATTCCTGGGTGAAGACACCATC
E Q F I R Q S L K N N V E F L G E D T I
GAAAAACTGTCTAACCAGTACGTTGTTGTTGTTGGTGCTGGTGGTGTTGGTTCTTGGGTT
E K L S N Q Y V V V V G A G G V G S W V
GTTAACTCTCTGGTTCGTTCTGGTTGCCGTAAAATCCGTGTTGTTGACTTCGACCAGGTT
V N S L V R S G C R K I R V V D F D Q V
TCTCTGTCTTCTCTGAACCGTCACTCTTGCGCTATCCTGAACGACGTTGGTACTCCGAAA
S L S S L N R H S C A I L N D V G T P K
GTTGAATGCCTGCGTCGTCACATGCGTGAAATCGCTCCGTGGTGCGAAATCGACCCGATC
V E C L R R H M R E I A P W C E I D P I
AACGAACTGTGGACCCTGCAAAACGGTGAACGTCTGACCCTGGGTAACGGTACTCCGGAC
N E L W T L Q N G E R L T L G N G T P D
TTCATCGTTGACTGCATCGACAACATCGACACCAAAGTTGACCTGCTGGAATTCGCTTAC
F I V D C I D N I D T K V D L L E F A Y
AACCACGGTATCAAAGTTATCTCTTCTATGGGTGCTTCTGCTAAATCTGACCCGACCAAA
N H G I K V I S S M G A S A K S D P T K
CTGAACGTTGGTGACCTGGCTACCACCGAAGAAGACCCGCTGGCT**gaa**GTTGTTCGT**gaa**
L N V G D L A T T E E D P L A E V V R E
gAACTGAAA**g**AACGTGGTATCCTGTCTGGTATCCCGGTTGTTTTCTCTGCTGAAAAACCG
E L K E R G I L S G I P V V F S A E K P
GACCCG**g**AAAAAGCT**g**AACTGCTGCCGCTGCCGGACGAAGAATACGAACGTGGTAAAGTT
D P E K A E L L P L P D E E Y E R G K V
GACGAACTGTCTGCTCTGAAAGACTTC**gaa**GTT**gaa**ATCCTGCCGGTTCTGGGTACTATG
D E L S A L K D F E V E I L P V L G T M
CCGTCTCTGTTCGGTCTGACCATCACCACCTGGATCTTATCTAACATCTCTGACAAACCG
P S L F G L T I T T W I L S N I S D K P
CTGGAACCGGTTGAAGGTAAAAACCGTATCAAAGTTTACGACGGTATCTACCAGTCTCTG
L E P V E G K N R I K V Y D G I Y Q S L
GCTGGTCAGATGTCTCGTGTTGGTATCCCGTCTCAGCGTATCCCGCTGGCTCTGAAAGAC
A G Q M S R V G I P S Q R I P L A L K D
GTTTCTTACCTGGTTGAAGAAGTTTTC**g**AAGGT**g**AATCTCCGATCTCTGGTATCTCTACC
V S Y L V E E V F E G E S P I S G I S T
CGTCTGACCCTGACCAAATGGGACCCGTCTAAACCGATCTCTCTGCAAAACGTTGTTGTT
R L T L T K W D P S K P I S L Q N V V V
CTGACCAAAAACGAACAGAAAGTTCACGAAGACCGTGTTCTGAAAGGTAAAGAATCTCTG
L T K N E Q K V H E D R V L K G K E S L
CAAGACGTTTACGACGCT**g**AAGTTCTG**g**AACTGGTTTCTCAG**gaa**TTC**gaa**GAAGAAGCT
Q D V Y D A E V L E L V S Q E F E E E A

```
TACTACTCTCAGTTCCGTTAA
 Y  Y  S  Q  F  R  -
```

Supplementary Table 9: DNA primer list for the manipulation of *S. cerevisiae*

#	Primer name	Primer Sequence	Use
**74**	*Sc*_Tcd1_F	CCAGGATCCATGAGAGGTTCTCATCATCACCATCATCACGAAAATTTGTATTTTCAAGGTTCTGACCACTTATTTAGGGAAC	Together with primer #5 (Supplementary Table 5) amplification of the His$_6$-TEV-*TCD1*$^{148-1290}$ cassette for insertion into pBEVY-L$^{Amp, Leu}$ via *Bam*HI-HF and *Pst*I-HF
**75**	*Sc*_SUMO^{star} _F	CCAGGTACCATGGGTCATCATCACCATC	Amplification of the His$_6$-SUMO^{star} tag and the His$_6$-SUMO^{star}-*TCD2*$^{184-1344}$ fusion construct for insertion with *Kpn*I-HF into pBEVY-L$^{Amp, Leu}$ *TCD1*$^{148-1290}$
**76**	*Sc*_SUMO^{star} _R	GATTGGCGAATAAACTGTTCCCCACCTATTTGCTCTCTATG	Amplification of the His$_6$-SUMO^{star} tag; overlap with the 5' end of the *TCD2*$^{184-1344}$ gene
**77**	*Sc*_Tcd2 _F	CATAGAGAGCAAATAGGTGGGGAACAGTTTATTCGCCAATC	Amplification of *TCD2*$^{184-1344}$; overlap with the 3' end of the His$_6$-SUMO^{star} gene
**78**	*Sc*_Tcd2_R	CCAGAGCTCTCATCTGAATTGAGAGTAATAAGC	Amplification of *TCD2*$^{184-1344}$, the His$_6$-SUMO^{star}-*TCD2*$^{184-1344}$ and the His$_6$-TEV-*TCD2*$^{184-1344}$ fusion construct for insertion with *Sac*I-HF into pBEVY-L$^{Amp, Leu}$ *TCD1*$^{148-1290}$
**79**	*Sc*_H$_6$TEV_Tcd2_F	CCAGGTACCATGAGAGGTTCTCATCATCACCATCATCACGAAAATTTGTATTTTCAAGGTTCT<br>GAACAGTTTATTCGCCAATC	Amplification of *TCD2*$^{184-1344}$ with a N-terminal His6-TEV tag for insertion with KpnI-HF into pBEVY-L$^{Amp, Leu}$ *TCD1*$^{148-1290}$
**80**	*Sc*_Tcd1_F	AAAAAAAAAGTAAGAATTTTTGAAAACCACCATGGCAAATAATACGTGGAAGTTAATTGCT	Amplification of *TCD1*$^{1-1290}$ for insertion into pBEVY-GL$^{Amp, Leu}$ via Gibson assembly
**81**	*Sc*_Tcd1_R	AAATCATAAATCATAAGAAATTCGCCTAAGAATAGTACTCCTCCTCTTTAAAAAGCCTCT	

**82**	*Sc*_Tcd1_vec_F	GCGAATTTCTTATGATTTATGATTTTTATTATTAAATAAGTTATAAAAA	Amplification of the pBEVY-GL$^{Amp, Leu}$ vector for insertion of *TCD1*$^{1-1290}$ via Gibson assembly
**83**	*Sc*_Tcd1_vec_R	TTTCAAAAATTCTTACTTTTTTTTTGGATGGACGC	
**84**	*Sc*_Tcd2_F	GGTACCGAGCTCGAAACCACCATGGTAGAGAAGGATACATGGAAACTGATAAC	Amplification of *TCD2*$^{1-1344}$ for insertion into pBEVY-GL$^{Amp, Leu}$ via Gibson assembly
**85**	*Sc*_Tcd2_R	GCATAGGAGATCCGCTCATCTGAATTGAGAGTAATAAGCTTCTTCTCTGAAT	
**86**	*Sc*_Tcd2_vec_F	TTCGAGCTCGGTACCCGG	Amplification of the pBEVY-GL$^{Amp, Leu}$ vector for insertion of *TCD2*$^{1-1344}$ via Gibson assembly
**87**	*Sc*_Tcd2_vec_R	GCGGATCTCCTATGCCTTCACG	
**88**	*Sc*_Tcd1R59A_F	ACAATTGGCTgcgAACTATGCATTTCTTGG	Mutagenesis of pBEVY-GL$^{Amp, Leu}$-*TCD1*$^{1-1290}$ –*TCD2*$^{1-1344}$
**89**	*Sc*_Tcd1R59A_R	TCCCTAAATAAGTGGTC	
**90**	*Sc*_Tcd2R128A_F	TTCACTAAATgcgCACAGCTGTGCC	Mutagenesis of pBEVY-GL$^{Amp, Leu}$-*TCD1*$^{1-1290}$ –*TCD2*$^{1-1344}$
**91**	*Sc*_Tcd2R128A_R	CTTAGTGAGACCTGGTC	
**92**	*Sc*_Tcd2K141A_F	TGGCACGCCAgcgGTGGAATGTC	Mutagenesis of pBEVY-GL$^{Amp, Leu}$-*TCD1*$^{1-1290}$ –*TCD2*$^{1-1344}$
**93**	*Sc*_Tcd2K141A_R	ACATCATTTAGTATGGCAC	
**94**	*Sc*_Tcd2R293E_F	CTTCCGTGTAgaaATCCTACCCGTTTTAG	Mutagenesis of pBEVY-GL$^{Amp, Leu}$-*TCD1*$^{1-1290}$ –*TCD2*$^{1-1344}$
**95**	*Sc*_Tcd2R293E_R	TCCTTCAGGGCGCTC	
**96**	*Sc*_Tcd2D188A_F	GGACTGCATTgcgAATATTGATACAAAG	Mutagenesis of pBEVY-GL$^{Amp, Leu}$-*TCD1*$^{1-1290}$ –*TCD2*$^{1-1344}$
**97**	*Sc*_Tcd2D188A_R	ACGATAAAGTCAGGAGTTC	
**98**	*Sc*_Tcd2S285A_F	ACTTCCGTGTAAGAATCCTACCC	Mutagenesis of pBEVY-GL$^{Amp, Leu}$-*TCD1*$^{1-1290}$ –*TCD2*$^{1-1344}$
**99**	*Sc*_Tcd2S285A_R	CCTTCAGGGCggcCAGCTCGTCG	
**100**	*Sc*_Tcd2R291A_F	GAAGGACTTCgcgGTAAGAATCCTACCCGTTTTAGG	Mutagenesis of pBEVY-GL$^{Amp, Leu}$-*TCD1*$^{1-1290}$ –*TCD2*$^{1-1344}$
**101**	*Sc*_Tcd2R291A_R	AGGGCGCTCAGCTCG	

**102**	*Sc*_Tcd2R291E_F	GAAGGACTTCgaaGTAAGAATCCTACCCGTTTTAGG	Mutagenesis of pBEVY-GL$^{Amp, Leu}$-*TCD1*$^{1-1290}$ –*TCD2*$^{1-1344}$
**103**	*Sc*_Tcd2R291E_R	AGGGCGCTCAGCTCG	
**104**	*Sc*_Tcd2L295A_F	TGTAAGAATCgcgCCCGTTTTAGGTAC	Mutagenesis of pBEVY-GL$^{Amp, Leu}$-*TCD1*$^{1-1290}$ –*TCD2*$^{1-1344}$
**105**	*Sc*_Tcd2L295A_R	CGGAAGTCCTTCAGG	
**106**	*Sc*_Tcd1His7_F	TGGTGATGATGAGAATAGTACTCCTCCTC	Tagging of *TCD1* in pBEVY-GL$^{Amp, Leu}$-*TCD1*$^{1-1290}$ – *TCD2*$^{1-1344}$ with a C-terminal His$_7$ tag
**107**	*Sc*_Tcd1His7_R	TCATCACCACTAGGCGAATTTCTTATGATTTATG	
**108**	*Sc*_Tcd2His7_F	ATCATCACCACTGAGCGGATCTCCTATG	Tagging of *TCD2* in pBEVY-GL$^{Amp, Leu}$-*TCD1*$^{1-1290}$ – *TCD2*$^{1-1344}$ with a C-terminal His$_7$ tag
**109**	*Sc*_Tcd2His7_R	GGTGATGATGTCTGAATTGAGAGTAATAAGC	
**110**	Delta_TCD1 _F	AGTAAAGTATTAATACTGCAATTAGTTAGGATAGCAGAACGAACAACGTACGCTGCAGGTCGAC	Knockout of *TCD1*; overlap with the NatMX cassette of pFA6a-NatMX6
**111**	Delta_TCD1_R	GTAAATAGTATTAATAAATCTATATTCAATATGTTGTATTTTTGATCTCCCATTGATCGATGAATTCGAGCTCG	
**112**	Delta_Cterm_TCD1_F	GACATGGATTTTAACCAAAGTGTCAGGCTATCCAATGAAAGAAAATGAGGTC**TAG**GGCGCGCCACTTCTAAATAAG	Knockout of C-terminal domain of *TCD1*; overlap with the *KanMX4* cassette of pYM14-*KanMX4* (Supplementary Table 10)
**113**	Delta_Cterm_TCD1_R	GTAAATAGTATTAATAAATCTATATTCAATATGTTGTATTTTTGATCTCCCATTGGAATTCGAGCTCGTTTAAACT	
**114**	Delta_Cterm_TCD2_F	GTGGATTTTATCTAATATATCCGATAAACCTCTAGAACCTGTTGAGGGT**TAG**CGTACGCTGCAGGTCGAC	Knockout of C-terminal domain of TCD2; overlap with the *NatMX6* cassette of pFA6a-*NatMX6* (Supplementary Table 10)
**115**	Delta_Cterm_TCD2_R	GGAATAGTACGTAACTCTATGGAAGAAAATGCTTTATATGTTGGAAAGGAACAAGAATCGATGAATTCGAGCTCG	

**116**	TCD1_up_F	TTGCGTTCTAAGGGATGG TG	Binds upstream of *TCD1* in *S. cerevisiae* genome; control for *TCD1* deletion
**117**	TCD2_up_F	GAAACTGCTAGAGAAAAT GCATTGG	Binds upstream of *TCD2* in *S. cerevisiae* genome; control for *TCD2* deletion
**118**	TCD1_Cterm_up_ F	ATGGGTGTTGCCACGAA ATC	Binds upstream of the C-terminal domain of *TCD1*; control for C-terminal deletion/ tagging
**119**	TCD2_Cterm_up_ F	ATGGCGAAAGACTGACC CTTG	Binds upstream of the C-terminal domain of *TCD2*; control for C-terminal deletion/ tagging
**120**	Kan_R	GTATTCTGGGCCTCCATG TC	Binds within the *KanMX* cassette
**121**	Nat_R	GTGGTGAAGGACCCATC CAG	Binds within the *NatMX*/*NT2* cassette
**122**	TCD1-tag_F	GTTCTCGATTTTATTATTG AGAGGCTTTTTATTGAGA GGCTTTTTAAAGAGGAGG AGTACTATTCTCGTACGC TCAGGTCGACTC	C-terminal tagging of Tcd1 with either a HA_6 tag or a Myc_9 tag; overlap with corresponding tagging and resistance cassette on pYM14 and pYM21 (Supplementary Table 10)
**123**	TCD1-tag_R	GTAAATAGTATTAATAAAT CTATATTCAATATGTTGTA TTTTTGATCTCCCATTGAT CGATGAATTCGAGCTCG	
**124**	TCD2-tag_F	CTGGTTTCACAAAGATTC AGAGAAGAAGCTTATTAC TCTCAATTCAGACGTACG CTGCAGGTCGACTC	C-terminal tagging of Tcd1 with either a HA_6 tag or a Myc_9 tag; overlap with corresponding tagging and resistance cassette on pYM14 and pYM21 (Supplementary Table 10)
**125**	TCD2-tag_R	GTACGTAACTCTATGGAA GAAAATGCTTTATATGTT GGAAAGGAACAAGAATC GATGAATTCGAGCTCG	

Underlined bases represent recognition sites for restriction endonucleases used for cloning. Italic bases denote restriction sites that could not be used for cloning here. Lower case bases represent site of mutations. Bold bases are stop codons introduced.

Supplementary Table 10: Yeast plasmids used

Plasmid name	Description
pBEVY-L$^{Amp, Leu}$ (14)	bi-directional expression vector for yeast, constitutively active
pBEVY-GL$^{Amp, Leu}$ (14)	bi-directional expression vector for yeast, galactose inducible
pBEVY-L$^{Amp, Leu}$-His$_6$-TEV-*TCD1*$^{148-1290}$–His$_6$-SUMO^{star}-*TCD2*$^{184-1344}$	Overproduction of His$_6$-TEV-Tcd1$^{50-429}$ and His$_6$-SUMO^{star}-Tcd2$^{62-447}$ in *S. cerevisiae*
pBEVY-(G)L$^{Amp, Leu}$-His$_6$-TEV-*TCD1*$^{148-1290}$–His$_6$-TEV-*TCD2*$^{184-1344}$	Overproduction of His$_6$-TEV-Tcd1$^{50-429}$ and His$_6$-TEV-Tcd2$^{62-447}$ in *S. cerevisiae*
pBEVY-GL$^{Amp, Leu}$-*TCD1*$^{1-1290}$–*TCD2*$^{1-1344}$	Overproduction of full-length and tag-free Tcd1$^{1-429}$ and Tcd2$^{1-447}$ in *S. cerevisiae*
pBEVY-GL$^{Amp, Leu}$-*TCD1*$^{1-1290}_{R59A}$–*TCD2*$^{1-1344}$	Overproduction of full-length and tag-free Tcd1$^{1-429}$-R59A and Tcd2$^{1-447}$ in *S. cerevisiae*
pBEVY-GL$^{Amp, Leu}$-*TCD1*$^{1-1290}$–*TCD2*$^{1-1344}_{K141A}$	Overproduction of full-length and tag-free Tcd1$^{1-429}$ and Tcd2$^{1-447}$-K141A in *S. cerevisiae*
pBEVY-GL$^{Amp, Leu}$-*TCD1*$^{1-1290}$–*TCD2*$^{1-1344}_{R128A}$	Overproduction of full-length and tag-free Tcd1$^{1-429}$ and Tcd2$^{1-447}$-R128A in *S. cerevisiae*
pBEVY-GL$^{Amp, Leu}$-*TCD1*$^{1-1290}$–*TCD2*$^{1-1344}_{R293E}$	Overproduction of full-length and tag-free Tcd1$^{1-429}$ and Tcd2$^{1-447}$-R293E in *S. cerevisiae*
pBEVY-GL$^{Amp, Leu}$-*TCD1*$^{1-1290}$–*TCD2*$^{1-1344}_{D188A}$	Overproduction of full-length and tag-free Tcd1$^{1-429}$ and Tcd2$^{1-447}$-D188A in *S. cerevisiae*
pBEVY-GL$^{Amp, Leu}$-*TCD1*$^{1-1290}$–*TCD2*$^{1-1344}_{S285A}$	Overproduction of full-length and tag-free Tcd1$^{1-429}$ and Tcd2$^{1-447}$-S285A in *S. cerevisiae*
pBEVY-GL$^{Amp, Leu}$-*TCD1*$^{1-1290}$–*TCD2*$^{1-1344}_{R291A}$	Overproduction of full length and tag-free Tcd1$^{1-429}$ and Tcd2$^{1-447}$-R291A in *S. cerevisiae*
pBEVY-GL$^{Amp, Leu}$-*TCD1*$^{1-1290}$–*TCD2*$^{1-1344}_{R291E}$	Overproduction of full length and tag-free Tcd1$^{1-429}$ and Tcd2$^{1-447}$-R291E in *S. cerevisiae*
pBEVY-GL$^{Amp, Leu}$-*TCD1*$^{1-1290}$–*TCD2*$^{1-1344}_{L295A}$	Overproduction of full length and tag-free Tcd1$^{1-429}$ and Tcd2$^{1-447}$-L295A in *S. cerevisiae*
pBEVY-GL$^{Amp, Leu}$-*TCD1*$^{1-1290}$-*His*$_7$–*TCD2*$^{1-1344}$	Overproduction of full length Tcd1$^{1-429}$-His$_7$ and Tcd2$^{1-447}$ in *S. cerevisiae*
pBEVY-GL$^{Amp, Leu}$-*TCD1*$^{1-1290}$–*TCD2*$^{1-1344}$-His$_7$	Overproduction of full length Tcd1$^{1-429}$ and Tcd2$^{1-447}$-His$_7$ in *S. cerevisiae*
pBEVY-GL$^{Amp, Leu}$-*TCD1*$^{1-1290}_{R59A}$-*His*$_7$–*TCD2*$^{1-1344}$	Overproduction of full length Tcd1$^{1-429}$-R59A and Tcd2$^{1-447}$ in *S. cerevisiae*
pBEVY-GL$^{Amp, Leu}$-*TCD1*$^{1-1290}$–*TCD2*$^{1-1344}_{K141A}$-His$_7$	Overproduction of full length Tcd1$^{1-429}$ and Tcd2$^{1-447}$-K141A-His$_7$ in *S. cerevisiae*

pBEVY-GL$^{Amp, Leu}$-*TCD1*$^{1\text{-}1290}$–*TCD2*$^{1\text{-}1344}_{R128A}$-His$_7$	Overproduction of full length and tag-free Tcd1$^{1\text{-}429}$ and Tcd2$^{1\text{-}447}$-R128A-His$_7$ in *S. cerevisiae*
pBEVY-GL$^{Amp, Leu}$-*TCD1*$^{1\text{-}1290}$–*TCD2*$^{1\text{-}1344}_{R293E}$-His$_7$	Overproduction of full length and tag-free Tcd1$^{1\text{-}429}$ and Tcd2$^{1\text{-}447}$-R293E-His$_7$ in *S. cerevisiae*
pBEVY-GL$^{Amp, Leu}$-*TCD1*$^{1\text{-}1290}$–*TCD2*$^{1\text{-}1344}_{D188A}$-His$_7$	Overproduction of full length and tag-free Tcd1$^{1\text{-}429}$ and Tcd2$^{1\text{-}447}$-D188A-His$_7$ in *S. cerevisiae*
pBEVY-GL$^{Amp, Leu}$-*TCD1*$^{1\text{-}1290}$–*TCD2*$^{1\text{-}1344}_{S285A}$-His$_7$	Overproduction of full length and tag-free Tcd1$^{1\text{-}429}$ and Tcd2$^{1\text{-}447}$-S285A-His$_7$ in *S. cerevisiae*
pBEVY-GL$^{Amp, Leu}$-*TCD1*$^{1\text{-}1290}$–*TCD2*$^{1\text{-}1344}_{R291A}$-His$_7$	Overproduction of full length and tag-free Tcd1$^{1\text{-}429}$ and Tcd2$^{1\text{-}447}$-R291A-His$_7$ in *S. cerevisiae*
pBEVY-GL$^{Amp, Leu}$-*TCD1*$^{1\text{-}1290}$–*TCD2*$^{1\text{-}1344}_{R291E}$-His$_7$	Overproduction of full length and tag-free Tcd1$^{1\text{-}429}$ and Tcd2$^{1\text{-}447}$-R291E-His$_7$ in *S. cerevisiae*
pBEVY-GL$^{Amp, Leu}$-*TCD1*$^{1\text{-}1290}$–*TCD2*$^{1\text{-}1344}_{L295A}$-His$_7$	Overproduction of full length and tag-free Tcd1$^{1\text{-}429}$ and Tcd2$^{1\text{-}447}$-L295A-His$_7$ in *S. cerevisiae*
pFA6a-*NatMX6*	Template plasmid for amplification of *NatMX6* cassette
pYM14	Template plasmid for amplification of HA$_6$ tag and *KanMX4* cassette
pYM21	Template plasmid for amplification of Myc$_9$ tag and *NatNT2* cassette

Supplementary Table 11: Genotype and origin of *E. coli* and S*. cerevisiae* strains used

Strain	Genotype	Company
E. coli XL1blue	*recA1 endA1 gyrA96 thi-1 hsdR17 supE44 relA1 lac* [F´ *proAB lacIq Z∆M15* Tn*10* (Tetr)]	Agilent Techonolgies (St. Clara, US)
E. coli BL21 (DE3)	*E. coli* str. B F$^-$ *ompT gal dcm lon* $hsdS_B(r_B^-m_B^-)$ λ(DE3 [*lacI lacUV5-T7p07 ind1 sam7 nin5*]) $[malB^+]_{K\text{-}12}(\lambda^S)$	Novagen/Merck (Darmstadt, DE)
E. coli SoluBL21	*E. coli* str. B F$^-$ *ompT* $hsdS_B(r_B^-m_B^-)$ *gal dcm lon* λ(DE3 [*lacI lacUV5-T7p07 ind1 sam7 nin5*])*	Genlantis (San Diego, US)
S. cerevisiae WCG4a	*MAT***<u>a</u>** *leu2-3,112 ura3 his3-11,15 CanS GAL2*	Heinemeyer *et al., 1993*
S. cerevisiae BY4741	*MAT***a** *his3Δ1 leu2Δ0 met15Δ0 ura3Δ0*	Euroscarf (Oberursel, DE)
S. cerevisiae BY4742	*MAT***α** *his3Δ1 leu2Δ0 lys2Δ0 ura3Δ0*	Euroscarf (Oberursel, DE)
S. cerevisiae BY4741 *Δtcd1*	*MAT***a** *his3Δ1 leu2Δ0 met15Δ0 ura3Δ0 YHR003c::KanMX4*	Euroscarf (Oberursel, DE)
S. cerevisiae BY4741 *Δtcd2*	*MAT***a** *his3Δ1 leu2Δ0 met15Δ0 ura3Δ0 YKL027w::KanMX4*	Euroscarf (Oberursel, DE)
S. cerevisiae BY4742 *Δtcd1*	*MAT***α** *his3Δ1 leu2Δ0 lys2Δ0 ura3Δ0 YHR003c::NatMX6*	This work
S. cerevisiae BY4741 *Δtcd1 Δtcd2*	*MAT***a** *his3Δ1 leu2Δ0 met15Δ0 ura3Δ0 YHR003c::NatMX6 YKL027w::KanMX4*	This work
S. cerevisiae BY4741 *tcd1ΔCterm*	*MAT***a** *his3Δ1 leu2Δ0 met15Δ0 ura3Δ0 YHR003c-ΔCterm::KanMX4*	This work
S. cerevisiae BY4741 *tcd2ΔCterm*	*MAT***a** *his3Δ1 leu2Δ0 met15Δ0 ura3Δ0 YKL027w-ΔCterm::NatMX6*	This work
S. cerevisiae BY4741 *tcd1ΔCterm tcd2ΔCterm*	*MAT***a** *his3Δ1 leu2Δ0 met15Δ0 ura3Δ0 YHR003c-ΔCterm::KanMX4 YKL027w-ΔCterm::NatMX6*	This work
S. cerevisiae BY4741-*tcd1*-Myc$_9$ *tcd2*-HA$_6$	*MAT***a** *his3Δ1 leu2Δ0 met15Δ0 ura3Δ0 YHR003c-Myc*$_9$*::NatNT2, YKL027w-HA*$_6$*::KanMX4*	This work
S. cerevisiae BY4741- *tcd1*-HA$_6$, *tcd2* -Myc$_9$	*MAT***a** *his3Δ1 leu2Δ0 met15Δ0 ura3Δ0 YHR003c-HA*$_6$*::KanMX4, YKL027w-Myc*$_9$*::NatNT2*	This work

*According to the company, some uncharacterized mutations in this *E. coli* strain are essential for its specific properties.

S. cerevisiae RS001 (BY4741)	*MAT**a** his3Δ1 leu2Δ0 met15Δ0 ura3Δ0 YHR003c::NatMX6 YKL027w::KanMX4* [pBEVY-GL-His$_6$-TEV-YHR003c$^{148-1290}$-His$_6$-SUMO^{star}-YKL027w$^{184-1344}$]	This work
S. cerevisiae RS002 (BY4741)	*MAT**a** his3Δ1 leu2Δ0 met15Δ0 ura3Δ0 YHR003c::NatMX6 YKL027w::KanMX4* [pBEVY-GL-YHR003c$^{1-1290}$-YKL027w$^{1-1344}$]	This work
S. cerevisiae RS003 (BY4741)	*MAT**a** his3Δ1 leu2Δ0 met15Δ0 ura3Δ0 YHR003c::NatMX6 YKL027w::KanMX4* [pBEVY-GL-YHR003c$^{1-1290}{}_{R59A}$-YKL027w$^{1-1344}$]	This work
S. cerevisiae RS004 (BY4741)	*MAT**a** his3Δ1 leu2Δ0 met15Δ0 ura3Δ0 YHR003c::NatMX6 YKL027w::KanMX4* [pBEVY-GL-YHR003c$^{1-1290}$-YKL027w$^{1-1344}{}_{K141A}$]	This work
S. cerevisiae RS005 (BY4741)	*MAT**a** his3Δ1 leu2Δ0 met15Δ0 ura3Δ0 YHR003c::NatMX6 YKL027w::KanMX4* [pBEVY-GL-YHR003c$^{1-1290}$-YKL027w$^{1-1344}{}_{R128A}$]	This work
S. cerevisiae RS006 (BY4741)	*MAT**a** his3Δ1 leu2Δ0 met15Δ0 ura3Δ0 YHR003c::NatMX6 YKL027w::KanMX4* [pBEVY-GL-YHR003c$^{1-1290}$-YKL027w$^{1-1344}{}_{R293E}$]	This work
S. cerevisiae RS007 (BY4741)	*MAT**a** his3Δ1 leu2Δ0 met15Δ0 ura3Δ0 YHR003c::NatMX6 YKL027w::KanMX4* [pBEVY-GL-YHR003c$^{1-1290}$-YKL027w$^{1-1344}{}_{D188A}$]	This work
S. cerevisiae RS008 (BY4741)	*MAT**a** his3Δ1 leu2Δ0 met15Δ0 ura3Δ0 YHR003c::NatMX6 YKL027w::KanMX4* [pBEVY-GL-YHR003c$^{1-1290}$-YKL027w$^{1-1344}{}_{S285A}$]	This work
S. cerevisiae RS009 (BY4741)	*MAT**a** his3Δ1 leu2Δ0 met15Δ0 ura3Δ0 YHR003c::NatMX6 YKL027w::KanMX4* [pBEVY-GL-YHR003c$^{1-1290}$-YKL027w$^{1-1344}{}_{R291A}$]	This work
S. cerevisiae RS010 (BY4741)	*MAT**a** his3Δ1 leu2Δ0 met15Δ0 ura3Δ0 YHR003c::NatMX6 YKL027w::KanMX4* [pBEVY-GL-YHR003c$^{1-1290}$-YKL027w$^{1-1344}{}_{R291E}$]	This work
S. cerevisiae RS011 (BY4741)	*MAT**a** his3Δ1 leu2Δ0 met15Δ0 ura3Δ0 YHR003c::NatMX6 YKL027w::KanMX4* [pBEVY-GL-YHR003c$^{1-1290}$-YKL027w$^{1-1344}{}_{L295A}$]	This work

S. cerevisiae RS012 (BY4741)	*MAT**a** his3Δ1 leu2Δ0 met15Δ0 ura3Δ0 YHR003c::NatMX6 YKL027w::KanMX4* [pBEVY-GL-YHR003c$^{1-1290}$-His$_7$-YKL027w$^{1-1344}$]	This work
S. cerevisiae RS013 (BY4741)	*MAT**a** his3Δ1 leu2Δ0 met15Δ0 ura3Δ0 YHR003c::NatMX6 YKL027w::KanMX4* [pBEVY-GL-YHR003c$^{1-1290}$-YKL027w$^{1-1344}$-His$_7$]	This work
S. cerevisiae RS014 (BY4741)	*MAT**a** his3Δ1 leu2Δ0 met15Δ0 ura3Δ0 YHR003c::NatMX6 YKL027w::KanMX4* [pBEVY-GL-YHR003c$^{1-1290}_{R59A}$-His$_7$-YKL027w$^{1-1344}$]	This work
S. cerevisiae RS015 (BY4741)	*MAT**a** his3Δ1 leu2Δ0 met15Δ0 ura3Δ0 YHR003c::NatMX6 YKL027w::KanMX4* [pBEVY-GL-YHR003c$^{1-1290}$-YKL027w$^{1-1344}_{K141A}$-His$_7$]	This work
S. cerevisiae RS016 (BY4741)	*MAT**a** his3Δ1 leu2Δ0 met15Δ0 ura3Δ0 YHR003c::NatMX6 YKL027w::KanMX4* [pBEVY-GL-YHR003c$^{1-1290}$-YKL027w$^{1-1344}_{R128A}$-His$_7$]	This work
S. cerevisiae RS017 (BY4741)	*MAT**a** his3Δ1 leu2Δ0 met15Δ0 ura3Δ0 YHR003c::NatMX6 YKL027w::KanMX4* [pBEVY-GL-YHR003c$^{1-1290}$-YKL027w$^{1-1344}_{R293E}$-His$_7$]	This work
S. cerevisiae RS018 (BY4741)	*MAT**a** his3Δ1 leu2Δ0 met15Δ0 ura3Δ0 YHR003c::NatMX6 YKL027w::KanMX4* [pBEVY-GL-YHR003c$^{1-1290}$-YKL027w$^{1-1344}_{D188A}$-His$_7$]	This work
S. cerevisiae RS019 (BY4741)	*MAT**a** his3Δ1 leu2Δ0 met15Δ0 ura3Δ0 YHR003c::NatMX6 YKL027w::KanMX4* [pBEVY-GL-YHR003c$^{1-1290}$-YKL027w$^{1-1344}_{S285A}$-His$_7$]	This work
S. cerevisiae RS020 (BY4741)	*MAT**a** his3Δ1 leu2Δ0 met15Δ0 ura3Δ0 YHR003c::NatMX6 YKL027w::KanMX4* [pBEVY-GL-YHR003c$^{1-1290}$-YKL027w$^{1-1344}_{R291A}$-His$_7$]	This work
S. cerevisiae RS021 (BY4741)	*MAT**a** his3Δ1 leu2Δ0 met15Δ0 ura3Δ0 YHR003c::NatMX6 YKL027w::KanMX4* [pBEVY-GL-YHR003c$^{1-1290}$-YKL027w$^{1-1344}_{R291E}$-His$_7$]	This work
S. cerevisiae RS022 (BY4741)	*MAT**a** his3Δ1 leu2Δ0 met15Δ0 ura3Δ0 YHR003c::NatMX6 YKL027w::KanMX4* [pBEVY-GL-YHR003c$^{1-1290}$-YKL027w$^{1-1344}_{L295A}$-His$_7$]	This work

Supplementary Table 12: Solutions for *S. cerevisiae* transformation

Buffer	Composition
10× TE	100 mM Tris/HCl pH 7.5, 10 mM EDTA pH 8.0
10× LiAc	1 M Lithium acetate, 2*H_2O
PEG_{3350}	50% (v/v) PEG_{3350}
YPD	2% (w/v) Peptone, 1% (w/v) Yeast extract, 2% (w/v) Glucose, (2% (w/v) Agar$_{(only\ for\ plates)}$)
YPG	2% (w/v) Peptone, 1% (w/v) Yeast extract, 3% (w/v) Glycerol, (2% (w/v) Agar$_{(only\ for\ plates)}$)

Supplementary Table 13: Buffers for Western Blotting

Buffer	Composition
Electrophoresis buffer (1×)	25 mM Tris, 192 mM Gylcine, 0.1% (w/v) SDS
Tris-buffered saline (TBS)	50 mM Tris/HCl pH 7.2, 150 mM NaCl
TBS-Tween (TBS-T)	50 mM Tris/HCl pH 7.2, 150 mM NaCl, 0.1% (v/v) Tween20
Transfer Buffer	80% (v/v) Electrophoresis buffer (1×), 20% (v/v) Methanol

Supplementary Table 14: Antibodies

Antibody and used dilution	Supplier
HA Tag monoclonal antibody (mouse) (1:10,000) (AB_10978021)	Invitrogen (Carlsbad, US)
Myc Tag monoclonal antibody (mouse) (1:1,000) (AB_558473)	Invitrogen (Carlsbad, US)
VDAC-1 (Porin) monoclonal antibody (mouse) (1:1,000) (16G9E6BC4)	Invitrogen (Carlsbad, US)
Actin monoclonal antibody (mouse) (1:5,000) (mAbGEa)	Invitrogen (Carlsbad, US)
HRP-conjugated anti-Mouse IgG (goat) Cross-Adsorbed Secondary Antibody (1:20,000) (AB_2536527)	Invitrogen (Carlsbad, US)

HRP-conjugated anti-Mouse IgG (rabbit) secondary antibody (1:10,000) (A9044)	Merck (Darmstadt, DE)
HRP-conjugated 6*His-Tag Monoclonal antibody (1:5,000) (HRP-66005)	Proteintech (Rosemont, US)
Multiple Tags positive control (GST-DDDDK-V5-HSV-HA-T7-Myc-S)	GeneTex (Irvine, US)

Supplementary Table 15: Synthetic *S. cerevisiae* tRNA genes for *in vitro* transcription

tRNA gene name	tRNA gene sequence
$tRNA^{Lys(UUU)}$	GCAGAGTAATACGACTCACTATAGGGTCCTTGTTAGCTCAGTTGGTAGAGCGTTCGGC**TTT**TAAGCGCATTTGCTTAAGCAAGGATACCGAAATGTCAGGGGTTCGAGCCCCCTATGAGGAGCCA
$tRNA^{Met(CAU)}$	GCAGAGTAATACGACTCACTATAGGGGCTTCAGTAGCTCAGTAGGAAGAGCGTCAGTCT**CAT**AATCTGAAGGTCGAGAGTTCGAACCTCCCCTGGAGCACCA
$tRNA^{Thr(AGU)}$	GCAGAGTAATACGACTCACTATAGGGGCTTCTATGGCCAAGTTGGTAAGGCGCCACACT**AGT**AATGTGGAGATCATCGGTTCAAATCCGATTGGAAGCACCA

Sequences encoding the *T7* promoter and CCA tail are underlined, while that for the anticodon is printed bold.

Supplementary Table 16: Complete minimal dropout media

Buffer	Composition
Complete minimal $medium^{Leu-}$ (CM^{Leu-})	20% (w/v) Glucose, 6.67% (w/v) Yeast Nitrogen Base w/o amino acids, 2 g/l Dropout Mix, 0.024 g/l L-Histidine
Galactose selection $medium^{Leu-}$	6.67% (w/v) Yeast Nitrogen Base w/o amino acids, 2 g/l Dropout Mix, 2% (v/v) Galactose, 0.024 g/l L-Histidine
Dropout Mix	2.00 g L-Alanine, 2.00 g L-Arginine, 2.00 g L-Asparagine-Monohydrate, 2.00 g L-Aspartic acid, 2.00 g L-Cysteine, 2.00 g L-Glutamine, 2.00 g L-Glutamic acid, 2.00 g L-Glycine, 2.00 g L-Lysin, 2.00 g L-Methionine, 2.00 g L-Isoleucine, 2.00 g L-Phenylalanine, 2.00 g L-Proline, 2.00 g L-Serin, 2.00 g L-Threonine, 2.00 g L-Tryptophan, 2.00 g L-Tyrosine, 2.00 g L-Valine, 2.00 g Myo-Inositol, 0.20 g PABA (4-Aminobenzoic acid), 0.50 g Adenine-Hemisulfate

Supplementary Table 17: Buffers for protein purification

Protein	Buffer	Composition
Tcd	Buffer A^{Tcd}	100 mM Tris/HCl pH 7.5, 50 mM NaCl, 5 mM $MgCl_2$, 20 mM Imidazole, 10% (w/v) Glycerol, 3 mM β-Mercaptoethanol
	Buffer B^{Tcd}	100 mM Tris/HCl pH 7.5, 50 mM NaCl, 5 mM $MgCl_2$, 500 mM Imidazole, 10% (w/v) Glycerol, 3 mM β-Mercaptoethanol
	Buffer C^{Tcd}	50 mM Tris/HCl pH 7.5, 150 mM NaCl, 5 mM $MgCl_2$, 10% (w/v) Glycerol, 3 mM β-Mercaptoethanol
	Buffer D^{Tcd}	20 mM Tris/HCl pH 7.5, 150 mM NaCl, 5 mM $MgCl_2$, 10% (w/v) Glycerol, 3 mM DTT
$Tcd^{366-485}$	Buffer $A^{Tcd366-485}$	100 mM Tris/HCl pH 7.5, 50 mM NaCl, 20 mM Imidazole, 10% (w/v) Glycerol, 3 mM β-Mercaptoethanol
	Buffer $B^{Tcd366-485}$	100 mM Tris/HCl pH 7.5, 50 mM NaCl, 500 mM Imidazole, 10% (w/v) Glycerol, 3 mM β-Mercaptoethanol
	Buffer $C^{Tcd366-485}$	50 mM Tris/HCl pH 7.5, 150 mM NaCl, 10% (w/v) Glycerol, 3 mM β-Mercaptoethanol
	Buffer $D^{Tcd366-485}$	20 mM Tris/HCl pH 7.5, 150 mM NaCl, 10% (w/v) Glycerol, 3 mM DTT
Tcd1&Tcd2 – initial buffers	Buffer $A^{Tcd1\&Tcd2}$	100 mM Tris pH 7.5, 50 mM NaCl, 20 mM Imidazole, 10% (w/v) Glycerol, 3 mM β-Mercaptoethanol
	Buffer $B^{Tcd1\&Tcd2}$	100 mM Tris pH 7.5, 50 mM NaCl, 500 mM Imidazole, 10% (w/v) Glycerol, 3 mM β-Mercaptoethanol
	Buffer $C^{Tcd1\&Tcd2}$	50 mM Tris pH 7.5, 150 mM NaCl, 10% (w/v) Glycerol, 3 mM β-Mercaptoethanol
	Buffer $D^{Tcd1\&Tcd2}$	20 mM Tris pH 7.5, 150 mM NaCl, 10% (w/v) Glycerol, 3 mM DTT
Tcd1&Tcd2 – optimized buffers	Buffer $A^{Tcd1\&Tcd2}$	100 mM Bis-Tris/HCl pH 6.5, 50 mM NaCl, 5 mM $MgCl_2$, 20 mM Imidazole, 10% (w/v) Glycerol, 3 mM β-Mercaptoethanol
	Buffer $B^{Tcd1\&Tcd2}$	100 mM Bis-Tris/HCl pH 6.5, 50 mM NaCl, 5 mM $MgCl_2$, 500 mM Imidazole, 10% (w/v) Glycerol, 3 mM β-Mercaptoethanol
	Buffer $C^{Tcd1\&Tcd2}$	50 mM Bis-Tris/HCl pH 6.5, 150 mM NaCl, 5 mM $MgCl_2$, 10% (w/v) Glycerol, 3 mM β-Mercaptoethanol
	Buffer $D^{Tcd1\&Tcd2}$	20 mM Bis-Tris/HCl pH 6.5, 150 mM NaCl, 5 mM $MgCl_2$, 10% (w/v) Glycerol, 3 mM DTT
TcdA low salt	Buffer A^{low}	100 mM Bis-Tris/HCl pH 7.5, 50 mM NaCl, 5 mM $MgCl_2$, 20 mM Imidazole, 10% (w/v) Glycerol, 3 mM β-Mercaptoethanol
	Buffer B^{low}	100 mM Bis-Tris/HCl pH 6.5, 50 mM NaCl, 5 mM $MgCl_2$, 500 mM Imidazole, 10% (w/v) Glycerol, 3 mM β-Mercaptoethanol

	Buffer C^{low}	50 mM Bis-Tris/HCl pH 7.5, 150 mM NaCl, 2 mM $MgCl_2$, 10% (w/v) Glycerol, 3 mM β-Mercaptoethanol
	Buffer D^{low}	20 mM Bis-Tris/HCl pH 7.5, 150 mM NaCl, 5 mM $MgCl_2$, 10% (w/v) Glycerol, 3 mM DTT
TcdA high salt	Buffer A^{high}	50 mM Sodium phosphate pH 7.5, 300 mM NaCl, 20 mM Imidazole, 10% (w/v) Glycerol, 2 mM β-Mercaptoethanol
	Buffer B^{high}	50 mM Sodium phosphate pH 7.5, 300 mM NaCl, 500 mM Imidazole, 10% (w/v) Glycerol, 2 mM β-Mercaptoethanol
	Buffer C^{high}	20 mM Sodium phosphate pH 7.5, 300 mM NaCl, 10% (w/v) Glycerol, 2 mM β-Mercaptoethanol
	Buffer D^{high}	20 mM Sodium phosphate pH 7.5, 300 mM NaCl, 10% (w/v) Glycerol, 3 mM DTT

Supplementary Table 18: Buffers for native PAGE/EMSA

Buffer	Composition
Binding Buffer	20% (w/v) Glycerol, 50 mM Tris/HCl pH 7.5, 5 mM $MgCl_2$, 2.5 mM EDTA, 2.5 mM DTT, 250 mM NaCl
Loading Buffer	40% (w/v) Glycerol, 250 mM Tris/HCl pH 7.5, 1 mg Bromophenol blue, 3 µL Xylencyanol
TBE Buffer (5×) pH 8.3	450 mM Tris, 444 mM Boric acid, 10 mM EDTA

SUPPLEMENTARY REFERENCES

1. Jinek, M., Jiang, F., Taylor, D.W. *et al.* Structures of Cas9 endonucleases reveal RNA-mediated conformational activation. *Science* 2014;343:1247997.
2. Shen, B.W., Heiter, D.F., Chan, S.H. *et al.* Unusual target site disruption by the rare-cutting HNH restriction endonuclease PacI. *Structure* 2010;18:734–743.
3. Lopez-Estepa, M., Arda, A., Savko, M. *et al.* The crystal structure and small-angle X-ray analysis of CsdL/TcdA reveal a new tRNA binding motif in the MoeB/E1 superfamily. *PloS one* 2015;10:e0118606.
4. Liebschner, D., Afonine, P.V., Baker, M.L. *et al.* Macromolecular structure determination using X-rays, neutrons and electrons: recent developments in Phenix. *Acta Crystallogr D Struct Biol* 2019;75:861–877.
5. Abramson, J., Adler, J., Dunger, J. *et al.* Accurate structure prediction of biomolecular interactions with AlphaFold 3. *Nature* 2024;630:493–500.
6. Duda, D.M., Walden, H., Sfondouris, J. *et al.* Structural analysis of Escherichia coli ThiF. *J Mol Biol* 2005;349:774–786.
7. Xi, J., Ge, Y., Kinsland, C. *et al.* Biosynthesis of the thiazole moiety of thiamin in Escherichia coli: identification of an acyldisulfide-linked protein--protein conjugate that is functionally analogous to the ubiquitin/E1 complex. *Proc Natl Acad Sci U S A* 2001;98:8513–8518.
8. Tripos. 1699 South Hanley Rd., St. Louis, Missouri, 63144, USA
9. Emsley, P., Lohkamp, B., Scott, W.G. *et al.* Features and development of Coot. *Acta Crystallogr D Biol Crystallogr* 2010;66:486–501.
10. Leimkuhler, S., Wuebbens, M.M. and Rajagopalan, K.V. Characterization of Escherichia coli MoeB and its involvement in the activation of molybdopterin synthase for the biosynthesis of the molybdenum cofactor. *J Biol Chem* 2001;276:34695–34701.
11. Kim, S., Lee, H. and Park, S. The Structure of Escherichia coli TcdA (Also Known As CsdL) Reveals a Novel Topology and Provides Insight into the tRNA Binding Surface Required for N(6)-Threonylcarbamoyladenosine Dehydratase Activity. *J Mol Biol* 2015;427:3074–3085.
12. Holm, L. and Rosenstrom, P. Dali server: conservation mapping in 3D. *Nucleic acids research* 2010;38:W545–549.
13. Holm, L., Kaariainen, S., Rosenstrom, P. *et al.* Searching protein structure databases with DaliLite v.3. *Bioinformatics* 2008;24:2780–2781.
14. Miller, C.A., 3rd, Martinat, M.A. and Hyman, L.E. Assessment of aryl hydrocarbon receptor complex interactions using pBEVY plasmids: expressionvectors with bi-directional promoters for use in Saccharomyces cerevisiae. *Nucleic Acids Res* 1998;26:3577–3583.
